# Development of prognostic model for preterm birth using machine learning in a population-based cohort of Western Australia births between 1980 and 2015

**DOI:** 10.1038/s41598-022-23782-w

**Published:** 2022-11-09

**Authors:** Kingsley Wong, Gizachew A. Tessema, Kevin Chai, Gavin Pereira

**Affiliations:** 1 Curtin School of Population Health, Curtin University, 400 Kent St, Bentley, Perth, WA 6102 Australia; 2 Telethon Kids Institute, The University of Western Australia, Perth, WA Australia; 3 School of Public Health, University of Adelaide, Adelaide, South Australia Australia; 4 enAble Institute, Curtin University, Perth, WA Australia; 5 Centre for Fertility and Health (CeFH), Norwegian Institute of Public Health, Oslo, Norway

**Keywords:** Population screening, Health services

## Abstract

Preterm birth is a global public health problem with a significant burden on the individuals affected. The study aimed to extend current research on preterm birth prognostic model development by developing and internally validating models using machine learning classification algorithms and population-based routinely collected data in Western Australia. The longitudinal retrospective cohort study involved all births in Western Australia between 1980 and 2015, and the analytic sample contains 81,974 (8.6%) preterm births (< 37 weeks of gestation). Prediction models for preterm birth were developed using regularised logistic regression, decision trees, Random Forests, extreme gradient boosting, and multi-layer perceptron (MLP). Predictors included maternal socio-demographics and medical conditions, current and past pregnancy complications, and family history. Class weight was applied to handle imbalanced outcomes and stratified tenfold cross-validation was used to reduce overfitting. Close to half of the preterm births (49.1% at 5% FPR, 95% CI 48.9%,49.5%) were correctly classified by the best performing classifier (MLP) for all women when current pregnancy information was available. The sensitivity was boosted to 52.7% (95% CI 52.1%,53.3%) after including past obstetric history in a sub-population of births from multiparous women. Around half of the preterm birth can be identified antenatally at high specificity using population-based routinely collected maternal and pregnancy data. The performance of the prediction models depends on the available predictor pool that is individual and time specific.

## Introduction

Preterm birth, defined as birth before 37 completed weeks of pregnancy, is a significant contributor to neonatal mortality and morbidity^1–4^, and infants born preterm are at increased risk of lifelong adverse neurological, intellectual, respiratory, metabolic and cardiovascular outcomes^5–9^. Approximately 15 million babies were born prematurely worldwide in 2014, and a large majority (81%) occurred in Asia and sub-Saharan Africa^10^. In Australia, the prevalence of preterm birth was 8.6% (2014), lower than the global and U.S. figures of 10.6% and 9.6%, respectively^10^. Nevertheless, it fared worse than other countries like Canada (8.2%) and the U.K. (7.0%) in the same period^10^. According to a report by the Australian Institute of Health and Welfare, the prevalence of preterm birth was 8.6% in 2019, and, despite advances in antenatal care, the occurrence was higher than the trough of 8.3% in 2010 and was barely lower than the peak of 8.7% in 2017–2018^11^. Importantly, Indigenous Australians were disproportionately affected, with a higher prevalence of 13.2%.

Given the profound health impact and the possibility of prevention when detected early^12,13^, prognostic models have been developed to identify pregnant women with increased risk of preterm birth using prenatal and perinatal predictors. However, findings from these studies were affected by a small sample size, a limited number of predictors, and methodological and reporting shortcomings^14–16^. The discrimination capacity, measured using the area under the receiver operating characteristic curve (AUC), of models developed to date, was found to be poor to moderate (58–77%)^16^. In a recent retrospective study that involved more than 2 million births in the U.S., the AUC of an early pregnancy cumulative risk scoring tool for preterm birth was 59.1% (95% confidence interval [CI] 58.9–59.4)^17^. Despite the large sample size, the study neither considered risk factors known to be strongly associated with preterm birth, such as socioeconomic status, nor reported additional prediction performance statistics for comparison purposes. At the other end of the spectrum, a French prospective study conducted in 2018 demonstrated that a nomogram (a graphical tool that shows the relationship between outcome probability and selected predictors) developed for personalised preterm birth risk evaluation has an AUC of 0.77 (95% CI 0.72–0.81). Nevertheless, this study was limited as only high-risk women were targeted, and some of the risk factors used in the prediction model were not routinely assessed (e.g., cervical length)^18^. Importantly, among the prognostic models developed, there is a paucity of evidence on preterm birth prediction that are timely and relevant to the Australian population and utilises readily available health information^19^.

Population-based research using routinely collected health data coupled with innovative approache for model generation can potentially improve prediction outcomes. Western Australia is the home to the largest and most comprehensive collection of routine perinatal data within the country, that span nearly four decades and has a complete geographic coverage^20^. Using data linkage, the predictive capability of the population-based database can be expanded by bringing in predictors from other health-related data sources^21^. Linkage can also be applied longitudinally to include historical events and vertically to capture features across generations^21^. Machine learning, a subset of artificial intelligence, has been used in reproductive medicine to automate pattern recognition^22^. By applying algorithms to learn and adapt from experience with data without being explicitly instructed, the technology has been adopted as an adjunct to clinicians in the U.S. for interpreting electronic foetal heart monitoring^23^. In predictive analytics, models developed using machine learning have been explored to tackle the challenge of identifying stillbirth and perinatal morbidities^24,25^. Recent advances in the field of machine learning enable the analysis of large-scale health data and improve prediction by generating models that may use novel and complex combinations of features (i.e., potential predictors) and weights (i.e., model parameters)^26^.

An effective prognostic model for preterm birth will contribute to the estimation of individual risk and aid in identifying potentially high-risk pregnancies. As a complement to standard antenatal care, the tool can inform surveillance strategies and enable efficient use of resources. Crucially, the resulting prediction is locally relevant and readily translatable to the Australian antenatal care program. The primary aim of this study was to develop models for predicting preterm birth in Western Australia using a suite of machine learning classification algorithms and a comprehensive range of routinely collected risk factors and internally validate these models. The secondary aim of this study was to compare the performance of the classification algorithms in predicting preterm birth.

## Methods

### Study design and population, and data sources

**Figure 1:**
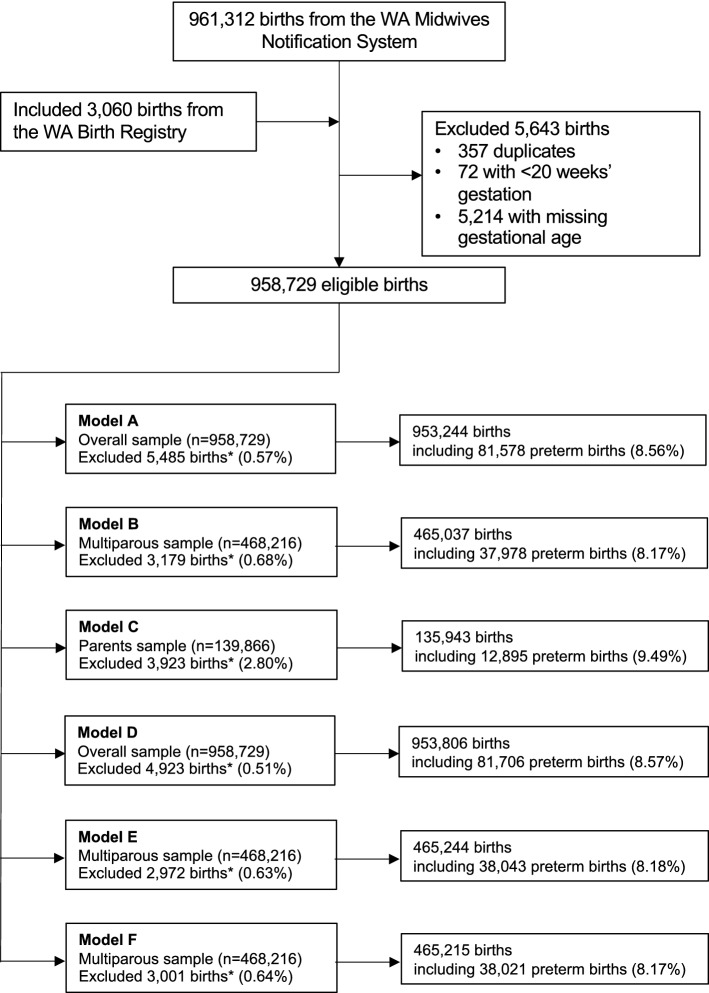
Study flow diagram *Births containing unknown values for a subset of predictors were excluded to reduce multicollinearity of predictors. These predictors were 1) Maternal chronic medical conditions: miscarriage (**A**, **B**, **C**, **D**, **E**, **F**); 2) Current pregnancy characteristics and complications: threatened preterm labour (**A**, **B**, **F**), parity (**A**, **B**, **D**), small-for-gestational age (**A**, **B**, **C**); 3) Past obstetric history: Caesarean section delivery (**B**, **E**, **F**), gestational age of the last pregnancy (**B**, **E**, **F**), small-for-gestational age (**B**, **E**, **F**); 4) Parent’s birth outcomes: preterm birth (**C**), small-for-gestational age (**C**); 5) Grandmothers’ past obstetric history: cancer registration (**C**).

Data for the longitudinal retrospective cohort study were sourced from core population-based administrative and health datasets, linked using the Western Australia (WA) Data Linkage System maintained by the WA Department of Health^27^. Birth records for the period from 1st January 1980 to 31st December 2015 were ascertained from the WA Midwives Notification System, a statutory data collection and encompasses all live births and stillbirths that occur from 20 weeks of gestation or have a birth weight of at least than 400 g^20^. These records, augmented with those from the WA Births Registrations, formed the preliminary study cohort. The final cohort was established by excluding duplicates as well as births with gestational age that was missing or less than 20 weeks (Fig. 1). We then used the provided record identifier to link the birth records to that of the WA Hospital Morbidity Data Collection, WA Cancer Registry, WA Death Registrations, WA Register of Developmental Anomalies—Birth Defects, and WA Family Connections, which allowed the identification of (1) maternal morbidities before and during pregnancy, (2) birth outcomes and (3) reported developmental anomalies for births and families. Extracted records for the cohort also included information on socio-demographic factors, chronic medical conditions, past and current pregnancy events, and infants’ characteristics.

### Outcome and predictors

Preterm birth was defined as births before 37 weeks of gestation. Both spontaneous and medically indicated preterm births were included in the definition. Predictors were categorised into six main groups, including (1) maternal socio-demographic factors, (2) maternal chronic medical conditions, (3) current pregnancy characteristics and complications, (4) past obstetric history, (5) parent’s birth outcomes, and (6) grandmothers’ chronic medical conditions and obstetric history. Maternal socio-demographic factors included age (< 20, 20–24, 25–29, 30–34, 35–39, ≥ 40 years), ethnicity (Caucasian, Indigenous, other), socio-economic status, remoteness of residence, and smoking. Based on the maternal residence at the time of birth, the Index of Relative Socioeconomic Disadvantage (IRSD) at the statistical local area level was used as a proxy for the individual-level socio-economic status^28^. The values were divided into quintiles for the state of WA, with the first category assigned as the most disadvantaged. The remoteness of residence was categorised as major cities or regional/remote using the Accessibility and Remoteness Index for Australia (ARIA +) score remoteness area categories^29^. Maternal chronic medical conditions included essential hypertension, diabetes mellitus, asthma, miscarriage, obesity and circulatory system diseases. Characteristics and complications in current and previous pregnancies included gestational diabetes, gestational hypertension, urinary tract infection, cancer registration (before or during pregnancy), pre-eclampsia, threatened miscarriage, placenta praevia, placental abruption, pre-labour rupture of membranes, unspecified antepartum haemorrhage, threatened preterm labour and uterine rupture. Other characteristics of current pregnancy included: parity (0, 1, 2, or ≥ 3), year of birth (in five-year periods), plurality (singleton, twin, multiple [> 2] gestation), presence of congenital anomalies, and small-for-gestational-age (SGA) birth as a proxy for foetal growth restriction. Congenital anomalies were defined as any birth defect registered in the WA Register of Developmental Anomalies which collects data, including diagnostic information for anomalies, on children with birth defects up until the age of six. We calculated SGA as the lowest 10th percentile of birthweight by sex and gestation within calendar intervals of five years. For past obstetric history, additional predictors included caesarean section delivery, stillbirth, gestational age of the last birth (< 28, 28–31, 32–36, ≥ 37 weeks), SGA birth and congenital anomalies. For women with two or more birth records during the study period, we coded the timing of occurrence for past obstetric events, indicating whether the condition/complication occurred in the last birth, not in the last but an earlier birth, no history, or unknown. For a subset of births to mothers and fathers who were born during the study period and whose birth details were included in our cohort, we ascertained whether they were born preterm, small for gestational age, or were diagnosed with congenital anomalies. For these parents, we also recorded the chronic medical conditions and past obstetric history of their mothers.

### Statistical methods

Descriptive statistics were used to summarise the characteristics of the study cohort and the prediction results. The minimal study size needed was ascertained using the method by Riley et al.^30^, which targets four aspects of a prediction model (namely margin of error in overall outcome risk, mean absolute prediction error, shrinkage of predictor effects and optimism in apparent model fit) to obtain a context-specific required sample size that minimises the potential for model overfitting while ensuring precise estimates of key parameters. Our sample size far exceeds the required numbers and is thus adequately designed to develop and validate the prognostic models.

We first developed three prediction models with full information on relevant predictor groups for specific target populations. For these models, we considered the inclusion of predictors collected after birth as a proxy for measurements taken during pregnancy (e.g., SGA). The first model (Model A) encompassed all births in the study population (n = 953,244, preterm births = 81,578) (Fig. 1) and employed predictors related to maternal socio-demographic factors, maternal chronic medical conditions, and current pregnancy characteristics and complications. The second model (Model B) extended Model A by adding predictors related to past obstetric history, and the cohort was limited to births of multiparous women (n = 465,037, preterm births = 37,978). The third model (Model C) comprised predictors of Model A plus those of parent’s birth outcomes and grandmothers’ chronic medical conditions and obstetric history, and the cohort was restricted to births of parents who were born during the study period as previously described (n = 135,943, preterm births = 12,895).

We then developed three additional models with limited sets of predictors to emulate the temporal availability of data at a specific time of contact during the pregnancy period. The first model (Model D) contained predictors that were collected at the early phase of pregnancy (e.g., at initial antenatal appointment) for both nulliparous and multiparous women (n = 953,806, preterm births = 81,706) (Fig. 1). The second model (Model E) extended Model D by adding past obstetric history predictors, and thus the cohort was limited to births of multiparous women (n = 465,244, preterm births = 38,043). The third model (Model F) included predictors available in the late term of pregnancy. It was a replica of Model B with the exclusion of SGA and congenital anomalies, as these conditions were ascertained after birth (n = 465,215, preterm births = 38,021).

In all prediction models, the occurrence of preterm births was far outweighed by the number of term births. The unequal distribution of classes in the dataset created an imbalanced classification problem that required special attention during model development (e.g., class weighting) and evaluation (e.g., reporting using true positive rate and positive predictive value instead of accuracy).

### Classification algorithms

We applied five different supervised machine learning algorithms to solve the binary classification problem of identifying preterm and term births: (1) regularised logistic regression; (2) decision trees via classification and regression trees (CART); (3) Random Forests; (4) extreme gradient boosting; and (5) multi-layer perceptron. These classification algorithms, or classifiers, were chosen because of their widespread application, relative ease of use, and diverse learning capabilities. They also represent a spectrum of classifiers ranging from conventional (e.g., logistic regression) to modern (e.g., extreme gradient boosting).

Regularised logistic regression (LR) is an extension of logistic regression that incorporates a penalty term on the cost function. The L1 penalisation used resulted in variable selection by shrinking the regression coefficient of predictors that contribute least to the prediction and thus addressing the issue of overfitting.

Decision trees (DT) use the CART algorithm to make predictions by selecting the most differentiating predictors using an impurity criterion (Gini impurity or information gain using entropy) at the root and successive nodes to split the sample into the best homogenous sets of sub-samples. The risk of overfitting was minimised by pruning the depth of the tree and restricting the number of predictors to consider when looking for the best split.

Random Forests (RF) employs bootstrap aggregating (or bagging) to build an ensemble of decision trees using random sample subsets and then combine the results by averaging the probabilistic prediction of the trees. This classification approach reduces variance by combining diverse trees thus further lessons the effect of overfitting and improving prediction performance as well.

Gradient boosting is a method of converting weak learners (e.g., decision trees) into strong ones by first identifying shortcomings of a weak learner through gradients (or first-order derivatives) of the loss function and then improving upon, or boosting, predictions of the weak learner by adding more weak ones in a gradual, additive, and sequential manner. Extreme gradient boosting (XGBoost [XGB]) is a specific implementation of the gradient boosting method which enhances efficiency through parallelisation and delivers more accurate estimation by using the second-order derivative of the loss function and L1 and L2 regularisations.

Multi-layer perceptron (MLP) is an artificial neural network that mimics the learning processes found in the neural network of the human brain. The classifier processes information starting from an input layer, through one or more hidden layers, and ending at an output layer. Learning was facilitated through backpropagation using stochastic gradient descent. Weights were then learnt through backpropagation, and the activation functions were selected as hyperparameters. The nodes of the input layer represent the predictors in the model, and the nodes of the remaining layers represent the non-linear functions of weighted combinations of nodes in the previous layers.

### Model development

All predictors were categorised as previously described and checked for missing values. For each model, records with unknown values for a subset of predictors were excluded to reduce multicollinearity. Predictors were pre-processed using either one-hot encoding (excluding binary variables) for DT, RF, XGB and MLP, or dummy encoding for LR. Class weight was used in all classifiers except MLP (class weight is not supported in scikit-learn implementation of MLP) to handle imbalanced outcomes. Hyperparameter tuning was carried out using a grid search (LR, DT), or Bayesian optimisation (using the Tree-Parzen Estimator algorithm) combined with the asynchronous HyperBand scheduler (RF, XGB, MLP) over specified parameter search space. The hyperparameters of the classifiers were optimised for F_1_ score, the harmonic mean of positive predictive value and sensitivity, by tenfold cross-validation. Model performance and generalisability were evaluated using internal validation, which was undertaken using cross-validation by first randomly partitioning the data into 10 equal portions with the same prevalence of the outcome in each fold via the stratified k-fold cross-validation technique. The model was then trained using data from the first 9 folds and validated on the 10th fold, and the process was repeated iteratively, resulting in 10 model variants for each classifier. We measured the overall performance of the classifier by averaging the values of each selected evaluation metric.

### Model evaluation

The performance of each classifier was based on the ability to discriminate between preterm births and term births and was evaluated using the following classification metrics: 1) area under the receiver operating characteristic curve (AUC); 2) accuracy (ACC); 3) F_1_ score (F1); 4) true positive rate (TPR) (also called sensitivity or recall); 5) positive predictive value (PPV) (also called precision); 6) negative predictive value (NPV); 7) positive likelihood ratio (LR +); and 8) negative likelihood ratio (LR-). Accuracy was defined as the proportion of correctly classified individuals. For comparison with prior studies, performance results were reported at a false positive rate (FPR) of 5% (95% specificity) and 10% (90% specificity). As the outcome classes are highly imbalanced (i.e., the number of term births is overwhelmingly higher than that of preterm births), accuracy would be erroneously high even if the classifier predicts all term births correctly and misclassifies all preterm births. Therefore, AUC and F1 were mainly used in our reporting of results. The latter was chosen because of its balanced view of TPR and PPV, which we assumed are equally important in preterm birth prediction. Feature importance, a non-directional measure of the individual contribution of the corresponding predictor towards model performance for a specific classifier, was reported. Scoring was determined using a non-standardised absolute beta coefficient for LR and impurity-based importance measure for tree-based classifiers, and ranking was performed in descending order of the value. The top 10 ranked one-hot or dummy encoded predictors were then compared. To enable interpretation of the models generated by non-linear tree-based classifiers, the impact of each encoded predictor on the prediction of preterm birth in the models developed by the best classifier was estimated using SHapley Additive exPlanations (SHAP), a unified framework that extends the classic Shapley values from game theory to explain the output of machine learning model^31^.

All analyses were conducted using miniconda3 4.10.1–5, python 3.8.9, pandas 1.3.0, numpy 1.19.5, scikit-learn 0.23.2, tune-sklearn 0.4.0 and shap 0.41.0. The results were reported following the Transparent Reporting of a multivariable prediction model for Individual Prognosis or Diagnosis (TRIPOD) statement^32^.

### Ethics

The study was approved by the Human Research Ethics Committees of the Department of Health WA and Curtin University. All research activities were conducted in accordance with corresponding guidelines and regulations. Informed consent was waived by the Department of Health WA Human Research Ethics Committee (PRN RGS0000005148) because the study uses existing and consented registry data and presents minimal harm to participants while offering potential benefits to the public.

## Results

### Study population

**Figure 2:**
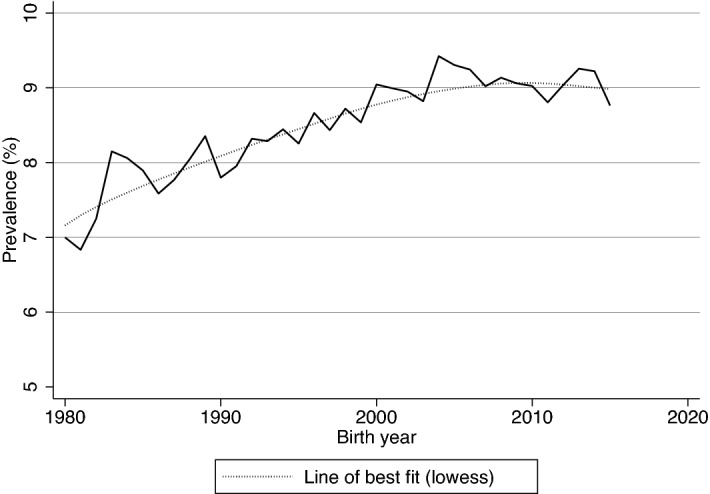
Annual prevalence of preterm birth in Western Australia for the period 1980–2015. Lowess: the local linear smooth plot was generated using Cleveland’s tricube weighting function with a bandwidth of 0.8 (Cleveland, W. S. 1979. Robust locally weighted regression and smoothing scatterplots. Journal of the American Statistical Association 74: 829–836).

The characteristics of the 958,729 births, including 81,974 (8.6%) preterm births and 876,755 (91.4%) term births, are shown in Supplementary Table 1. When viewed by birth decade, the proportion of births of women aged at or after 35 years tripled from 6.7% in the 1980s to 21.1% in the 1st half of the 2010s (Supplementary Table 2). Likewise, the portion of births of women of ethnicity other than Caucasian and Indigenous has increased over time (1980s: 5.1%; first half of 2010s 25.0%). There was a gradual improvement in socio-economic status, as indicated by the top two IRSD quintiles, from close to a third (30.7%) in the 1980s to nearly half (47.0%) in the most recent decade studied. A similar trend was observed for the remoteness of residence where the majority lived in major cities and had been increasing since the 1980s (1980s 61.8%; first half of 2010s 72.7%). Among the maternal chronic medical risk factors, the prevalence of essential hypertension has increased seven folds from 0.08% in the 1980s to 0.62% in the most recent decade studied. A similar rise was noted for diabetes mellitus and obesity. On the other hand, circulatory heart disease has become less common over time, with 0.6–1.9% of cases observed in the 1980s and 1990s and only 0.2% in the 2010s. Regarding pregnancy characteristics, nearly two-fifth (38.9%) of the births were nulliparous in the 1980s and became more so over the decades (first half of 2010s 42.5%). Notably, the prevalence of most of the pregnancy complications observed appeared to improve over time. The prevalence of preterm birth has plateaued from the peak of 9.1% in the 2000s to 9.0% in the period 2010–2015 after decades of increase (Supplementary Table 2, Fig. 2).

### Model performance

Predictors used in the prediction models and the tuned hyperparameters optimised for F1 by models are shown in Supplementary Tables 3 and 4, respectively. Overall, at 5% FPR using tenfold cross-validation, the AUCs ranged from 57.70% (Model D, DT; 95% confidence interval [CI] 57.54–57.86) to 86.43% (Model B, MLP; 95% CI 86.21–86.65) and the F1s varied from 12.89% (Model D, LR; 95% CI 12.59–13.19) to 50.44% (Model B, MLP; 95% CI 49.99–50.89) (Table 1), suggesting that prediction performance depended on the selection of the predictors and classifiers. Across the six models, ensemble and neural network classifiers (i.e., XGB and MLP) shared the top spots in prediction performance. The performance of classifiers by models at 10% FPR using tenfold cross-validation is shown in Table 2.

**Table 1:** Performance of models for predicting preterm birth at 5% FPR using tenfold cross-validation, by classification algorithms.

Model	Algorithm	Evaluation metrics							
		AUC, %	Accuracy, %	F1, %	TPR, %	PPV, %	NPV, %	LR +	LR-
		Mean (95% CI)	Mean (95% CI)	Mean (95% CI)	Mean (95% CI)	Mean (95% CI)	Mean (95% CI)	Mean (95% CI)	Mean (95% CI)
A	LR	83.56 (83.35,83.77)	90.80 (90.77,90.83)	46.08 (45.80,46.36)	45.93 (45.57,46.29)	46.23 (46.04,46.43)	94.94 (94.91,94.97)	9.19 (9.12,9.26)	0.57 (0.57,0.57)
	DT	82.72 (82.52,82.92)	90.98 (90.96,91.01)	47.55 (47.28,47.82)	47.76 (47.34,48.17)	47.35 (47.18,47.51)	95.11 (95.07,95.14)	9.61 (9.55,9.67)	0.55 (0.55,0.55)
	RF	84.23 (84.04,84.42)	90.99 (90.96,91.02)	47.75 (47.47,48.03)	48.13 (47.76,48.50)	47.39 (47.19,47.58)	95.14 (95.11,95.17)	9.62 (9.55,9.70)	0.55 (0.54,0.55)
	XGB	84.34 (84.14,84.54)	91.05 (91.02,91.09)	48.33 (48.02,48.64)	48.89 (48.48,49.30)	47.78 (47.57,47.99)	95.21 (95.17,95.24)	9.78 (9.69,9.86)	0.54 (0.53,0.54)
	MLP	84.41 (84.21,84.61)	91.08 (91.05,91.11)	48.55 (48.31,48.79)	49.19 (48.87,49.51)	47.94 (47.77,48.10)	95.23 (95.20,95.26)	9.84 (9.77,9.90)	0.53 (0.53,0.54)
B	LR	85.88 (85.71,86.05)	91.38 (91.31,91.44)	48.95 (48.32,49.57)	50.63 (49.77,51.49)	47.37 (46.96,47.79)	95.58 (95.51,95.66)	10.13 (9.96,10.29)	0.52 (0.51,0.53)
	DT	83.27 (83.12,83.42)	91.30 (91.22,91.38)	48.25 (47.77,48.73)	49.66 (48.94,50.38)	46.93 (46.47,47.39)	95.50 (95.44,95.56)	9.95 (9.76,10.13)	0.53 (0.52,0.54)
	RF	85.47 (85.28,85.66)	91.37 (91.31,91.42)	48.83 (48.34,49.33)	50.46 (49.78,51.14)	47.31 (46.97,47.65)	95.57 (95.51,95.63)	10.10 (9.96,10.24)	0.52 (0.51,0.53)
	XGB	86.22 (86.05,86.39)	91.43 (91.36,91.51)	49.46 (48.83,50.09)	51.32 (50.46,52.19)	47.72 (47.30,48.15)	95.64 (95.57,95.72)	10.27 (10.09,10.44)	0.51 (0.50,0.52)
	MLP	86.43 (86.21,86.65)	91.54 (91.49,91.60)	50.44 (49.99,50.89)	52.69 (52.06,53.31)	48.37 (48.07,48.67)	95.76 (95.71,95.81)	10.54 (10.41,10.67)	0.50 (0.49,0.50)
C	LR	83.67 (83.03,84.31)	90.29 (90.18,90.41)	47.02 (46.10,47.94)	45.43 (44.28,46.58)	48.73 (48.07,49.38)	94.32 (94.21,94.44)	9.08 (8.84,9.32)	0.57 (0.56,0.59)
	DT	81.34 (80.81,81.87)	90.28 (90.13,90.42)	46.63 (45.81,47.45)	44.80 (43.69,45.91)	48.66 (47.84,49.47)	94.26 (94.16,94.37)	9.05 (8.76,9.35)	0.58 (0.57,0.59)
	RF	83.10 (82.54,83.66)	90.35 (90.24,90.46)	47.42 (46.49,48.35)	45.91 (44.72,47.10)	49.04 (48.41,49.68)	94.37 (94.25,94.49)	9.19 (8.96,9.42)	0.57 (0.56,0.58)
	XGB	84.09 (83.47,84.71)	90.50 (90.37,90.62)	48.67 (47.70,49.64)	47.54 (46.30,48.78)	49.87 (49.20,50.54)	94.53 (94.41,94.65)	9.50 (9.25,9.76)	0.55 (0.54,0.57)
	MLP	83.23 (82.66,83.80)	90.34 (90.22,90.47)	47.41 (46.39,48.42)	45.91 (44.62,47.20)	49.02 (48.31,49.72)	94.37 (94.24,94.50)	9.18 (8.93,9.44)	0.57 (0.56,0.58)
D	LR	58.20 (58.04,58.36)	87.77 (87.74,87.79)	12.89 (12.59,13.19)	10.56 (10.30,10.83)	16.52 (16.17,16.87)	91.89 (91.87,91.92)	2.11 (2.06,2.17)	0.94 (0.94,0.94)
	DT	57.70 (57.54,57.86)	87.72 (87.64,87.81)	12.92 (12.69,13.14)	10.63 (10.38,10.88)	16.46 (16.24,16.69)	91.90 (91.88,91.91)	2.10 (2.07,2.14)	0.94 (0.94,0.94)
	RF	58.42 (58.24,58.60)	87.78 (87.77,87.80)	13.10 (12.90,13.30)	10.75 (10.57,10.93)	16.77 (16.53,17.00)	91.91 (91.90,91.93)	2.15 (2.11,2.19)	0.94 (0.94,0.94)
	XGB	58.70 (58.52,58.88)	87.80 (87.79,87.82)	13.38 (13.18,13.59)	11.00 (10.82,11.18)	17.09 (16.86,17.32)	91.93 (91.92,91.95)	2.20 (2.16,2.24)	0.94 (0.93,0.94)
	MLP	58.69 (58.54,58.84)	87.80 (87.78,87.82)	13.29 (13.08,13.51)	10.92 (10.73,11.11)	16.99 (16.74,17.24)	91.92 (91.91,91.94)	2.19 (2.15,2.22)	0.94 (0.94,0.94)
E	LR	69.01 (68.67,69.35)	89.11 (89.07,89.14)	25.57 (25.21,25.93)	22.89 (22.53,23.26)	28.97 (28.63,29.30)	93.26 (93.23,93.29)	4.58 (4.51,4.65)	0.81 (0.81,0.82)
	DT	66.86 (66.60,67.12)	88.57 (88.23,88.91)	25.94 (25.12,26.77)	24.58 (22.97,26.19)	27.76 (27.05,28.47)	93.35 (93.25,93.45)	4.32 (4.17,4.47)	0.80 (0.79,0.81)
	RF	67.70 (67.44,67.96)	89.09 (89.05,89.14)	25.46 (24.93,25.98)	22.78 (22.24,23.32)	28.85 (28.37,29.33)	93.25 (93.21,93.29)	4.56 (4.45,4.66)	0.81 (0.81,0.82)
	XGB	69.26 (68.91,69.61)	89.13 (89.09,89.17)	25.90 (25.46,26.34)	23.23 (22.78,23.69)	29.26 (28.85,29.67)	93.29 (93.25,93.32)	4.65 (4.55,4.74)	0.81 (0.80,0.81)
	MLP	69.11 (68.81,69.41)	89.14 (89.12,89.17)	26.04 (25.72,26.36)	23.38 (23.04,23.71)	29.39 (29.10,29.69)	93.30 (93.27,93.33)	4.68 (4.61,4.74)	0.81 (0.80,0.81)
F	LR	85.53 (85.22,85.84)	91.34 (91.29,91.39)	48.68 (48.23,49.14)	50.25 (49.62,50.88)	47.21 (46.91,47.52)	95.55 (95.49,95.60)	10.05 (9.93,10.18)	0.52 (0.52,0.53)
	DT	83.87 (83.50,84.24)	91.20 (91.07,91.34)	48.16 (47.58,48.74)	50.03 (48.74,51.32)	46.50 (45.79,47.21)	95.52 (95.42,95.62)	9.78 (9.49,10.06)	0.53 (0.51,0.54)
	RF	85.35 (85.03,85.67)	91.32 (91.27,91.37)	48.50 (48.08,48.92)	50.00 (49.43,50.58)	47.08 (46.80,47.37)	95.53 (95.48,95.58)	10.00 (9.88,10.11)	0.53 (0.52,0.53)
	XGB	85.62 (85.31,85.93)	91.40 (91.34,91.45)	49.15 (48.63,49.68)	50.90 (50.17,51.62)	47.53 (47.19,47.88)	95.60 (95.54,95.66)	10.18 (10.04,10.32)	0.52 (0.51,0.52)
	MLP	85.75 (85.47,86.03)	91.47 (91.42,91.52)	49.80 (49.34,50.26)	51.78 (51.14,52.42)	47.97 (47.66,48.27)	95.68 (95.62,95.73)	10.36 (10.23,10.49)	0.51 (0.50,0.51)

AUC, area under the receiving-operator characteristic curve; TPR, true positive rate (recall or sensitivity); PPV, positive predictive value (precision); NPV, negative predictive value; LR + , positive likelihood ratio; LR − , negative likelihood ratio; CI, confidence interval; FPR, false positive rate (1-specificity); LR, regularised logistic regression; DT, decision trees; RF, Random Forests; XGB, extreme gradient boosting; MLP, multi-layer perceptron.

Model A: Cohort—all births; Predictors—maternal socio-demographic factors, maternal chronic medical conditions, and current pregnancy characteristics and complications; Model B: Cohort—births of multiparous women; Predictors—Model A + maternal past obstetric history; Model C: Cohort—births of parents who were born during the study period; Predictors—Model A + parent’s birth outcomes and grandmother’s chronic medical conditions and obstetric history; Model D: Cohort—all births; Predictors—maternal socio-demographic factors, maternal chronic medical conditions, parity, and birth year; Model E: Cohort—births of multiparous women; Predictors—Model D + maternal past obstetric history; Model F: Cohort—births of multiparous women; Predictors—Model B excluding small-for-gestational age and congenital anomalies in current birth.

**Table 2:** Performance of models for predicting preterm birth at 10% FPR using tenfold cross-validation, by classification algorithms.

Model	Algorithm	Evaluation metrics							
		AUC, %	Accuracy, %	F1, %	TPR, %	PPV, %	NPV, %	LR +	LR-
		Mean (95% CI)	Mean (95% CI)	Mean (95% CI)	Mean (95% CI)	Mean (95% CI)	Mean (95% CI)	Mean (95% CI)	Mean (95% CI)
A	LR	83.56 (83.35,83.77)	87.43 (87.41,87.46)	44.97 (44.81,45.14)	60.01 (59.72,60.29)	35.96 (35.85,36.07)	96.01 (95.98,96.03)	6.00 (5.97,6.03)	0.44 (0.44,0.45)
	DT	82.79 (82.62,82.96)	87.49 (87.44,87.55)	45.63 (45.48,45.78)	61.32 (61.13,61.51)	36.33 (36.19,36.48)	96.13 (96.11,96.15)	6.10 (6.06,6.14)	0.43 (0.43,0.43)
	RF	84.24 (84.05,84.43)	87.57 (87.54,87.60)	45.90 (45.72,46.08)	61.62 (61.30,61.93)	36.57 (36.45,36.69)	96.16 (96.13,96.19)	6.16 (6.13,6.19)	0.43 (0.42,0.43)
	XGB	84.34 (84.15,84.53)	87.62 (87.59,87.65)	46.23 (46.05,46.41)	62.19 (61.89,62.50)	36.79 (36.67,36.91)	96.22 (96.19,96.25)	6.22 (6.19,6.25)	0.42 (0.42,0.42)
	MLP	84.40 (84.21,84.59)	87.64 (87.61,87.67)	46.35 (46.14,46.57)	62.41 (62.04,62.78)	36.87 (36.73,37.01)	96.24 (96.20,96.27)	6.24 (6.20,6.28)	0.42 (0.41,0.42)
B	LR	85.88 (85.71,86.05)	87.90 (87.85,87.94)	46.44 (46.13,46.74)	64.25 (63.70,64.80)	36.36 (36.16,36.55)	96.59 (96.54,96.64)	6.42 (6.37,6.48)	0.40 (0.39,0.40)
	DT	83.42 (83.22,83.62)	87.87 (87.79,87.96)	46.28 (45.98,46.57)	63.96 (63.39,64.54)	36.25 (36.02,36.49)	96.56 (96.51,96.61)	6.40 (6.33,6.46)	0.40 (0.39,0.41)
	RF	85.44 (85.27,85.61)	87.95 (87.90,88.00)	46.79 (46.46,47.12)	64.89 (64.29,65.48)	36.59 (36.37,36.80)	96.65 (96.59,96.70)	6.49 (6.43,6.55)	0.39 (0.38,0.40)
	XGB	86.22 (86.04,86.40)	88.00 (87.95,88.05)	47.14 (46.80,47.49)	65.53 (64.89,66.16)	36.81 (36.59,37.04)	96.71 (96.65,96.76)	6.55 (6.49,6.61)	0.38 (0.38,0.39)
	MLP	86.41 (86.20,86.62)	88.06 (88.01,88.11)	47.53 (47.22,47.84)	66.24 (65.68,66.79)	37.06 (36.86,37.27)	96.77 (96.72,96.82)	6.62 (6.56,6.68)	0.38 (0.37,0.38)
C	LR	83.67 (83.03,84.31)	87.14 (87.02,87.26)	46.86 (46.12,47.60)	59.79 (58.57,61.01)	38.53 (38.04,39.02)	95.53 (95.40,95.66)	5.98 (5.86,6.11)	0.45 (0.43,0.46)
	DT	81.36 (80.64,82.08)	87.61 (87.35,87.87)	46.44 (45.55,47.34)	56.61 (55.63,57.60)	39.38 (38.51,40.24)	95.23 (95.13,95.34)	6.21 (5.98,6.43)	0.48 (0.47,0.49)
	RF	83.13 (82.57,83.69)	87.16 (87.03,87.28)	46.97 (46.20,47.75)	60.00 (58.71,61.29)	38.60 (38.09,39.11)	95.55 (95.41,95.69)	6.00 (5.87,6.13)	0.44 (0.43,0.46)
	XGB	84.11 (83.49,84.73)	87.29 (87.17,87.41)	47.76 (47.00,48.52)	61.28 (59.99,62.57)	39.13 (38.63,39.63)	95.69 (95.55,95.82)	6.14 (6.01,6.27)	0.43 (0.42,0.44)
	MLP	83.01 (82.41,83.61)	87.11 (86.99,87.23)	46.67 (45.90,47.44)	59.49 (58.21,60.76)	38.40 (37.89,38.91)	95.50 (95.36,95.63)	5.95 (5.82,6.08)	0.45 (0.44,0.46)
D	LR	58.20 (58.04,58.36)	83.83 (83.80,83.85)	15.98 (15.78,16.18)	17.95 (17.71,18.20)	14.40 (14.23,14.57)	92.13 (92.11,92.15)	1.80 (1.77,1.82)	0.91 (0.91,0.91)
	DT	57.76 (57.59,57.93)	83.90 (83.85,83.96)	16.16 (16.05,16.28)	18.12 (17.93,18.30)	14.59 (14.52,14.67)	92.15 (92.14,92.16)	1.82 (1.81,1.83)	0.91 (0.91,0.91)
	RF	58.43 (58.27,58.59)	83.87 (83.85,83.89)	16.37 (16.18,16.56)	18.42 (18.19,18.66)	14.72 (14.57,14.88)	92.17 (92.15,92.19)	1.84 (1.82,1.87)	0.91 (0.90,0.91)
	XGB	58.70 (58.52,58.88)	83.89 (83.86,83.91)	16.58 (16.37,16.79)	18.69 (18.43,18.95)	14.89 (14.72,15.07)	92.20 (92.17,92.22)	1.87 (1.84,1.89)	0.90 (0.90,0.91)
	MLP	58.69 (58.53,58.85)	83.88 (83.86,83.91)	16.59 (16.41,16.78)	18.71 (18.49,18.94)	14.90 (14.75,15.06)	92.20 (92.18,92.22)	1.87 (1.85,1.89)	0.90 (0.90,0.91)
E	LR	69.01 (68.66,69.36)	85.47 (85.43,85.51)	28.04 (27.70,28.39)	34.63 (34.14,35.12)	23.56 (23.31,23.82)	93.92 (93.88,93.97)	3.46 (3.41,3.51)	0.73 (0.72,0.73)
	DT	66.96 (66.64,67.28)	85.53 (85.46,85.61)	27.23 (26.84,27.62)	33.11 (32.43,33.80)	23.13 (22.87,23.38)	93.81 (93.75,93.86)	3.38 (3.33,3.43)	0.74 (0.73,0.75)
	RF	67.71 (67.45,67.97)	85.39 (85.36,85.43)	27.36 (27.06,27.65)	33.64 (33.21,34.06)	23.05 (22.83,23.27)	93.84 (93.80,93.88)	3.36 (3.32,3.41)	0.74 (0.73,0.74)
	XGB	69.24 (68.91,69.57)	85.50 (85.46,85.55)	28.35 (27.98,28.71)	35.07 (34.54,35.60)	23.79 (23.52,24.06)	93.96 (93.92,94.01)	3.51 (3.45,3.56)	0.72 (0.72,0.73)
	MLP	69.14 (68.80,69.48)	85.50 (85.45,85.54)	28.24 (27.86,28.61)	34.89 (34.35,35.43)	23.71 (23.44,23.99)	93.95 (93.90,94.00)	3.49 (3.44,3.54)	0.72 (0.72,0.73)
F	LR	85.53 (85.22,85.84)	87.85 (87.79,87.91)	46.14 (45.75,46.54)	63.69 (62.97,64.41)	36.18 (35.92,36.43)	96.53 (96.47,96.60)	6.37 (6.30,6.44)	0.40 (0.40,0.41)
	DT	83.75 (83.42,84.08)	87.75 (87.65,87.85)	45.84 (45.46,46.23)	63.43 (62.73,64.13)	35.89 (35.59,36.19)	96.51 (96.44,96.57)	6.29 (6.21,6.38)	0.41 (0.40,0.41)
	RF	85.35 (85.04,85.66)	87.91 (87.85,87.97)	46.54 (46.14,46.94)	64.42 (63.69,65.16)	36.43 (36.17,36.69)	96.60 (96.53,96.67)	6.44 (6.37,6.51)	0.40 (0.39,0.40)
	XGB	85.63 (85.32,85.94)	87.89 (87.83,87.95)	46.41 (46.03,46.80)	64.19 (63.50,64.87)	36.35 (36.10,36.60)	96.58 (96.52,96.64)	6.42 (6.35,6.49)	0.40 (0.39,0.41)
	MLP	85.68 (85.32,86.04)	87.91 (87.87,87.96)	46.57 (46.23,46.91)	64.45 (63.83,65.07)	36.46 (36.24,36.67)	96.60 (96.55,96.66)	6.45 (6.39,6.51)	0.40 (0.39,0.40)

AUC, area under the receiving-operator characteristic curve; TPR, true positive rate (recall or sensitivity); PPV, positive predictive value (precision); NPV, negative predictive value; LR + , positive likelihood ratio; LR − , negative likelihood ratio; CI, confidence interval; FPR, false positive rate (1-specificity); LR, regularised logistic regression; DT, decision trees; RF, Random Forests; XGB, extreme gradient boosting; MLP, multi-layer perceptron.

Model A: Cohort—all births; Predictors—maternal socio-demographic factors, maternal chronic medical conditions, and current pregnancy characteristics and complications; Model B: Cohort—births of multiparous women; Predictors—Model A + maternal past obstetric history; Model C: Cohort—births of parents who were born during the study period; Predictors—Model A + parent’s birth outcomes and grandmother’s chronic medical conditions and obstetric history; Model D: Cohort—all births; Predictors—maternal socio-demographic factors, maternal chronic medical conditions, parity, and birth year; Model E: Cohort—births of multiparous women; Predictors—Model D + maternal past obstetric history; Model F: Cohort—births of multiparous women; Predictors—Model B excluding small-for-gestational age and congenital anomalies in current birth.

**Figure 3:**
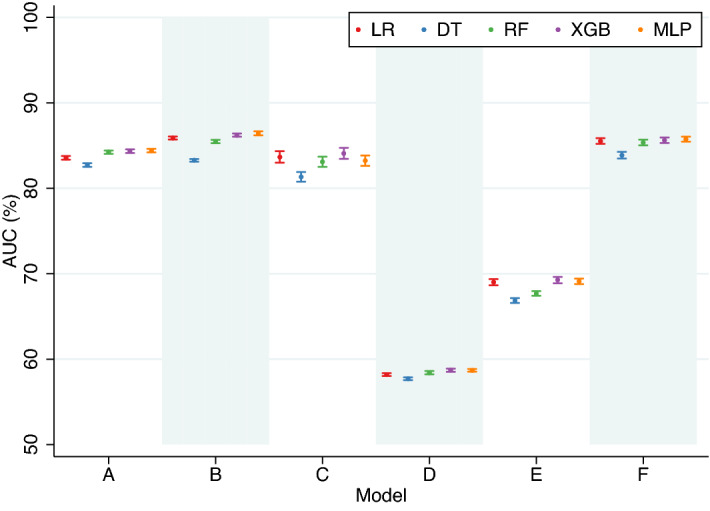
Performance, evaluated using AUC, of classification algorithms (10-fold CV), by models. AUC, area under the receiver operating characteristic curve; LR, regularised logistic regression; DT, decision trees; RF, Random Forest; XGB, extreme gradient boosting; MLP, multi-layer perceptron; CV, cross-validation.

**Figure 4:**
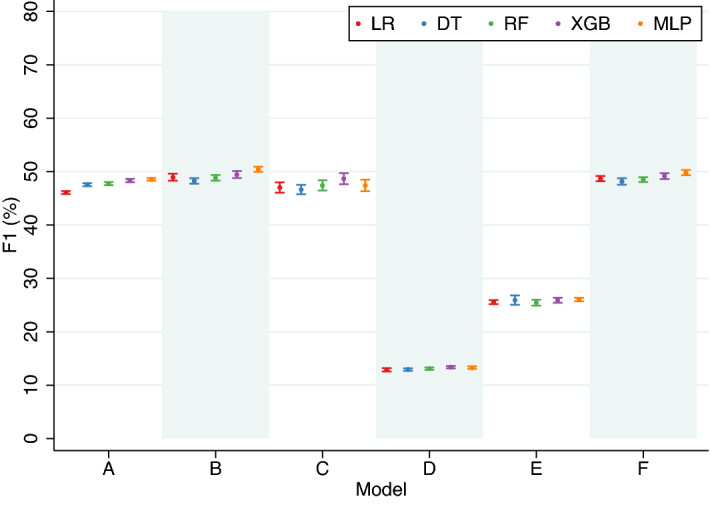
Performance, evaluated using F1, of classification algorithms (10-fold CV), by models. F1, F1 score; LR, regularised logistic regression; DT, decision trees; RF, Random Forest; XGB, extreme gradient boosting; MLP, multi-layer perceptron; CV, cross-validation.

For model A, which included maternal socio-demographic factors, chronic medical conditions and current pregnancy characteristics and complications-related predictors, the best performing classifier (MLP) achieved an AUC of 84.41% (95% CI 84.21–84.61) and a F1 of 48.55% (95% CI 48.31–48.79) at 5% FPR (Table 1, Figs. 3 and 4), a 2.0% and 5.4% increase in the respective metrics over the lowest-performing classifiers (DT and LR). For model B, adding predictors of past obstetric history to model A improved performance, as measured using AUC and F1, by 1–2 percentage points. MLP was the best-performing classifier with an AUC of 86.43% (95% CI 86.21–86.65) and a F1 of 50.44% (95% CI 49.99–50.89). The margin of improvement between the lowest (DT) and highest-performing classifiers was 3.8% and 4.5% for AUC and F1, respectively. For model C, including family history only marginally improved the prediction performances compared to that of Model A. The best-performing classifier (XGB) outperformed the lowest one (DT) by a similar magnitude as that in model B (AUC: 3.4%, F1: 4.4%).

Model D included predictors that were available in the early period of pregnancy. The prediction performances were the lowest among the models, with the best-performing classifier (XGB) achieving an AUC of 58.70% (95% CI 58.52–58.88) and a F1 of 13.38% (95% CI 13.18–13.59) at 5% FPR (Table 1, Figs. 3 and 4). Model E added predictors of past obstetric history to Model D and the performance, based on the best performers, increased moderately with an AUC of 69.26% (95% CI 68.91–69.61; XGB) and a F1 of 26.04% (95% CI 25.72–26.36; MLP). In Model F, factors ascertained after birth (i.e., SGA and congenital anomalies) were excluded from Model B, and this has a negligible impact on the performance of preterm birth prediction, as illustrated by the fact that the AUC (85.75%, 95% CI 85.47–86.03) and F1 (49.80%, 95% CI 49.34–50.26) of the best performing classifier (MLP) were very similar to that in Model B.

### Feature importance

Feature importance of the six models by classifiers are presented in Supplementary Tables 5 and 6. The estimated coefficients of the models produced using regularised logistic regression algorithm are reported in Supplementary Tables 7 and 8. For Model A, current pregnancy characteristics and complications dominated the top 10 features of importance (Supplementary Table 5). In particular, conditions such as pre-labour rupture of membranes, singleton birth and threatened miscarriage were high on the list among the tree-based classifiers, whilst, for LR, multiple gestations and threatened preterm labour shared the top 3 spots. The inclusion of past obstetric history in Model B slightly changed the rankings, with previous gestational age being considered important for DT, RF and XGB but less so for LR. For Model C, none of the predictors related to family history appeared on the ten highest-ranked features. For Model D, most maternal socio-demographic factors, chronic medical conditions, and parity were featured on the list (Supplementary Table 6). Specifically, diabetes and ethnicity were highly ranked, and maternal age was considered important for LR but not so much for the tree-based classifiers. Predictors of past obstetric history, particularly previous gestational age and caesarean delivery in the last birth, became the dominant contributors to the model performance of Model E after they were added to the early pregnancy predictors in Model D. For Model F, the ranking was similar to that of Model B after exclusion of SGA and congenital anomalies.

### Model interpretation

**Figure 5:**
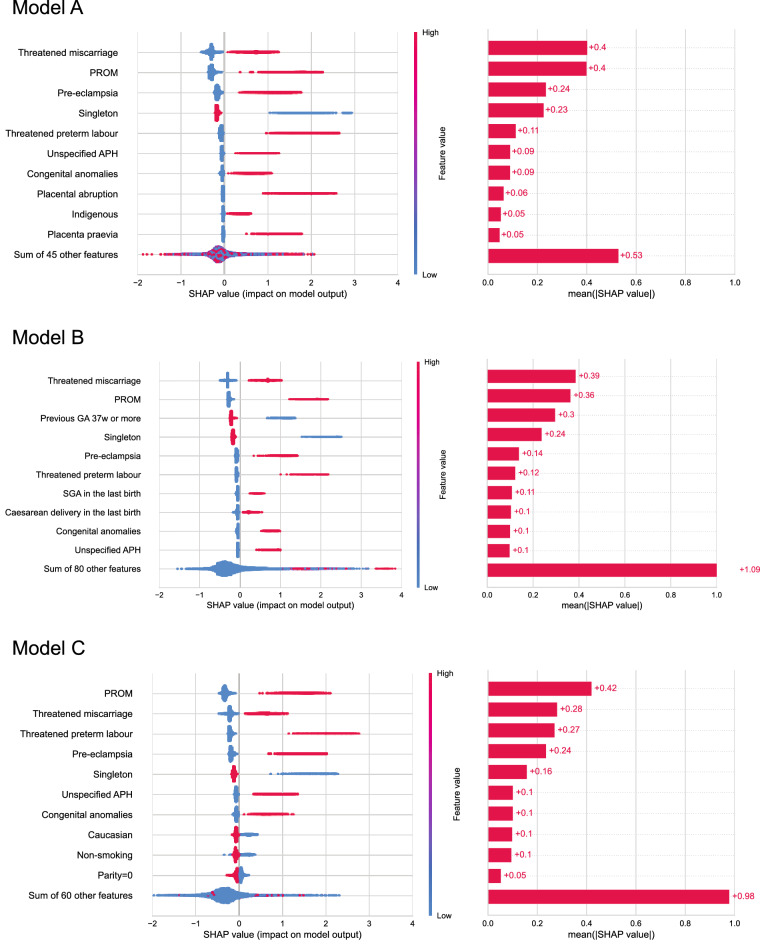
Summary plot of the SHapley Additive exPlanations (SHAP) value for models (**A**), (**B**) and (**C**) developed using the XGBoost classifier. The “beeswarm” plot on the left shows the distribution of the SHAP value (in log-odds scale) calculated using the test dataset. Each dot represents a prediction result of a particular feature for a particular individual. The colour of the dots shows the feature values (red = present/1, blue = absent/0). A positive SHAP value means the predictor increases the likelihood of preterm birth, and a negative value means otherwise. The bar graph on the right side shows the mean absolute SHAP value for each feature. The feature names are displayed on the far left in order of the mean absolute value. PROM, pre-labour rupture of membrane; APH, antepartum haemorrhage; GA, gestational age; SGA, small for gestational age; w, weeks.

**Figure 6:**
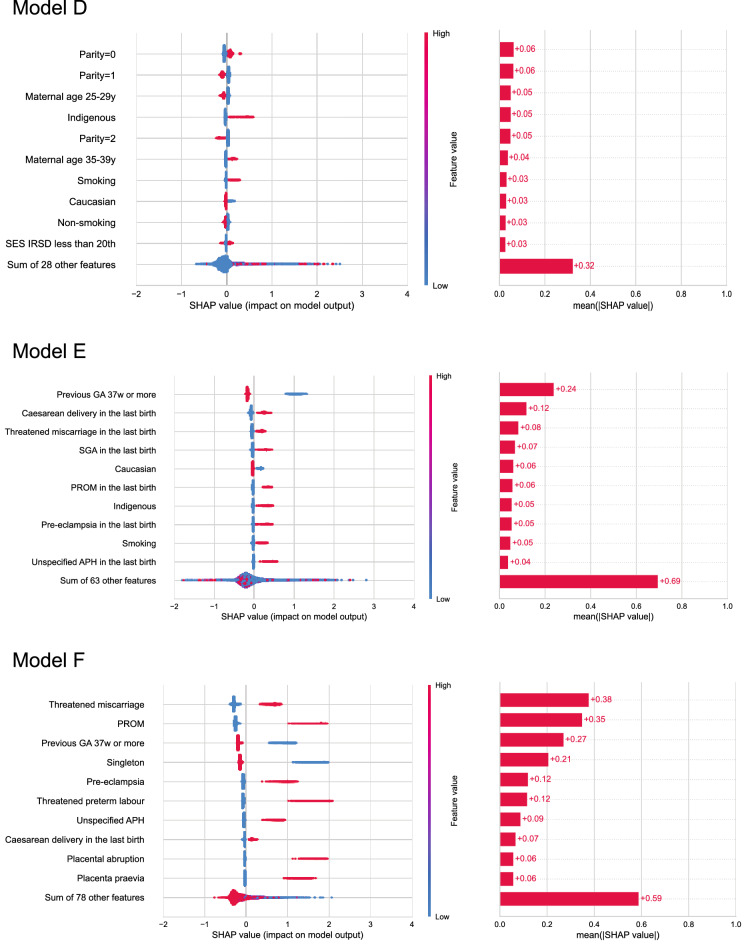
Summary plot of the SHapley Additive exPlanations (SHAP) value for models (**D**), (**E**) and (**F**) developed using the XGBoost classifier. The “beeswarm” plot on the left shows the distribution of the SHAP value (in log-odds scale) calculated using the test dataset. Each dot represents a prediction result of a particular feature for a particular individual. The colour of the dots shows the feature values (red = present/1, blue = absent/0). A positive SHAP value means the predictor increases the likelihood of preterm birth, and a negative value means otherwise. The bar graph on the right side shows the mean absolute SHAP value for each feature. The feature names are displayed on the far left in order of the mean absolute value. y, years; SES, socio-economic status; IRSD, The Index of Relative Socio-economic Disadvantage; GA, gestational age; w, weeks; SGA, small for gestational age; PROM, pre-labour rupture of membrane; APH, antepartum haemorrhage.

The SHAP value summary plots of the six models developed using the best-performing tree-based classifier (XGB) are shown in Figs. 5 and 6. Most predictors with high mean absolute SHAP were showcased in the feature importance ranking list, and the direction of association differed among the top predictors. For Model A, the presence of specific current pregnancy characteristics and complications, such as threatened miscarriage, increased the likelihood of preterm birth, whilst having a singleton birth decreased the possibility (Fig. 5). For Model B, in addition to singleton birth, a previous birth with a gestational age of 37 weeks or more lessened the risk of preterm birth. For Model C, current pregnancy conditions were noted to be the predominant drivers, and like that found in feature importance, none of the family history predictors was observed on the list. When only limited information was available at the early phase of pregnancy, as in Model D, predictors including nulliparity, maternal indigenous ethnicity, high maternal age at birth (35–39 years) and smoking contributed to the increased likelihood of preterm birth (Fig. 6). However, when past obstetric history became known, as in Model E, the additional predictors helped to explain the prediction. Of note was that the absence of a previous birth with a gestational age of 37 weeks or more heightened the risk. For Model F, the ranking and direction of association were similar to that of Model B.

## Discussion

In a large population-based cohort of Western Australia births with more than 80 thousand preterm births, we were able to correctly classify nearly half (49% at 5% FPR) of the cases using the model generated by multi-layer perceptron and a suite of routinely collected data on maternal socio-demographic, chronic medical conditions and current pregnancy characteristics and complications. Augmenting the pool of predictors with past obstetric history slightly improved the prediction performance to 53% in a sub-population of birth from multiparous women. On the other hand, we found minimal prediction improvement when family history was accounted for in the modelling that limited analysis to only births from locally born parents/grandparents (i.e., both parents and grandparents were also included in the birth perinatal registry). We also noted that omitting current pregnancy characteristics and complications significantly reduced the prediction performances, suggesting that current pregnancy information is crucial. The positive predictive values of the models generated were between 46–49% (except that of the models with only early pregnancy predictors), indicating that nearly half of the predictions of preterm birth were correct. Nevertheless, the values are influenced by the prevalence of preterm birth and should be interpreted in the context of an upper bound. In our study, given the overall prevalence of 8.6%, the highest possible value of PPV at 100% TPR and 5% FPR was 65%^33^, and, therefore, our models attained a PPV that was around 70% of the maximum possible.

When comparing the performance of the classifier in the prediction of preterm birth, we have observed that XGBoost and multi-layer perceptron consistently outperformed the rest. In the overall sample with full information on current pregnancy history (i.e., Model A), compared to logistic regression, multi-layer perceptron was able to correctly identify an additional 2659 cases (3.3%) among the 81,578 preterm births. Similarly, the TPR of multi-layer perceptron was around 2% higher than that of logistic regression in the multiparous sample (i.e., Model B). As a result, a further 782 cases were correctly detected among 37,978 preterm births. Nevertheless, despite the apparent better performance of XGBoost and multi-layer perceptron, the performance differences were small among the algorithms and were much less than the variations among the models developed.

Consistent with previous findings, our study has identified plurality (i.e., number of foetuses in a pregnancy) and specific events that occurred during current pregnancy (e.g., pre-labour rupture of membranes, placental abruption, pre-eclampsia, placenta praevia and unspecific antepartum haemorrhage) are pertinent risk factors for preterm birth, and having a history of preterm birth was also found to be informative for prediction among multiparous women^34^.

### Comparison to other studies

Studies have shown that routinely collected population-based maternal and pregnancy data can be used to predict preterm birth^17,25,35–37^. Weber et al. applied a suite of algorithms, including logistic regression and machine learning techniques, with administrative data to assess the prediction of spontaneous early preterm (< 32 weeks, prevalence 1.0%) birth among nulliparous women in California from 2007 to 2011 (n = 336,214)^36^. Their optimal model, generated by logistic regression, was able to identify 61% of cases at 36% FPR with an AUC of 0.67 (95% CI 0.65,0.68). The model’s TPR, which was approximately 20% at 5% FPR as estimated from the reported ROC curve, was lower than ours (46% at 5% FPR, Model A: LR). We have postulated that the discrepancy was partially due to differences in the study population (nulliparous women vs. all women) and outcome definition (< 32 weeks of gestation vs. < 37 weeks of gestation). Nevertheless, the data quality, instead of the prediction tools, may have played an important role in the model performance. For example, uncertainty about clinical diagnoses in hospital discharge records, and inadequate life-course risk exposure data could have affected the model's predictability. Using more than 100 maternal and neonatal features from the Iranian Maternal and neonatal (IMAN) registry, Khatibi et al. utilised machine learning algorithms and the Map-Reduce approach to develop a model for preterm birth prediction^37^. Important predictors identified include maternal age, maternal education level, city of birth, foetal sex, parity, pregnancy risk factors, gestational diabetes, cardiovascular disease, and chronic illnesses. Their best-performing model, an ensemble of decision trees, support vector machines and random forests with weighted voting, can predict preterm birth with an accuracy of 81% and an AUC of 68%. However, no other model performance statistics were presented, so a direct comparison with our study was impossible. In a more recent study, using population-based data from the United States natality datasets that provide demographic and health information on births that occurred between 2013 and 2016 (n = 12,864,146), Koivu and Sairanen employed logistic regression and machine learning methods to develop novel risk models for the prediction of pregnancies with delivery before 37 weeks of gestation (prevalence 7.4%)^25^. A gradient boosting decision tree algorithm provided the best model with a TPR of 31% at 10% FPR and an AUC of 0.67 (95% CI 0.67,0.67). The model prediction was subsequently validated by a New York City dataset collected during the years 2014 to 2016 (n = 285,871, preterm birth prevalence 6.7%), and the prediction performances were lower with this dataset (AUC 0.64 [95% CI 0.63,0.64], TPR 24% at FPR 10%). Despite the large volume of data involved in the U.S. study, their number of predictors was limited as they were derived from information in the birth certificates only. Moreover, although a handful of past obstetric history predictors (e.g., previous preterm birth) were used, they were simply dichotomised without considering that nulliparous and multiparous women were included in the modelling. These factors might explain why the results of our study (AUC 0.69 [95% CI 0.69,0.70], TPR 35% [95% CI 35,36] at FPR 10%, Model E: XGB) were better than that of their validation sample.

Other studies have employed additional risk factors collected during pregnancy to boost prediction performance. In the U.K., researchers combined proximal predictors, including cervical length (CL) and quantitative foetal fibronectin (qfFN), with other pertinent risk factors to develop a risk assessment tool for the prediction of spontaneous preterm birth (sPTB) in asymptomatic high-risk women. The research was translated into an online platform (QUiPP) for widespread use^38^. The study was recently re-evaluated with an expanded cohort (n = 1,803), and when both CL and qfFN were incorporated into the model, the AUC for the prediction of sPTB at < 37 weeks’ gestation was 0.746, and the TPR at 5% FPR was estimated to be 25%^39^. In a separate but related study where the risk-assessment tool was applied to a cohort of high-risk women with symptoms of threatened preterm labour, the AUC for the prediction of the same outcome was 0.73 (95% CI 0.63–0.83) and the TPR at 5% FPR was 20%^40^. Despite the difference in study design between the QUiPP studies and ours, it is nevertheless noteworthy to mention that the performance of their models was comparable to that of our model (Model E, MLP: AUC 0.691, TPR 23.4% at 5% FPR) in which only administrative data on socio-demographics, chronic medical conditions and past obstetric history were used. Furthermore, our predictors were not necessarily restricted to be proximal to the birth, and we utilised a more generalisable population.

### Strengths and limitations

Our study harnessed the benefits of using professionally curated population data on all births that occurred in Western Australia over three decades. The databases’ extensive time coverage allowed us to study many predictors, including familial factors that are otherwise not feasible. Furthermore, due to the administrative nature of the data, information on medical and family history was objectively collected and systematically recorded, thus avoiding the shortcomings associated with traditional health medical records and keeping missing data to a minimum.

There are limitations that need to be considered. Regarding predictors, firstly, their measurement could have changed over time due to shifts in practices. For example, there was a notable increase in the incidence of gestational diabetes in 2012–2013 due to a change in the diagnosis criteria of gestational diabetes by the Australasian Diabetes in Pregnancy Society because of the new consensus guidelines established by the International Association of Diabetes and Pregnancy Study Groups in 2010. Secondly, we do not know when risk factors first emerged, which could affect predictive performance if precise timing is required. Thirdly, as interventions and their indication were not recorded, the resulting predictions could be problematic because the treatment effects could have rendered some of the predictors less relevant. Fourthly, simplicity and interpretability can be gained by categorisation, but the ability to extract predictive information could be diminished as a result. Lastly, it is possible that machine learning was unable to detect the signal from the noise when categorised predictors were used.

Regarding the outcome and model development, firstly, spontaneous and medically indicated preterm births were collectively analysed because the study was prognostic instead of aetiologic and prediction performance (e.g., TPR and PPV) benefits from larger case numbers. Secondly, we have used class weight to improve class imbalance, but other methods, such as resampling, might be useful and haven’t yet been explored. Thirdly, the assumption of the equal importance of TPR and PPV in prediction performance is likely conservative, and a different weighting that caters for a specific clinical context might be necessary. Fourthly, the expansive time coverage of the databases allowed us to explore family factors in preterm birth prediction, but the sample size of the family linkage cohort was limited as the ancestors of the birth cohort are mostly migrants, and thus they were not included in the local birth perinatal registry. Lastly, although feature importance on model performance and the contribution of predictors to model prediction was investigated, we did not examine how the predictions using the multi-layer perceptron classifier were generated in this study. The insights gained can help promote acceptance and facilitate future model development.

### Clinical significance

The results of our study are promising, and the evidence contributes to establishing an effective prediction mechanism to identify pregnancies at elevated risk of preterm birth. The decision support system that utilises the proposed prediction algorithm can be used to inform individualised care. It aids clinicians by providing an objective evaluation of the risk of preterm birth based on administrative and clinical predictors already routinely collected in the antenatal system, thus having minimal impact on the workload. Furthermore, the assessment can be repeated when new information becomes available during the antenatal period. The longitudinal data can benefit clinicians and patients in evaluating progress and planning for preventative treatments and future interventions.

## Conclusions

We demonstrated that around half of the preterm birth could be identified antenatally at high specificity using population-based routinely collected maternal and pregnancy data. We have found that the performance of the prediction models largely depends on the available predictor pool that is individual and timing specific and is less reliant on the algorithms used for model development. Further research is needed to externally validate the models and to improve the performance using additional informative predictors and treatment indicators.

## Supplementary Information

Supplementary Information.

## Data Availability

The datasets generated and/or analysed during the current study are not publicly available due to the terms of the ethics approval granted by the Western Australian Department of Health Human Research Ethics Committee (WA DOH HREC) and the data disclosure policies of the Data Providers. The datasets may be available from the corresponding author upon request and subject to approval from the DOH HREC and relevant custodians.
