# Supplementary material for: Development of prognostic model for preterm birth using machine learning in a population-based cohort of Western Australia births between 1980 and 2015
Source: Sci Rep. 2022 Nov 9;12:19153. doi: 10.1038/s41598-022-23782-w (PMC9646808; doi:10.1038/s41598-022-23782-w)
Supplement: Supplementary file 1 — Supplementary Information. [file 41598_2022_23782_MOESM1_ESM.docx]

**Supplementary Table 1. Frequency distribution of maternal and pregnancy characteristics for births in Western Australia between 1980-2015, by preterm birth status**

|  |  | **Preterm birth** | |  |
| --- | --- | --- | --- | --- |
|  |  | **Yes**  **(n=81,794)** | **No**  **(n=876,755)** | **Total (n=958,729)** |
|  |  | n (%) | n (%) | n (%) |
| **Maternal socio-demographic factors** | | | | |
| Age, years | <20 | 5,762 (7.0) | 46,638 (5.3) | 52,400 (5.5) |
|  | 20-24 | 15,036 (18.3) | 166,404 (19.0) | 181,440 (18.9) |
|  | 25-29 | 23,896 (29.2) | 283,049 (32.3) | 306,945 (32.0) |
|  | 30-34 | 22,797 (27.8) | 252,233 (28.8) | 275,030 (28.7) |
|  | 35-39 | 11,716 (14.3) | 108,632 (12.4) | 120,348 (12.6) |
|  | ≥40 | 2,765 (3.4) | 19,793 (2.3) | 22,558 (2.4) |
|  | Unknown | <5 | 6 (0.0) | 8 (0.0) |
| Ethnicity | Caucasian | 64,510 (78.7) | 734,096 (83.7) | 798,606 (83.3) |
|  | Indigenous | 7,549 (9.2) | 41,398 (4.7) | 48,947 (5.1) |
|  | Other | 9,901 (12.1) | 101,244 (11.5) | 111,145 (11.6) |
|  | Unknown | 14 (0.0) | 17 (0.0) | 31 (0.0) |
| Socio-economic status, IRSD percentile | <20 | 18,201 (22.2) | 167,908 (19.2) | 186,109 (19.4) |
|  | 20-39 | 15,808 (19.3) | 167,810 (19.1) | 183,618 (19.2) |
|  | 40-59 | 14,875 (18.1) | 167,779 (19.1) | 182,654 (19.1) |
|  | 60-79 | 14,909 (18.2) | 166,350 (19.0) | 181,259 (18.9) |
|  | ≥80 | 14,333 (17.5) | 164,433 (18.8) | 178,766 (18.6) |
|  | Unknown | 3,848 (4.7) | 42,475 (4.8) | 46,323 (4.8) |
| Remoteness of residence | Major cities | 54,279 (66.2) | 580,890 (66.3) | 635,169 (66.3) |
|  | Regional/Remote | 19,374 (23.6) | 206,851 (23.6) | 226,225 (23.6) |
|  | Unknown | 8,321 (10.2) | 89,014 (10.2) | 97,335 (10.2) |
| Smoking | Yes | 9,748 (11.9) | 74,019 (8.4) | 83,767 (8.7) |
|  | No | 38,134 (46.5) | 409,698 (46.7) | 447,832 (46.7) |
|  | Unknown | 34,092 (41.6) | 393,038 (44.8) | 427,130 (44.6) |
| **Maternal chronic medical conditions** | | | | |
| Essential hypertension | Yes | 533 (0.7) | 2,590 (0.3) | 3,123 (0.3) |
|  | No | 81,439 (99.3) | 874,140 (99.7) | 955,579 (99.7) |
|  | Unknown | <5 | 25 (0.0) | 27 (0.0) |
| Diabetes mellitus | Yes | 1,045 (1.3) | 2,096 (0.2) | 3,141 (0.3) |
|  | No | 80,927 (98.7) | 874,634 (99.8) | 955,561 (99.7) |
|  | Unknown | <5 | 25 (0.0) | 27 (0.0) |
| Asthma | Yes | 4,551 (5.6) | 37,671 (4.3) | 42,222 (4.4) |
|  | No | 77,421 (94.4) | 839,059 (95.7) | 916,480 (95.6) |
|  | Unknown | <5 | 25 (0.0) | 27 (0.0) |
| Miscarriage | Yes | 3,070 (3.7) | 23,450 (2.7) | 26,520 (2.8) |
|  | No | 78,653 (95.9) | 848,736 (96.8) | 927,389 (96.7) |
|  | Unknown | 251 (0.3) | 4,569 (0.5) | 4,820 (0.5) |
| Obesity | Yes | 796 (1.0) | 5,498 (0.6) | 6,294 (0.7) |
|  | No | 80,927 (98.7) | 866,688 (98.9) | 947,615 (98.8) |
|  | Unknown | 251 (0.3) | 4,569 (0.5) | 4,820 (0.5) |
| Circulatory system diseases | Yes | 1,170 (1.4) | 7,717 (0.9) | 8,887 (0.9) |
|  | No | 80,553 (98.3) | 864,469 (98.6) | 945,022 (98.6) |
|  | Unknown | 251 (0.3) | 4,569 (0.5) | 4,820 (0.5) |
| **Current pregnancy characteristics and complications** | | | | |
| Parity | 0 | 35,977 (43.9) | 351,648 (40.1) | 387,625 (40.4) |
|  | 1 | 23,867 (29.1) | 299,429 (34.2) | 323,296 (33.7) |
|  | 2 | 12,123 (14.8) | 143,376 (16.4) | 155,499 (16.2) |
|  | ≥3 | 9,988 (12.2) | 82,202 (9.4) | 92,190 (9.6) |
|  | Unknown | 19 (0.0) | 100 (0.0) | 119 (0.0) |
| Gestational diabetes | Yes | 5,965 (7.3) | 36,990 (4.2) | 42,955 (4.5) |
|  | No | 76,007 (92.7) | 839,762 (95.8) | 915,769 (95.5) |
|  | Unknown | <5 | <5 | 5 (0.0) |
| Gestational hypertension | Yes | 2,171 (2.6) | 19,018 (2.2) | 21,189 (2.2) |
|  | No | 79,552 (97.0) | 853,168 (97.3) | 932,720 (97.3) |
|  | Unknown | 251 (0.3) | 4,569 (0.5) | 4,820 (0.5) |
| Urinary tract infection | Yes | 7,251 (8.8) | 43,402 (5.0) | 50,653 (5.3) |
|  | No | 74,721 (91.2) | 833,350 (95.0) | 908,071 (94.7) |
|  | Unknown | <5 | <5 | 5 (0.0) |
| Cancer registration | Yes | 365 (0.4) | 2,512 (0.3) | 2,877 (0.3) |
|  | No | 81,358 (99.2) | 869,674 (99.2) | 951,032 (99.2) |
|  | Unknown | 251 (0.3) | 4,569 (0.5) | 4,820 (0.5) |
| Pre-eclampsia | Yes | 12,954 (15.8) | 48,220 (5.5) | 61,174 (6.4) |
|  | No | 69,018 (84.2) | 828,532 (94.5) | 897,550 (93.6) |
|  | Unknown | <5 | <5 | 5 (0.0) |
| Threatened miscarriage | Yes | 36,850 (45.0) | 156,899 (17.9) | 193,749 (20.2) |
|  | No | 45,122 (55.0) | 719,853 (82.1) | 764,975 (79.8) |
|  | Unknown | <5 | <5 | 5 (0.0) |
| Placenta praevia | Yes | 2,958 (3.6) | 6,840 (0.8) | 9,798 (1.0) |
|  | No | 79,014 (96.4) | 869,912 (99.2) | 948,926 (99.0) |
|  | Unknown | <5 | <5 | 5 (0.0) |
| Placental abruption | Yes | 3,861 (4.7) | 4,105 (0.5) | 7,966 (0.8) |
|  | No | 78,111 (95.3) | 872,647 (99.5) | 950,758 (99.2) |
|  | Unknown | <5 | <5 | 5 (0.0) |
| Pre-labour rupture of membranes | Yes | 23,244 (28.4) | 42,196 (4.8) | 65,440 (6.8) |
|  | No | 58,728 (71.6) | 834,556 (95.2) | 893,284 (93.2) |
|  | Unknown | <5 | <5 | 5 (0.0) |
| Unspecified antepartum haemorrhage | Yes | 8,212 (10.0) | 25,402 (2.9) | 33,614 (3.5) |
|  | No | 73,760 (90.0) | 851,350 (97.1) | 925,110 (96.5) |
|  | Unknown | <5 | <5 | 5 (0.0) |
| Threatened preterm labour | Yes | 7,417 (9.0) | 6,765 (0.8) | 14,182 (1.5) |
|  | No | 74,507 (90.9) | 869,965 (99.2) | 944,472 (98.5) |
|  | Unknown | 50 (0.1) | 25 (0.0) | 75 (0.0) |
| Uterine rupture | Yes | 30 (0.0) | 70 (0.0) | 100 (0.0) |
|  | No | 81,693 (99.7) | 872,116 (99.5) | 953,809 (99.5) |
|  | Unknown | 251 (0.3) | 4,569 (0.5) | 4,820 (0.5) |
| Birth year | 1980-1984 | 8,176 (10.0) | 101,128 (11.5) | 109,304 (11.4) |
|  | 1985-1989 | 9,679 (11.8) | 112,282 (12.8) | 121,961 (12.7) |
|  | 1990-1994 | 10,367 (12.6) | 116,689 (13.3) | 127,056 (13.3) |
|  | 1995-1999 | 10,882 (13.3) | 116,798 (13.3) | 127,680 (13.3) |
|  | 2000-2004 | 11,321 (13.8) | 113,789 (13.0) | 125,110 (13.0) |
|  | 2005-2009 | 13,496 (16.5) | 134,030 (15.3) | 147,526 (15.4) |
|  | 2010-2015 | 18,053 (22.0) | 182,039 (20.8) | 200,092 (20.9) |
| Plurality | Singleton | 66,318 (80.9) | 865,713 (98.7) | 932,031 (97.2) |
|  | Twin | 14,729 (18.0) | 11,018 (1.3) | 25,747 (2.7) |
|  | Multiple (>2) | 927 (1.1) | 24 (0.0) | 951 (0.1) |
| Small-for-gestational age | Yes | 8,454 (10.3) | 82,824 (9.4) | 91,278 (9.5) |
|  | No | 73,409 (89.6) | 793,499 (90.5) | 866,908 (90.4) |
|  | Unknown | 111 (0.1) | 432 (0.0) | 543 (0.1) |
| Congenital anomalies | Yes | 8,888 (10.8) | 43,308 (4.9) | 52,196 (5.4) |
|  | No | 73,086 (89.2) | 833,447 (95.1) | 906,533 (94.6) |
| **Past obstetric history** | | | | |
| Gestational diabetes | Nulliparous | 35,977 (43.9) | 351,648 (40.1) | 387,625 (40.4) |
|  | First birth, parity>0 | 7,645 (9.3) | 95,124 (10.8) | 102,769 (10.7) |
|  | No | 36,559 (44.6) | 416,618 (47.5) | 453,177 (47.3) |
|  | Yes, in an earlier birth | 277 (0.3) | 1,903 (0.2) | 2,180 (0.2) |
|  | Yes, in last birth | 1,500 (1.8) | 10,836 (1.2) | 12,336 (1.3) |
|  | Unknown | 16 (0.0) | 626 (0.1) | 642 (0.1) |
| Gestational hypertension | Nulliparous | 35,977 (43.9) | 351,648 (40.1) | 387,625 (40.4) |
|  | First birth, parity>0 | 7,645 (9.3) | 95,124 (10.8) | 102,769 (10.7) |
|  | No | 36,716 (44.8) | 416,489 (47.5) | 453,205 (47.3) |
|  | Yes, in an earlier birth | 549 (0.7) | 3,326 (0.4) | 3,875 (0.4) |
|  | Yes, in last birth | 1,054 (1.3) | 9,454 (1.1) | 10,508 (1.1) |
|  | Unknown | 33 (0.0) | 714 (0.1) | 747 (0.1) |
| Urinary tract infection | Nulliparous | 35,977 (43.9) | 351,648 (40.1) | 387,625 (40.4) |
|  | First birth, parity>0 | 7,645 (9.3) | 95,124 (10.8) | 102,769 (10.7) |
|  | No | 32,812 (40.0) | 390,931 (44.6) | 423,743 (44.2) |
|  | Yes, in an earlier birth | 2,059 (2.5) | 13,508 (1.5) | 15,567 (1.6) |
|  | Yes, in last birth | 3,466 (4.2) | 24,920 (2.8) | 28,386 (3.0) |
|  | Unknown | 15 (0.0) | 624 (0.1) | 639 (0.1) |
| Cancer registration | Nulliparous | 35,977 (43.9) | 351,648 (40.1) | 387,625 (40.4) |
|  | First birth, parity>0 | 7,645 (9.3) | 95,124 (10.8) | 102,769 (10.7) |
|  | No | 38,167 (46.6) | 427,986 (48.8) | 466,153 (48.6) |
|  | Yes, in an earlier birth | 48 (0.1) | 323 (0.0) | 371 (0.0) |
|  | Yes, in last birth | 104 (0.1) | 960 (0.1) | 1,064 (0.1) |
|  | Unknown | 33 (0.0) | 714 (0.1) | 747 (0.1) |
| Pre-eclampsia | Nulliparous | 35,977 (43.9) | 351,648 (40.1) | 387,625 (40.4) |
|  | First birth, parity>0 | 7,645 (9.3) | 95,124 (10.8) | 102,769 (10.7) |
|  | No | 32,403 (39.5) | 385,953 (44.0) | 418,356 (43.6) |
|  | Yes, in an earlier birth | 1,656 (2.0) | 13,110 (1.5) | 14,766 (1.5) |
|  | Yes, in last birth | 4,279 (5.2) | 30,293 (3.5) | 34,572 (3.6) |
|  | Unknown | 14 (0.0) | 627 (0.1) | 641 (0.1) |
| Threatened miscarriage | Nulliparous | 35,977 (43.9) | 351,648 (40.1) | 387,625 (40.4) |
|  | First birth, parity>0 | 7,645 (9.3) | 95,124 (10.8) | 102,769 (10.7) |
|  | No | 23,222 (28.3) | 314,225 (35.8) | 337,447 (35.2) |
|  | Yes, in an earlier birth | 3,774 (4.6) | 29,703 (3.4) | 33,477 (3.5) |
|  | Yes, in last birth | 11,341 (13.8) | 85,450 (9.7) | 96,791 (10.1) |
|  | Unknown | 15 (0.0) | 605 (0.1) | 620 (0.1) |
| Placenta praevia | Nulliparous | 35,977 (43.9) | 351,648 (40.1) | 387,625 (40.4) |
|  | First birth, parity>0 | 7,645 (9.3) | 95,124 (10.8) | 102,769 (10.7) |
|  | No | 37,592 (45.9) | 424,672 (48.4) | 462,264 (48.2) |
|  | Yes, in an earlier birth | 228 (0.3) | 1,247 (0.1) | 1,475 (0.2) |
|  | Yes, in last birth | 516 (0.6) | 3,436 (0.4) | 3,952 (0.4) |
|  | Unknown | 16 (0.0) | 628 (0.1) | 644 (0.1) |
| Placental abruption | Nulliparous | 35,977 (43.9) | 351,648 (40.1) | 387,625 (40.4) |
|  | First birth, parity>0 | 7,645 (9.3) | 95,124 (10.8) | 102,769 (10.7) |
|  | No | 36,991 (45.1) | 424,225 (48.4) | 461,216 (48.1) |
|  | Yes, in an earlier birth | 440 (0.5) | 1,823 (0.2) | 2,263 (0.2) |
|  | Yes, in last birth | 905 (1.1) | 3,308 (0.4) | 4,213 (0.4) |
|  | Unknown | 16 (0.0) | 627 (0.1) | 643 (0.1) |
| Pre-labour rupture of membranes | Nulliparous | 35,977 (43.9) | 351,648 (40.1) | 387,625 (40.4) |
|  | First birth, parity>0 | 7,645 (9.3) | 95,124 (10.8) | 102,769 (10.7) |
|  | No | 31,142 (38.0) | 389,635 (44.4) | 420,777 (43.9) |
|  | Yes, in an earlier birth | 1,991 (2.4) | 11,855 (1.4) | 13,846 (1.4) |
|  | Yes, in last birth | 5,203 (6.3) | 27,877 (3.2) | 33,080 (3.5) |
|  | Unknown | 16 (0.0) | 616 (0.1) | 632 (0.1) |
| Unspecified antepartum haemorrhage | Nulliparous | 35,977 (43.9) | 351,648 (40.1) | 387,625 (40.4) |
|  | First birth, parity>0 | 7,645 (9.3) | 95,124 (10.8) | 102,769 (10.7) |
|  | No | 34,583 (42.2) | 408,698 (46.6) | 443,281 (46.2) |
|  | Yes, in an earlier birth | 1,275 (1.6) | 6,731 (0.8) | 8,006 (0.8) |
|  | Yes, in last birth | 2,478 (3.0) | 13,936 (1.6) | 16,414 (1.7) |
|  | Unknown | 16 (0.0) | 618 (0.1) | 634 (0.1) |
| Threatened preterm labour | Nulliparous | 35,977 (43.9) | 351,648 (40.1) | 387,625 (40.4) |
|  | First birth, parity>0 | 7,645 (9.3) | 95,124 (10.8) | 102,769 (10.7) |
|  | No | 36,420 (44.4) | 423,286 (48.3) | 459,706 (47.9) |
|  | Yes, in an earlier birth | 497 (0.6) | 1,872 (0.2) | 2,369 (0.2) |
|  | Yes, in last birth | 1,359 (1.7) | 4,288 (0.5) | 5,647 (0.6) |
|  | Unknown | 76 (0.1) | 537 (0.1) | 613 (0.1) |
| Uterine rupture | Nulliparous | 35,977 (43.9) | 351,648 (40.1) | 387,625 (40.4) |
|  | First birth, parity>0 | 7,645 (9.3) | 95,124 (10.8) | 102,769 (10.7) |
|  | No | 38,307 (46.7) | 429,243 (49.0) | 467,550 (48.8) |
|  | Yes, in an earlier birth | <5 | 8 (0.0) | 11 (0.0) |
|  | Yes, in last birth | 9 (0.0) | 18 (0.0) | 27 (0.0) |
|  | Unknown | 33 (0.0) | 714 (0.1) | 747 (0.1) |
| Caesarean section delivery | Nulliparous | 35,977 (43.9) | 351,648 (40.1) | 387,625 (40.4) |
|  | First birth, parity>0 | 7,645 (9.3) | 95,124 (10.8) | 102,769 (10.7) |
|  | No | 26,238 (32.0) | 334,494 (38.2) | 360,732 (37.6) |
|  | Yes, in an earlier birth | 940 (1.1) | 5,855 (0.7) | 6,795 (0.7) |
|  | Yes, in last birth | 11,110 (13.6) | 89,191 (10.2) | 100,301 (10.5) |
|  | Unknown | 64 (0.1) | 443 (0.1) | 507 (0.1) |
| Stillbirth | Nulliparous | 35,977 (43.9) | 351,648 (40.1) | 387,625 (40.4) |
|  | First birth, parity>0 | 7,645 (9.3) | 95,124 (10.8) | 102,769 (10.7) |
|  | No | 36,517 (44.5) | 422,854 (48.2) | 459,371 (47.9) |
|  | Yes, in an earlier birth | 608 (0.7) | 3,163 (0.4) | 3,771 (0.4) |
|  | Yes, in last birth | 1,208 (1.5) | 3,866 (0.4) | 5,074 (0.5) |
|  | Unknown | 19 (0.0) | 100 (0.0) | 119 (0.0) |
| Gestational age of last birth, weeks | <28 | 1,329 (1.6) | 2,941 (0.3) | 4,270 (0.4) |
|  | 28-31 | 1,149 (1.4) | 2,198 (0.3) | 3,347 (0.3) |
|  | 32-36 | 7,323 (8.9) | 21,418 (2.4) | 28,741 (3.0) |
|  | ≥37 | 28,294 (34.5) | 401,534 (45.8) | 429,828 (44.8) |
|  | Unknown | 43,879 (53.5) | 448,664 (51.2) | 492,543 (51.4) |
| Small-for-gestational age | Nulliparous | 35,977 (43.9) | 351,648 (40.1) | 387,625 (40.4) |
|  | First birth, parity>0 | 7,645 (9.3) | 95,124 (10.8) | 102,769 (10.7) |
|  | No | 30,018 (36.6) | 364,541 (41.6) | 394,559 (41.2) |
|  | Yes, in an earlier birth | 2,562 (3.1) | 19,046 (2.2) | 21,608 (2.3) |
|  | Yes, in last birth | 5,519 (6.7) | 44,320 (5.1) | 49,839 (5.2) |
|  | Unknown | 253 (0.3) | 2,076 (0.2) | 2,329 (0.2) |
| Congenital anomalies | Yes | 3,936 (4.8) | 34,971 (4.0) | 38,907 (4.1) |
|  | No | 34,397 (42.0) | 394,912 (45.0) | 429,309 (44.8) |
|  | Unknown | 43,641 (53.2) | 446,872 (51.0) | 490,513 (51.2) |
| **Parent’s birth outcomes** | | | | |
| Preterm birth | Yes | 1,555 (1.9) | 11,319 (1.3) | 12,874 (1.3) |
|  | No | 11,504 (14.0) | 113,254 (12.9) | 124,758 (13.0) |
|  | Unknown | 68,915 (84.1) | 752,182 (85.8) | 821,097 (85.6) |
| Small-for-gestational age | Yes | 2,509 (3.1) | 19,763 (2.3) | 22,272 (2.3) |
|  | No | 10,538 (12.9) | 104,662 (11.9) | 115,200 (12.0) |
|  | Unknown | 68,927 (84.1) | 752,330 (85.8) | 821,257 (85.7) |
| Congenital anomalies | Yes | 676 (0.8) | 6,044 (0.7) | 6,720 (0.7) |
|  | No | 12,606 (15.4) | 120,501 (13.7) | 133,107 (13.9) |
|  | Unknown | 68,692 (83.8) | 750,210 (85.6) | 818,902 (85.4) |
| **Grandmother’s chronic medical conditions and obstetric history** | | | | |
| Essential hypertension | Yes | 20 (0.0) | 139 (0.0) | 159 (0.0) |
|  | No | 13,258 (16.2) | 126,388 (14.4) | 139,646 (14.6) |
|  | Unknown | 68,696 (83.8) | 750,228 (85.6) | 818,924 (85.4) |
| Diabetes mellitus | Yes | 35 (0.0) | 147 (0.0) | 182 (0.0) |
|  | No | 13,243 (16.2) | 126,380 (14.4) | 139,623 (14.6) |
|  | Unknown | 68,696 (83.8) | 750,228 (85.6) | 818,924 (85.4) |
| Asthma | Yes | 222 (0.3) | 1,869 (0.2) | 2,091 (0.2) |
|  | No | 13,056 (15.9) | 124,660 (14.2) | 137,716 (14.4) |
|  | Unknown | 68,696 (83.8) | 750,226 (85.6) | 818,922 (85.4) |
| Miscarriage | Yes | 478 (0.6) | 4,016 (0.5) | 4,494 (0.5) |
|  | No | 12,759 (15.6) | 121,960 (13.9) | 134,719 (14.1) |
|  | Unknown | 68,737 (83.9) | 750,779 (85.6) | 819,516 (85.5) |
| Circulatory system disease | Yes | 146 (0.2) | 1,124 (0.1) | 1,270 (0.1) |
|  | No | 13,090 (16.0) | 124,853 (14.2) | 137,943 (14.4) |
|  | Unknown | 68,738 (83.9) | 750,778 (85.6) | 819,516 (85.5) |
| Gestational diabetes | Yes | 140 (0.2) | 1,040 (0.1) | 1,180 (0.1) |
|  | No | 13,138 (16.0) | 125,487 (14.3) | 138,625 (14.5) |
|  | Unknown | 68,696 (83.8) | 750,228 (85.6) | 818,924 (85.4) |
| Gestational hypertension | Yes | 14 (0.0) | 165 (0.0) | 179 (0.0) |
|  | No | 13,222 (16.1) | 125,811 (14.3) | 139,033 (14.5) |
|  | Unknown | 68,738 (83.9) | 750,779 (85.6) | 819,517 (85.5) |
| Urinary tract infection | Yes | 1,449 (1.8) | 11,526 (1.3) | 12,975 (1.4) |
|  | No | 11,829 (14.4) | 115,001 (13.1) | 126,830 (13.2) |
|  | Unknown | 68,696 (83.8) | 750,228 (85.6) | 818,924 (85.4) |
| Cancer registration | Yes | 16 (0.0) | 135 (0.0) | 151 (0.0) |
|  | No | 13,220 (16.1) | 125,841 (14.4) | 139,061 (14.5) |
|  | Unknown | 68,738 (83.9) | 750,779 (85.6) | 819,517 (85.5) |
| Pre-eclampsia | Yes | 1,562 (1.9) | 14,357 (1.6) | 15,919 (1.7) |
|  | No | 11,716 (14.3) | 112,172 (12.8) | 123,888 (12.9) |
|  | Unknown | 68,696 (83.8) | 750,226 (85.6) | 818,922 (85.4) |
| Threatened miscarriage | Yes | 3,663 (4.5) | 31,606 (3.6) | 35,269 (3.7) |
|  | No | 9,615 (11.7) | 94,923 (10.8) | 104,538 (10.9) |
|  | Unknown | 68,696 (83.8) | 750,226 (85.6) | 818,922 (85.4) |
| Placenta praevia | Yes | 139 (0.2) | 1,268 (0.1) | 1,407 (0.1) |
|  | No | 13,139 (16.0) | 125,259 (14.3) | 138,398 (14.4) |
|  | Unknown | 68,696 (83.8) | 750,228 (85.6) | 818,924 (85.4) |
| Placental abruption | Yes | 170 (0.2) | 1,582 (0.2) | 1,752 (0.2) |
|  | No | 13,108 (16.0) | 124,945 (14.3) | 138,053 (14.4) |
|  | Unknown | 68,696 (83.8) | 750,228 (85.6) | 818,924 (85.4) |
| Pre-labour rupture of membranes | Yes | 804 (1.0) | 6,025 (0.7) | 6,829 (0.7) |
|  | No | 12,474 (15.2) | 120,502 (13.7) | 132,976 (13.9) |
|  | Unknown | 68,696 (83.8) | 750,228 (85.6) | 818,924 (85.4) |
| Unspecified antepartum haemorrhage | Yes | 541 (0.7) | 4,786 (0.5) | 5,327 (0.6) |
|  | No | 12,737 (15.5) | 121,741 (13.9) | 134,478 (14.0) |
|  | Unknown | 68,696 (83.8) | 750,228 (85.6) | 818,924 (85.4) |
| Stillbirth | Yes | 0.0 (0.0) | 18 (0.0) | 18 (0.0) |
|  | No | 13,282.0 (16.2) | 126,527 (14.4) | 139,809 (14.6) |
|  | Unknown | 68,692.0 (83.8) | 750,210 (85.6) | 818,902 (85.4) |

IRSD, The Index of Relative Socio-economic Disadvantage

Notes: Percentages may not add up to 100% due to rounding. Coding for pregnancy history: Nulliparous - parity=0; First birth, parity>0 – no previous birth records exist as this was the first birth in the study period for a non-nulliparous mother; No – previous birth records exist and there is no history of the condition; Yes, in an earlier birth – the condition was present in an earlier birth; Yes, in last birth – the condition was present in the birth prior to the current one; Unknown – unknown due to missing value. Results with cell size less than 5 (excluding zero) have been presented as “<5” in accordance with the practice code for the use of personal health information provided by the WA Department of Health.

**Supplementary Table 2. Frequency distribution of maternal and pregnancy characteristics for births in Western Australia between 1980-2015, by birth decades**

|  |  | **Birth decade** | | | |  |
| --- | --- | --- | --- | --- | --- | --- |
|  |  | **1980-1989 (n=231,265)** | **1990-1999 (n=254,736)** | **2000-2009 (n=272,636)** | **2010-2015 (n=200,092)** | **Total**  **(n=958,729)** |
|  |  | n (%) | n (%) | n (%) | n (%) | n (%) |
| **Maternal socio-demographic factors** | | | | |  |  |
| Age, years | <20 | 15,458 (6.7) | 15,292 (6.0) | 14,435 (5.3) | 7,215 (3.6) | 52,400 (5.5) |
|  | 20-24 | 61,003 (26.4) | 48,709 (19.1) | 43,389 (15.9) | 28,339 (14.2) | 181,440 (18.9) |
|  | 25-29 | 89,163 (38.6) | 85,453 (33.5) | 75,548 (27.7) | 56,781 (28.4) | 306,945 (32.0) |
|  | 30-34 | 50,000 (21.6) | 73,131 (28.7) | 86,334 (31.7) | 65,565 (32.8) | 275,030 (28.7) |
|  | 35-39 | 13,725 (5.9) | 27,806 (10.9) | 44,450 (16.3) | 34,367 (17.2) | 120,348 (12.6) |
|  | ≥40 | 1,913 (0.8) | 4,345 (1.7) | 8,478 (3.1) | 7,822 (3.9) | 22,558 (2.4) |
|  | Unknown | <5 | 0 (0.0) | <5 | <5 | 8 (0.0) |
| Ethnicity | Caucasian | 207,408 (89.7) | 220,909 (86.7) | 225,285 (82.6) | 145,004 (72.5) | 798,606 (83.3) |
|  | Indigenous | 12,044 (5.2) | 14,953 (5.9) | 16,865 (6.2) | 5,085 (2.5) | 48,947 (5.1) |
|  | Other | 11,811 (5.1) | 18,872 (7.4) | 30,483 (11.2) | 49,979 (25.0) | 111,145 (11.6) |
|  | Unknown | <5 | <5 | <5 | 24 (0.0) | 31 (0.0) |
| Socio-economic status, IRSD percentile | <20 | 51,747 (22.4) | 55,345 (21.7) | 50,141 (18.4) | 28,876 (14.4) | 186,109 (19.4) |
|  | 20-39 | 46,098 (19.9) | 53,513 (21.0) | 51,991 (19.1) | 32,016 (16.0) | 183,618 (19.2) |
|  | 40-59 | 42,219 (18.3) | 50,717 (19.9) | 50,743 (18.6) | 38,975 (19.5) | 182,654 (19.1) |
|  | 60-79 | 34,415 (14.9) | 42,606 (16.7) | 56,133 (20.6) | 48,105 (24.0) | 181,259 (18.9) |
|  | ≥80 | 36,492 (15.8) | 40,387 (15.9) | 55,887 (20.5) | 46,000 (23.0) | 178,766 (18.6) |
|  | Unknown | 20,294 (8.8) | 12,168 (4.8) | 7,741 (2.8) | 6,120 (3.1) | 46,323 (4.8) |
| Remoteness of residence | Major cities | 142,823 (61.8) | 164,062 (64.4) | 182,781 (67.0) | 145,503 (72.7) | 635,169 (66.3) |
|  | Regional/Remote | 52,803 (22.8) | 63,288 (24.8) | 68,040 (25.0) | 42,094 (21.0) | 226,225 (23.6) |
|  | Unknown | 35,639 (15.4) | 27,386 (10.8) | 21,815 (8.0) | 12,495 (6.2) | 97,335 (10.2) |
| Smoking | Yes | <5 | 13,237 (5.2) | 48,394 (17.8) | 22,135 (11.1) | 83,767 (8.7) |
|  | No | 69 (0.0) | 45,616 (17.9) | 224,228 (82.2) | 177,919 (88.9) | 447,832 (46.7) |
|  | Unknown | 231,195 (100.0) | 195,883 (76.9) | 14 (0.0) | 38 (0.0) | 427,130 (44.6) |
| **Maternal chronic medical conditions** | | | | |  |  |
| Essential hypertension | Yes | 177 (0.1) | 279 (0.1) | 1,426 (0.5) | 1,241 (0.6) | 3,123 (0.3) |
|  | No | 231,074 (99.9) | 254,456 (99.9) | 271,205 (99.5) | 198,844 (99.4) | 955,579 (99.7) |
|  | Unknown | 14 (0.0) | <5 | 5 (0.0) | 7 (0.0) | 27 (0.0) |
| Diabetes mellitus | Yes | 103 (0.0) | 863 (0.3) | 1,106 (0.4) | 1,069 (0.5) | 3,141 (0.3) |
|  | No | 231,148 (99.9) | 253,872 (99.7) | 271,525 (99.6) | 199,016 (99.5) | 955,561 (99.7) |
|  | Unknown | 14 (0.0) | <5 | 5 (0.0) | 7 (0.0) | 27 (0.0) |
| Asthma | Yes | 1,891 (0.8) | 8,084 (3.2) | 18,368 (6.7) | 13,879 (6.9) | 42,222 (4.4) |
|  | No | 229,360 (99.2) | 246,651 (96.8) | 254,263 (93.3) | 186,206 (93.1) | 916,480 (95.6) |
|  | Unknown | 14 (0.0) | <5 | 5 (0.0) | 7 (0.0) | 27 (0.0) |
| Miscarriage | Yes | 4,486 (1.9) | 9,829 (3.9) | 7,857 (2.9) | 4,348 (2.2) | 26,520 (2.8) |
|  | No | 225,516 (97.5) | 244,029 (95.8) | 263,701 (96.7) | 194,143 (97.0) | 927,389 (96.7) |
|  | Unknown | 1,263 (0.5) | 878 (0.3) | 1,078 (0.4) | 1,601 (0.8) | 4,820 (0.5) |
| Obesity | Yes | 0 (0.0) | 625 (0.2) | 3,177 (1.2) | 2,492 (1.2) | 6,294 (0.7) |
|  | No | 230,002 (99.5) | 253,233 (99.4) | 268,381 (98.4) | 195,999 (98.0) | 947,615 (98.8) |
|  | Unknown | 1,263 (0.5) | 878 (0.3) | 1,078 (0.4) | 1,601 (0.8) | 4,820 (0.5) |
| Circulatory system diseases | Yes | 1,302 (0.6) | 4,916 (1.9) | 2,224 (0.8) | 445 (0.2) | 8,887 (0.9) |
|  | No | 228,700 (98.9) | 248,942 (97.7) | 269,334 (98.8) | 198,046 (99.0) | 945,022 (98.6) |
|  | Unknown | 1,263 (0.5) | 878 (0.3) | 1,078 (0.4) | 1,601 (0.8) | 4,820 (0.5) |
| **Current pregnancy characteristics and complications** | | | | |  |  |
| Parity | 0 | 89,873 (38.9) | 100,263 (39.4) | 112,370 (41.2) | 85,119 (42.5) | 387,625 (40.4) |
|  | 1 | 77,516 (33.5) | 85,214 (33.5) | 91,913 (33.7) | 68,653 (34.3) | 323,296 (33.7) |
|  | 2 | 41,311 (17.9) | 43,078 (16.9) | 41,723 (15.3) | 29,387 (14.7) | 155,499 (16.2) |
|  | ≥3 | 22,475 (9.7) | 26,179 (10.3) | 26,627 (9.8) | 16,909 (8.5) | 92,190 (9.6) |
|  | Unknown | 90 (0.0) | <5 | <5 | 24 (0.0) | 119 (0.0) |
| Gestational diabetes | Yes | 976 (0.4) | 6,498 (2.6) | 12,635 (4.6) | 22,846 (11.4) | 42,955 (4.5) |
|  | No | 230,289 (99.6) | 248,238 (97.4) | 259,999 (95.4) | 177,243 (88.6) | 915,769 (95.5) |
|  | Unknown | 0 (0.0) | 0 (0.0) | <5 | <5 | 5 (0.0) |
| Gestational hypertension | Yes | 23 (0.0) | 3,419 (1.3) | 11,871 (4.4) | 5,876 (2.9) | 21,189 (2.2) |
|  | No | 229,979 (99.4) | 250,439 (98.3) | 259,687 (95.3) | 192,615 (96.3) | 932,720 (97.3) |
|  | Unknown | 1,263 (0.5) | 878 (0.3) | 1,078 (0.4) | 1,601 (0.8) | 4,820 (0.5) |
| Urinary tract infection | Yes | 11,727 (5.1) | 18,067 (7.1) | 13,475 (4.9) | 7,384 (3.7) | 50,653 (5.3) |
|  | No | 219,538 (94.9) | 236,669 (92.9) | 259,159 (95.1) | 192,705 (96.3) | 908,071 (94.7) |
|  | Unknown | 0 (0.0) | 0 (0.0) | <5 | <5 | 5 (0.0) |
| Cancer registration | Yes | 196 (0.1) | 789 (0.3) | 1,160 (0.4) | 732 (0.4) | 2,877 (0.3) |
|  | No | 229,806 (99.4) | 253,069 (99.3) | 270,398 (99.2) | 197,759 (98.8) | 951,032 (99.2) |
|  | Unknown | 1,263 (0.5) | 878 (0.3) | 1,078 (0.4) | 1,601 (0.8) | 4,820 (0.5) |
| Pre-eclampsia | Yes | 19,389 (8.4) | 20,593 (8.1) | 14,009 (5.1) | 7,183 (3.6) | 61,174 (6.4) |
|  | No | 211,876 (91.6) | 234,143 (91.9) | 258,625 (94.9) | 192,906 (96.4) | 897,550 (93.6) |
|  | Unknown | 0 (0.0) | 0 (0.0) | <5 | <5 | 5 (0.0) |
| Threatened miscarriage | Yes | 45,223 (19.6) | 57,805 (22.7) | 60,614 (22.2) | 30,107 (15.0) | 193,749 (20.2) |
|  | No | 186,042 (80.4) | 196,931 (77.3) | 212,020 (77.8) | 169,982 (85.0) | 764,975 (79.8) |
|  | Unknown | 0 (0.0) | 0 (0.0) | <5 | <5 | 5 (0.0) |
| Placenta praevia | Yes | 1,903 (0.8) | 2,500 (1.0) | 3,257 (1.2) | 2,138 (1.1) | 9,798 (1.0) |
|  | No | 229,362 (99.2) | 252,236 (99.0) | 269,377 (98.8) | 197,951 (98.9) | 948,926 (99.0) |
|  | Unknown | 0 (0.0) | 0 (0.0) | <5 | <5 | 5 (0.0) |
| Placental abruption | Yes | 2,465 (1.1) | 2,291 (0.9) | 1,951 (0.7) | 1,259 (0.6) | 7,966 (0.8) |
|  | No | 228,800 (98.9) | 252,445 (99.1) | 270,683 (99.3) | 198,830 (99.4) | 950,758 (99.2) |
|  | Unknown | 0 (0.0) | 0 (0.0) | <5 | <5 | 5 (0.0) |
| Pre-labour rupture of membranes | Yes | 8,566 (3.7) | 15,191 (6.0) | 25,306 (9.3) | 16,377 (8.2) | 65,440 (6.8) |
|  | No | 222,699 (96.3) | 239,545 (94.0) | 247,328 (90.7) | 183,712 (91.8) | 893,284 (93.2) |
|  | Unknown | 0 (0.0) | 0 (0.0) | <5 | <5 | 5 (0.0) |
| Unspecified antepartum haemorrhage | Yes | 6,519 (2.8) | 10,094 (4.0) | 10,046 (3.7) | 6,955 (3.5) | 33,614 (3.5) |
|  | No | 224,746 (97.2) | 244,642 (96.0) | 262,588 (96.3) | 193,134 (96.5) | 925,110 (96.5) |
|  | Unknown | 0 (0.0) | 0 (0.0) | <5 | <5 | 5 (0.0) |
| Threatened preterm labour | Yes | 0 (0.0) | 1,607 (0.6) | 7,381 (2.7) | 5,194 (2.6) | 14,182 (1.5) |
|  | No | 231,257 (100.0) | 253,114 (99.4) | 265,241 (97.3) | 194,860 (97.4) | 944,472 (98.5) |
|  | Unknown | 8 (0.0) | 15 (0.0) | 14 (0.0) | 38 (0.0) | 75 (0.0) |
| Uterine rupture | Yes | 25 (0.0) | 29 (0.0) | 39 (0.0) | 7 (0.0) | 100 (0.0) |
|  | No | 229,977 (99.4) | 253,829 (99.6) | 271,519 (99.6) | 198,484 (99.2) | 953,809 (99.5) |
|  | Unknown | 1,263 (0.5) | 878 (0.3) | 1,078 (0.4) | 1,601 (0.8) | 4,820 (0.5) |
| Plurality | Singleton | 225,745 (97.6) | 247,574 (97.2) | 264,192 (96.9) | 194,520 (97.2) | 932,031 (97.2) |
|  | Twin | 5,266 (2.3) | 6,830 (2.7) | 8,193 (3.0) | 5,458 (2.7) | 25,747 (2.7) |
|  | Multiple (>2) | 254 (0.1) | 332 (0.1) | 251 (0.1) | 114 (0.1) | 951 (0.1) |
| Small-for-gestational age | Yes | 26,346 (11.4) | 24,734 (9.7) | 23,359 (8.6) | 16,839 (8.4) | 91,278 (9.5) |
|  | No | 204,655 (88.5) | 229,875 (90.2) | 249,187 (91.4) | 183,191 (91.6) | 866,908 (90.4) |
|  | Unknown | 264 (0.1) | 127 (0.0) | 90 (0.0) | 62 (0.0) | 543 (0.1) |
| Preterm birth | Yes | 17,855 (7.7) | 21,249 (8.3) | 24,817 (9.1) | 18,053 (9.0) | 81,974 (8.6) |
|  | No | 213,410 (92.3) | 233,487 (91.7) | 247,819 (90.9) | 182,039 (91.0) | 876,755 (91.4) |
| Congenital anomalies | Yes | 11,167 (4.8) | 15,020 (5.9) | 15,624 (5.7) | 10,385 (5.2) | 52,196 (5.4) |
|  | No | 220,098 (95.2) | 239,716 (94.1) | 257,012 (94.3) | 189,707 (94.8) | 906,533 (94.6) |
| **Past obstetric history** | | | | |  |  |
| Gestational diabetes | Nulliparous | 89,873 (38.9) | 100,263 (39.4) | 112,370 (41.2) | 85,119 (42.5) | 387,625 (40.4) |
|  | First birth, parity>0 | 51,724 (22.4) | 17,067 (6.7) | 17,331 (6.4) | 16,647 (8.3) | 102,769 (10.7) |
|  | No | 89,327 (38.6) | 135,029 (53.0) | 136,972 (50.2) | 91,849 (45.9) | 453,177 (47.3) |
|  | Yes, in an earlier birth | 35 (0.0) | 291 (0.1) | 951 (0.3) | 903 (0.5) | 2,180 (0.2) |
|  | Yes, in last birth | 180 (0.1) | 1,929 (0.8) | 4,830 (1.8) | 5,397 (2.7) | 12,336 (1.3) |
|  | Unknown | 126 (0.1) | 157 (0.1) | 182 (0.1) | 177 (0.1) | 642 (0.1) |
| Gestational hypertension | Nulliparous | 89,873 (38.9) | 100,263 (39.4) | 112,370 (41.2) | 85,119 (42.5) | 387,625 (40.4) |
|  | First birth, parity>0 | 51,724 (22.4) | 17,067 (6.7) | 17,331 (6.4) | 16,647 (8.3) | 102,769 (10.7) |
|  | No | 89,451 (38.7) | 136,155 (53.4) | 134,706 (49.4) | 92,893 (46.4) | 453,205 (47.3) |
|  | Yes, in an earlier birth | <5 | 179 (0.1) | 1,923 (0.7) | 1,771 (0.9) | 3,875 (0.4) |
|  | Yes, in last birth | 10 (0.0) | 913 (0.4) | 6,123 (2.2) | 3,462 (1.7) | 10,508 (1.1) |
|  | Unknown | 205 (0.1) | 159 (0.1) | 183 (0.1) | 200 (0.1) | 747 (0.1) |
| Urinary tract infection | Nulliparous | 89,873 (38.9) | 100,263 (39.4) | 112,370 (41.2) | 85,119 (42.5) | 387,625 (40.4) |
|  | First birth, parity>0 | 51,724 (22.4) | 17,067 (6.7) | 17,331 (6.4) | 16,647 (8.3) | 102,769 (10.7) |
|  | No | 83,641 (36.2) | 122,586 (48.1) | 127,203 (46.7) | 90,313 (45.1) | 423,743 (44.2) |
|  | Yes, in an earlier birth | 1,274 (0.6) | 4,777 (1.9) | 6,294 (2.3) | 3,222 (1.6) | 15,567 (1.6) |
|  | Yes, in last birth | 4,627 (2.0) | 9,887 (3.9) | 9,259 (3.4) | 4,613 (2.3) | 28,386 (3.0) |
|  | Unknown | 126 (0.1) | 156 (0.1) | 179 (0.1) | 178 (0.1) | 639 (0.1) |
| Cancer registration | Nulliparous | 89,873 (38.9) | 100,263 (39.4) | 112,370 (41.2) | 85,119 (42.5) | 387,625 (40.4) |
|  | First birth, parity>0 | 51,724 (22.4) | 17,067 (6.7) | 17,331 (6.4) | 16,647 (8.3) | 102,769 (10.7) |
|  | No | 89,397 (38.7) | 136,942 (53.8) | 142,113 (52.1) | 97,701 (48.8) | 466,153 (48.6) |
|  | Yes, in an earlier birth | 9 (0.0) | 79 (0.0) | 168 (0.1) | 115 (0.1) | 371 (0.0) |
|  | Yes, in last birth | 57 (0.0) | 226 (0.1) | 471 (0.2) | 310 (0.2) | 1,064 (0.1) |
|  | Unknown | 205 (0.1) | 159 (0.1) | 183 (0.1) | 200 (0.1) | 747 (0.1) |
| Pre-eclampsia | Nulliparous | 89,873 (38.9) | 100,263 (39.4) | 112,370 (41.2) | 85,119 (42.5) | 387,625 (40.4) |
|  | First birth, parity>0 | 51,724 (22.4) | 17,067 (6.7) | 17,331 (6.4) | 16,647 (8.3) | 102,769 (10.7) |
|  | No | 79,204 (34.2) | 120,031 (47.1) | 127,793 (46.9) | 91,328 (45.6) | 418,356 (43.6) |
|  | Yes, in an earlier birth | 1,944 (0.8) | 5,191 (2.0) | 5,163 (1.9) | 2,468 (1.2) | 14,766 (1.5) |
|  | Yes, in last birth | 8,397 (3.6) | 12,027 (4.7) | 9,797 (3.6) | 4,351 (2.2) | 34,572 (3.6) |
|  | Unknown | 123 (0.1) | 157 (0.1) | 182 (0.1) | 179 (0.1) | 641 (0.1) |
| Threatened miscarriage | Nulliparous | 89,873 (38.9) | 100,263 (39.4) | 112,370 (41.2) | 85,119 (42.5) | 387,625 (40.4) |
|  | First birth, parity>0 | 51,724 (22.4) | 17,067 (6.7) | 17,331 (6.4) | 16,647 (8.3) | 102,769 (10.7) |
|  | No | 69,285 (30.0) | 96,693 (38.0) | 99,505 (36.5) | 71,964 (36.0) | 337,447 (35.2) |
|  | Yes, in an earlier birth | 3,366 (1.5) | 10,195 (4.0) | 12,079 (4.4) | 7,837 (3.9) | 33,477 (3.5) |
|  | Yes, in last birth | 16,897 (7.3) | 30,365 (11.9) | 31,178 (11.4) | 18,351 (9.2) | 96,791 (10.1) |
|  | Unknown | 120 (0.1) | 153 (0.1) | 173 (0.1) | 174 (0.1) | 620 (0.1) |
| Placenta praevia | Nulliparous | 89,873 (38.9) | 100,263 (39.4) | 112,370 (41.2) | 85,119 (42.5) | 387,625 (40.4) |
|  | First birth, parity>0 | 51,724 (22.4) | 17,067 (6.7) | 17,331 (6.4) | 16,647 (8.3) | 102,769 (10.7) |
|  | No | 88,770 (38.4) | 135,765 (53.3) | 140,829 (51.7) | 96,900 (48.4) | 462,264 (48.2) |
|  | Yes, in an earlier birth | 157 (0.1) | 450 (0.2) | 521 (0.2) | 347 (0.2) | 1,475 (0.2) |
|  | Yes, in last birth | 615 (0.3) | 1,034 (0.4) | 1,403 (0.5) | 900 (0.4) | 3,952 (0.4) |
|  | Unknown | 126 (0.1) | 157 (0.1) | 182 (0.1) | 179 (0.1) | 644 (0.1) |
| Placental abruption | Nulliparous | 89,873 (38.9) | 100,263 (39.4) | 112,370 (41.2) | 85,119 (42.5) | 387,625 (40.4) |
|  | First birth, parity>0 | 51,724 (22.4) | 17,067 (6.7) | 17,331 (6.4) | 16,647 (8.3) | 102,769 (10.7) |
|  | No | 88,130 (38.1) | 135,045 (53.0) | 140,934 (51.7) | 97,107 (48.5) | 461,216 (48.1) |
|  | Yes, in an earlier birth | 357 (0.2) | 876 (0.3) | 674 (0.2) | 356 (0.2) | 2,263 (0.2) |
|  | Yes, in last birth | 1,055 (0.5) | 1,329 (0.5) | 1,145 (0.4) | 684 (0.3) | 4,213 (0.4) |
|  | Unknown | 126 (0.1) | 156 (0.1) | 182 (0.1) | 179 (0.1) | 643 (0.1) |
| Pre-labour rupture of membranes | Nulliparous | 89,873 (38.9) | 100,263 (39.4) | 112,370 (41.2) | 85,119 (42.5) | 387,625 (40.4) |
|  | First birth, parity>0 | 51,724 (22.4) | 17,067 (6.7) | 17,331 (6.4) | 16,647 (8.3) | 102,769 (10.7) |
|  | No | 85,488 (37.0) | 126,933 (49.8) | 124,489 (45.7) | 83,867 (41.9) | 420,777 (43.9) |
|  | Yes, in an earlier birth | 873 (0.4) | 3,066 (1.2) | 5,347 (2.0) | 4,560 (2.3) | 13,846 (1.4) |
|  | Yes, in last birth | 3,181 (1.4) | 7,251 (2.8) | 12,921 (4.7) | 9,727 (4.9) | 33,080 (3.5) |
|  | Unknown | 126 (0.1) | 156 (0.1) | 178 (0.1) | 172 (0.1) | 632 (0.1) |
| Unspecified antepartum haemorrhage | Nulliparous | 89,873 (38.9) | 100,263 (39.4) | 112,370 (41.2) | 85,119 (42.5) | 387,625 (40.4) |
|  | First birth, parity>0 | 51,724 (22.4) | 17,067 (6.7) | 17,331 (6.4) | 16,647 (8.3) | 102,769 (10.7) |
|  | No | 86,717 (37.5) | 129,547 (50.9) | 134,234 (49.2) | 92,783 (46.4) | 443,281 (46.2) |
|  | Yes, in an earlier birth | 602 (0.3) | 2,407 (0.9) | 3,123 (1.1) | 1,874 (0.9) | 8,006 (0.8) |
|  | Yes, in last birth | 2,224 (1.0) | 5,297 (2.1) | 5,399 (2.0) | 3,494 (1.7) | 16,414 (1.7) |
|  | Unknown | 125 (0.1) | 155 (0.1) | 179 (0.1) | 175 (0.1) | 634 (0.1) |
| Threatened preterm labour | Nulliparous | 89,873 (38.9) | 100,263 (39.4) | 112,370 (41.2) | 85,119 (42.5) | 387,625 (40.4) |
|  | First birth, parity>0 | 51,724 (22.4) | 17,067 (6.7) | 17,331 (6.4) | 16,647 (8.3) | 102,769 (10.7) |
|  | No | 89,414 (38.7) | 137,147 (53.8) | 138,468 (50.8) | 94,677 (47.3) | 459,706 (47.9) |
|  | Yes, in an earlier birth | 0 (0.0) | 0 (0.0) | 1,127 (0.4) | 1,242 (0.6) | 2,369 (0.2) |
|  | Yes, in last birth | 0 (0.0) | 104 (0.0) | 3,219 (1.2) | 2,324 (1.2) | 5,647 (0.6) |
|  | Unknown | 254 (0.1) | 155 (0.1) | 121 (0.0) | 83 (0.0) | 613 (0.1) |
| Uterine rupture | Nulliparous | 89,873 (38.9) | 100,263 (39.4) | 112,370 (41.2) | 85,119 (42.5) | 387,625 (40.4) |
|  | First birth, parity>0 | 51,724 (22.4) | 17,067 (6.7) | 17,331 (6.4) | 16,647 (8.3) | 102,769 (10.7) |
|  | No | 89,455 (38.7) | 137,240 (53.9) | 142,738 (52.4) | 98,117 (49.0) | 467,550 (48.8) |
|  | Yes, in an earlier birth | <5 | 0 (0.0) | <5 | <5 | 11 (0.0) |
|  | Yes, in last birth | 5 (0.0) | 7 (0.0) | 10 (0.0) | 5 (0.0) | 27 (0.0) |
|  | Unknown | 205 (0.1) | 159 (0.1) | 183 (0.1) | 200 (0.1) | 747 (0.1) |
| Caesarean section delivery | Nulliparous | 89,873 (38.9) | 100,263 (39.4) | 112,370 (41.2) | 85,119 (42.5) | 387,625 (40.4) |
|  | First birth, parity>0 | 51,724 (22.4) | 17,067 (6.7) | 17,331 (6.4) | 16,647 (8.3) | 102,769 (10.7) |
|  | No | 77,064 (33.3) | 110,990 (43.6) | 104,800 (38.4) | 67,878 (33.9) | 360,732 (37.6) |
|  | Yes, in an earlier birth | 689 (0.3) | 2,305 (0.9) | 2,300 (0.8) | 1,501 (0.8) | 6,795 (0.7) |
|  | Yes, in last birth | 11,748 (5.1) | 23,953 (9.4) | 35,712 (13.1) | 28,888 (14.4) | 100,301 (10.5) |
|  | Unknown | 167 (0.1) | 158 (0.1) | 123 (0.0) | 59 (0.0) | 507 (0.1) |
| Stillbirth | Nulliparous | 89,873 (38.9) | 100,263 (39.4) | 112,370 (41.2) | 85,119 (42.5) | 387,625 (40.4) |
|  | First birth, parity>0 | 51,724 (22.4) | 17,067 (6.7) | 17,331 (6.4) | 16,647 (8.3) | 102,769 (10.7) |
|  | No | 87,813 (38.0) | 134,850 (52.9) | 140,278 (51.5) | 96,430 (48.2) | 459,371 (47.9) |
|  | Yes, in an earlier birth | 596 (0.3) | 1,141 (0.4) | 1,213 (0.4) | 821 (0.4) | 3,771 (0.4) |
|  | Yes, in last birth | 1,169 (0.5) | 1,413 (0.6) | 1,441 (0.5) | 1,051 (0.5) | 5,074 (0.5) |
|  | Unknown | 90 (0.0) | <5 | <5 | 24 (0.0) | 119 (0.0) |
| Gestational age of last birth, weeks | <28 | 871 (0.4) | 1,096 (0.4) | 1,286 (0.5) | 1,017 (0.5) | 4,270 (0.4) |
|  | 28-31 | 711 (0.3) | 976 (0.4) | 998 (0.4) | 662 (0.3) | 3,347 (0.3) |
|  | 32-36 | 5,088 (2.2) | 8,346 (3.3) | 9,161 (3.4) | 6,146 (3.1) | 28,741 (3.0) |
|  | ≥37 | 81,435 (35.2) | 126,691 (49.7) | 131,315 (48.2) | 90,387 (45.2) | 429,828 (44.8) |
|  | Unknown | 143,160 (61.9) | 117,627 (46.2) | 129,876 (47.6) | 101,880 (50.9) | 492,543 (51.4) |
| Small-for-gestational age | Nulliparous | 89,873 (38.9) | 100,263 (39.4) | 112,370 (41.2) | 85,119 (42.5) | 387,625 (40.4) |
|  | First birth, parity>0 | 51,724 (22.4) | 17,067 (6.7) | 17,331 (6.4) | 16,647 (8.3) | 102,769 (10.7) |
|  | No | 74,002 (32.0) | 114,205 (44.8) | 121,363 (44.5) | 84,989 (42.5) | 394,559 (41.2) |
|  | Yes, in an earlier birth | 2,759 (1.2) | 7,321 (2.9) | 7,343 (2.7) | 4,185 (2.1) | 21,608 (2.3) |
|  | Yes, in last birth | 11,295 (4.9) | 15,509 (6.1) | 14,021 (5.1) | 9,014 (4.5) | 49,839 (5.2) |
|  | Unknown | 1,612 (0.7) | 371 (0.1) | 208 (0.1) | 138 (0.1) | 2,329 (0.2) |
| Congenital anomalies | Yes | 5,849 (2.5) | 11,839 (4.6) | 12,974 (4.8) | 8,245 (4.1) | 38,907 (4.1) |
|  | No | 83,729 (36.2) | 125,565 (49.3) | 129,958 (47.7) | 90,057 (45.0) | 429,309 (44.8) |
|  | Unknown | 141,687 (61.3) | 117,332 (46.1) | 129,704 (47.6) | 101,790 (50.9) | 490,513 (51.2) |
| **Parent’s birth outcomes** | | | | |  |  |
| Preterm birth | Yes | 0 (0.0) | 369 (0.1) | 8,690 (3.2) | 13,213 (6.6) | 12,874 (1.3) |
|  | No | 0 (0.0) | 1,575 (0.6) | 41,857 (15.4) | 71,768 (35.9) | 124,758 (13.0) |
|  | Unknown | 231,265 (100.0) | 252,792 (99.2) | 222,089 (81.5) | 115,111 (57.5) | 821,097 (85.6) |
| Small-for-gestational age | Yes | 0 (0.0) | 369 (0.1) | 8,690 (3.2) | 13,213 (6.6) | 22,272 (2.3) |
|  | No | 0 (0.0) | 1,575 (0.6) | 41,857 (15.4) | 71,768 (35.9) | 115,200 (12.0) |
|  | Unknown | 231,265 (100.0) | 252,792 (99.2) | 222,089 (81.5) | 115,111 (57.5) | 821,257 (85.7) |
| Congenital anomalies | Yes | 0 (0.0) | 62 (0.0) | 2,223 (0.8) | 4,435 (2.2) | 6,720 (0.7) |
|  | No | 0 (0.0) | 2,028 (0.8) | 49,535 (18.2) | 81,544 (40.8) | 133,107 (13.9) |
|  | Unknown | 231,265 (100.0) | 252,646 (99.2) | 220,878 (81.0) | 114,113 (57.0) | 818,902 (85.4) |
| **Grandmother’s chronic medical conditions and obstetric history** | | | | |  |  |
| Essential hypertension | Yes | 0 (0.0) | 0 (0.0) | 56 (0.0) | 103 (0.1) | 159 (0.0) |
|  | No | 0 (0.0) | 2,090 (0.8) | 51,693 (19.0) | 85,863 (42.9) | 139,646 (14.6) |
|  | Unknown | 231,265 (100.0) | 252,646 (99.2) | 220,887 (81.0) | 114,126 (57.0) | 818,924 (85.4) |
| Diabetes mellitus | Yes | 0 (0.0) | 0 (0.0) | 52 (0.0) | 130 (0.1) | 182 (0.0) |
|  | No | 0 (0.0) | 2,090 (0.8) | 51,697 (19.0) | 85,836 (42.9) | 139,623 (14.6) |
|  | Unknown | 231,265 (100.0) | 252,646 (99.2) | 220,887 (81.0) | 114,126 (57.0) | 818,924 (85.4) |
| Asthma | Yes | 0 (0.0) | <5 | 531 (0.2) | 1,556 (0.8) | 2,091 (0.2) |
|  | No | 0 (0.0) | 2,086 (0.8) | 51,219 (18.8) | 84,411 (42.2) | 137,716 (14.4) |
|  | Unknown | 231,265 (100.0) | 252,646 (99.2) | 220,886 (81.0) | 114,125 (57.0) | 818,922 (85.4) |
| Miscarriage | Yes | 0 (0.0) | 7 (0.0) | 1,255 (0.5) | 3,232 (1.6) | 4,494 (0.5) |
|  | No | 0 (0.0) | 2,069 (0.8) | 50,263 (18.4) | 82,387 (41.2) | 134,719 (14.1) |
|  | Unknown | 231,265 (100.0) | 252,660 (99.2) | 221,118 (81.1) | 114,473 (57.2) | 819,516 (85.5) |
| Circulatory system disease | Yes | 0 (0.0) | <5 | 386 (0.1) | 880 (0.4) | 1,270 (0.1) |
|  | No | 0 (0.0) | 2,072 (0.8) | 51,131 (18.8) | 84,740 (42.4) | 137,943 (14.4) |
|  | Unknown | 231,265 (100.0) | 252,660 (99.2) | 221,119 (81.1) | 114,472 (57.2) | 819,516 (85.5) |
| Gestational diabetes | Yes | 0 (0.0) | 11 (0.0) | 319 (0.1) | 850 (0.4) | 1,180 (0.1) |
|  | No | 0 (0.0) | 2,079 (0.8) | 51,430 (18.9) | 85,116 (42.5) | 138,625 (14.5) |
|  | Unknown | 231,265 (100.0) | 252,646 (99.2) | 220,887 (81.0) | 114,126 (57.0) | 818,924 (85.4) |
| Gestational hypertension | Yes | 0 (0.0) | 0 (0.0) | 12 (0.0) | 167 (0.1) | 179 (0.0) |
|  | No | 0 (0.0) | 2,076 (0.8) | 51,505 (18.9) | 85,452 (42.7) | 139,033 (14.5) |
|  | Unknown | 231,265 (100.0) | 252,660 (99.2) | 221,119 (81.1) | 114,473 (57.2) | 819,517 (85.5) |
| Urinary tract infection | Yes | 0 (0.0) | 228 (0.1) | 5,078 (1.9) | 7,669 (3.8) | 12,975 (1.4) |
|  | No | 0 (0.0) | 1,862 (0.7) | 46,671 (17.1) | 78,297 (39.1) | 126,830 (13.2) |
|  | Unknown | 231,265 (100.0) | 252,646 (99.2) | 220,887 (81.0) | 114,126 (57.0) | 818,924 (85.4) |
| Cancer registration | Yes | 0 (0.0) | 0 (0.0) | 38 (0.0) | 113 (0.1) | 151 (0.0) |
|  | No | 0 (0.0) | 2,076 (0.8) | 51,479 (18.9) | 85,506 (42.7) | 139,061 (14.5) |
|  | Unknown | 231,265 (100.0) | 252,660 (99.2) | 221,119 (81.1) | 114,473 (57.2) | 819,517 (85.5) |
| Pre-eclampsia | Yes | 0 (0.0) | 212 (0.1) | 5,728 (2.1) | 9,979 (5.0) | 15,919 (1.7) |
|  | No | 0 (0.0) | 1,878 (0.7) | 46,023 (16.9) | 75,987 (38.0) | 123,888 (12.9) |
|  | Unknown | 231,265 (100.0) | 252,646 (99.2) | 220,885 (81.0) | 114,126 (57.0) | 818,922 (85.4) |
| Threatened miscarriage | Yes | 0 (0.0) | 426 (0.2) | 12,492 (4.6) | 22,351 (11.2) | 35,269 (3.7) |
|  | No | 0 (0.0) | 1,664 (0.7) | 39,258 (14.4) | 63,616 (31.8) | 104,538 (10.9) |
|  | Unknown | 231,265 (100.0) | 252,646 (99.2) | 220,886 (81.0) | 114,125 (57.0) | 818,922 (85.4) |
| Placenta praevia | Yes | 0 (0.0) | 12 (0.0) | 517 (0.2) | 878 (0.4) | 1,407 (0.1) |
|  | No | 0 (0.0) | 2,078 (0.8) | 51,232 (18.8) | 85,088 (42.5) | 138,398 (14.4) |
|  | Unknown | 231,265 (100.0) | 252,646 (99.2) | 220,887 (81.0) | 114,126 (57.0) | 818,924 (85.4) |
| Placental abruption | Yes | 0 (0.0) | 18 (0.0) | 683 (0.3) | 1,051 (0.5) | 1,752 (0.2) |
|  | No | 0 (0.0) | 2,072 (0.8) | 51,066 (18.7) | 84,915 (42.4) | 138,053 (14.4) |
|  | Unknown | 231,265 (100.0) | 252,646 (99.2) | 220,887 (81.0) | 114,126 (57.0) | 818,924 (85.4) |
| Pre-labour rupture of membranes | Yes | 0 (0.0) | 94 (0.0) | 2,400 (0.9) | 4,335 (2.2) | 6,829 (0.7) |
|  | No | 0 (0.0) | 1,996 (0.8) | 49,349 (18.1) | 81,631 (40.8) | 132,976 (13.9) |
|  | Unknown | 231,265 (100.0) | 252,646 (99.2) | 220,887 (81.0) | 114,126 (57.0) | 818,924 (85.4) |
| Unspecified antepartum haemorrhage | Yes | 0 (0.0) | 38 (0.0) | 1,845 (0.7) | 3,444 (1.7) | 5,327 (0.6) |
|  | No | 0 (0.0) | 2,052 (0.8) | 49,904 (18.3) | 82,522 (41.2) | 134,478 (14.0) |
|  | Unknown | 231,265 (100.0) | 252,646 (99.2) | 220,887 (81.0) | 114,126 (57.0) | 818,924 (85.4) |
| Stillbirth | Yes | 0 (0.0) | <5 | 10 (0.0) | 6 (0.0) | 18 (0.0) |
|  | No | 0 (0.0) | 2,088 (0.8) | 51,748 (19.0) | 85,973 (43.0) | 139,809 (14.6) |
|  | Unknown | 231,265 (100.0) | 252,646 (99.2) | 220,878 (81.0) | 114,113 (57.0) | 818,902 (85.4) |

IRSD, The Index of Relative Socio-economic Disadvantage

Notes: Percentages may not add up to 100% due to rounding. Coding for pregnancy history: Nulliparous - parity=0; First birth, parity>0 – no previous birth records exist as this was the first birth in the study period for a non-nulliparous mother; No – previous birth records exist and there is no history of the condition; Yes, in an earlier birth – the condition was present in an earlier birth; Yes, in last birth – the condition was present in the birth prior to the current one; Unknown – unknown due to missing value. Results with cell size less than 5 (excluding zero) have been presented as “<5” in accordance with the practice code for the use of personal health information provided by the WA Department of Health.

**Supplementary Table 3. List of predictors used for preterm birth prediction**

| **Predictor group/Predictor** | **Description/Coding** | **Data source** | **Years Available** | **Model** | | | | | |
| --- | --- | --- | --- | --- | --- | --- | --- | --- | --- |
|  |  |  |  | **A** | **B** | **C** | **D** | **E** | **F** |
| **Maternal socio-demographic factors** | | | | | | | | | |
| Age | 5-year age groups | MNS | 1980-2015 | + | + | + | + | + | + |
| Ethnicity | Caucasian, Indigenous, Other, Unknown | MNS, Indigenous status | 1980-2015 | + | + | + | + | + | + |
| Socio-economic status | Quintiles of Index of Relative Socio-economic Disadvantage | MNS | 1980-2015 | + | + | + | + | + | + |
| Remoteness of residence | Major cities, Regional/Remote, Unknown | MNS | 1980-2015 | + | + | + | + | + | + |
| Smoking | Any smoking during pregnancy | MNS | 1997-2015 | + | + | + | + | + | + |
| **Maternal chronic medical conditions** | | | | | | | | | |
| Essential hypertension | ICD-9: 401; ICD-10: I10 | HMDC, MNS | 1980-2015 | + | + | + | + | + | + |
| Diabetes mellitus | ICD-9: 250; ICD-10: E08-E11, E13 | HMDC, MNS | 1980-2015 | + | + | + | + | + | + |
| Asthma | ICD-9: 493; ICD-10: J45 | HMDC, MNS | 1980-2015 | + | + | + | + | + | + |
| Miscarriage | ICD-9: 634; ICD-10: O03 (<20 weeks’ gestation) | HMDC, MNS | 1980-2015 | + | + | + | + | + | + |
| Obesity | ICD-9: 278.0-278.03; ICD-10: E66 | HMDC, MNS | 1980-2015 | + | + | + | + | + | + |
| Circulatory system diseases | ICD-9: 390-459; ICD-10: 100-199 | HMDC, MNS | 1980-2015 | + | + | + | + | + | + |
| **Current pregnancy characteristics and complications** | | | | | | | | | |
| Parity | Number of previous live and stillbirths | MNS | 1980-2015 | + | + | + | + | + | + |
| Gestational diabetes | ICD-9: 648.8; ICD-10: O24.4 | HMDC, MNS | 1980-2015 | + | + | + |  |  | + |
| Gestational hypertension | ICD-9: 642.3; ICD-10: O13 | HMDC, MNS | 1980-2015 | + | + | + |  |  | + |
| Urinary tract infection | ICD-9: 646.6; ICD-10: O23 | HMDC, MNS | 1980-2015 | + | + | + |  |  | + |
| Cancer registration | ICD-9: 140-239; ICD-10: C00-D49 | Cancer Registry, HMDC | 1980-2015 | + | + | + |  |  | + |
| Pre-eclampsia | ICD-9: 642.4, 642.5, 642.7; ICD-10: O11, O14 | HMDC, MNS | 1980-2015 | + | + | + |  |  | + |
| Threatened miscarriage | ICD-9: 640-640.03; ICD-10: O20.0 (<20 weeks’ gestation) | HMDC, MNS | 1980-2015 | + | + | + |  |  | + |
| Placenta praevia | ICD-9: 641.0-641.1, ICD-10: O44 | HMDC, MNS | 1980-2015 | + | + | + |  |  | + |
| Placental abruption | ICD-9: 641.2; ICD-10: O45 | HMDC, MNS | 1980-2015 | + | + | + |  |  | + |
| Pre-labour rupture of membranes | ICD-9: 658.1; ICD-10: O42 | HMDC, MNS | 1980-2015 | + | + | + |  |  | + |
| Unspecified antepartum haemorrhage | ICD-9: 641.3-641.99, 640.8-640.9; ICD-10: O46, O20.8-O20.9 | HMDC, MNS | 1980-2015 | + | + | + |  |  | + |
| Threatened preterm labour | Threatened preterm labour <37 weeks | MNS | 1980-2015 | + | + | + |  |  | + |
| Uterine rupture | ICD-9: 665.0-665.1; ICD-10: O71.0-O71.1 | HMDC, MNS | 1980-2015 | + | + | + |  |  | + |
| Birth year | 5-year groups | MNS | 1980-2015 | + | + | + | + | + | + |
| Plurality | Singleton, Twin, Multiple (>2) gestation | MNS | 1980-2015 | + | + | + |  |  | + |
| Small-for-gestational age | <10th centile of birth weights for gestational week and sex | MNS | 1980-2015 | + | + | + |  |  |  |
| Congenital anomalies | Any birth defect | WARDA | 1980-2015 | + | + | + |  |  |  |
| **Past obstetric history** | | | | | | | | | |
| Gestational diabetes | ICD-9: 648.8; ICD-10: O24.4 | HMDC, MNS | 1980-2015 |  | + |  |  | + | + |
| Gestational hypertension | ICD-9: 642.3; ICD-10: O13 | HMDC, MNS | 1980-2015 |  | + |  |  | + | + |
| Urinary tract infection | ICD-9: 646.6, ICD-10: O23 | HMDC, MNS | 1980-2015 |  | + |  |  | + | + |
| Cancer registration | ICD-9: 140-239; ICD-10: C00-D49 | Cancer Registry, HMDC | 1980-2015 |  | + |  |  | + | + |
| Pre-eclampsia | ICD-9: 642.4, 642.5, 642.7; ICD-10: O11, O14 | HMDC, MNS | 1980-2015 |  | + |  |  | + | + |
| Threatened miscarriage | ICD-9: 640-640.03; ICD-10: O20.0 (<20 weeks’ gestation) | HMDC, MNS | 1980-2015 |  | + |  |  | + | + |
| Placenta praevia | ICD-9: 641.0-641.1, ICD-10: O44 | HMDC, MNS | 1980-2015 |  | + |  |  | + | + |
| Placental abruption | ICD-9: 641.2; ICD-10: O45 | HMDC, MNS | 1980-2015 |  | + |  |  | + | + |
| Pre-labour rupture of membranes | ICD-9: 658.1; ICD-10: O42 | HMDC, MNS | 1980-2015 |  | + |  |  | + | + |
| Unspecified antepartum haemorrhage | ICD-9: 641.3-641.99, 640.8-640.9; ICD-10: O46, O20.8-O20.9 | HMDC, MNS | 1980-2015 |  | + |  |  | + | + |
| Threatened preterm labour | Threatened preterm labour <37 weeks | HMDC, MNS | 1980-2015 |  | + |  |  | + | + |
| Uterine rupture | ICD-9: 665.0-665.1; ICD-10: O71.0-O71.1 | HMDC, MNS | 1980-2015 |  | + |  |  | + | + |
| Caesarean section delivery | Delivered by caesarean section | HMDC, MNS | 1998-2015 |  | + |  |  | + | + |
| Stillbirth | Stillbirth born after 28 weeks’ gestation | Death Registry | 1980-2015 |  | + |  |  | + | + |
| Gestational age of the last birth | <28, 28-31, 32-36, ≥37 weeks | MNS | 1980-2015 |  | + |  |  | + | + |
| Small-for-gestational age | <10th centile of birth weights for gestational week and sex | MNS | 1980-2015 |  | + |  |  | + | + |
| Congenital anomalies | Any birth defect | WARDA | 1980-2015 |  | + |  |  | + | + |
| **Parent's birth outcomes** | | | | | | | | | |
| Preterm birth | <37 weeks’ gestation | MNS | 1980-2015 |  |  | + |  |  |  |
| Small-for-gestational age | <10th centile of birth weights for gestational week and sex | MNS | 1980-2015 |  |  | + |  |  |  |
| Congenital anomalies | Any birth defect | WARDA | 1980-2015 |  |  | + |  |  |  |
| **Grandmothers' chronic medical conditions and obstetric history** | | | | | | | | | |
| Essential hypertension | ICD-9: 401; ICD-10: I10 | HMDC, MNS | 1980-2015 |  |  | + |  |  |  |
| Diabetes mellitus | ICD-9: 250; ICD-10: E08-E11, E13 | HMDC, MNS | 1980-2015 |  |  | + |  |  |  |
| Asthma | ICD-9: 493; ICD-10: J45 | HMDC, MNS | 1980-2015 |  |  | + |  |  |  |
| Miscarriage | ICD-9: 634; ICD-10: O03 (<20 weeks’ gestation) | HMDC, MNS | 1980-2015 |  |  | + |  |  |  |
| Circulatory system diseases | ICD-9: 390-459; ICD-10: 100-199 | HMDC, MNS | 1980-2015 |  |  | + |  |  |  |
| Gestational diabetes | ICD-9: 648.8; ICD-10: O24.4 | HMDC, MNS | 1980-2015 |  |  | + |  |  |  |
| Gestational hypertension | ICD-9: 642.3; ICD-10: O13 | HMDC, MNS | 1980-2015 |  |  | + |  |  |  |
| Urinary tract infection | ICD-9: 646.6, ICD-10: O23 | HMDC, MNS | 1980-2015 |  |  | + |  |  |  |
| Cancer registration | ICD-9: 140-239; ICD-10: C00-D49 | Cancer Registry, HMDC | 1980-2015 |  |  | + |  |  |  |
| Pre-eclampsia | ICD-9: 642.4, 642.5, 642.7; ICD-10: O11, O14 | HMDC, MNS | 1980-2015 |  |  | + |  |  |  |
| Threatened miscarriage | ICD-9: 640-640.03; ICD-10: O20.0 (<20 weeks’ gestation) | HMDC, MNS | 1980-2015 |  |  | + |  |  |  |
| Placenta praevia | ICD-9: 641.0-641.1, ICD-10: O44 | HMDC, MNS | 1980-2015 |  |  | + |  |  |  |
| Placental abruption | ICD-9: 641.2; ICD-10: O45 | HMDC, MNS | 1980-2015 |  |  | + |  |  |  |
| Pre-labour rupture of membranes | ICD-9: 658.1; ICD-10: O42 | HMDC, MNS | 1980-2015 |  |  | + |  |  |  |
| Unspecified antepartum haemorrhage | ICD-9: 641.3-641.99, 640.8-640.9; ICD-10: O46, O20.8-O20.9 | HMDC, MNS | 1980-2015 |  |  | + |  |  |  |
| Stillbirth | Stillbirth born after 28 weeks’ gestation | Death Registry | 1980-2015 |  |  | + |  |  |  |

ICD, International Classification of Diseases; MNS, Midwives Notifications System; HMDC, Hospital Morbidity Data Collection; WARDA, WA Register of Developmental Anomalies

Model A: Cohort - all births; Predictors - maternal socio-demographic factors, maternal chronic medical conditions, and current pregnancy characteristics and complications; Model B: Cohort - births of multiparous women; Predictors - Model A + maternal past obstetric history; Model C: Cohort - births of parents who were born during the study period; Predictors - Model A + parent’s birth outcomes and grandmother’s chronic medical conditions and obstetric history; Model D: Cohort - all births; Predictors - maternal socio-demographic factors, maternal chronic medical conditions, parity, and birth year; Model E: Cohort - births of multiparous women; Predictors - Model D + maternal past obstetric history; Model F: Cohort - births of multiparous women; Predictors - Model B excluding small-for-gestational age and congenital anomalies in current birth

**Supplementary Table 4. Hyperparameter search spaces and tuned values of classification algorithms, by models**

| **Classification algorithm** | **Hyperparameter** | **Hyperparameter values** | **Model** | | | | | |
| --- | --- | --- | --- | --- | --- | --- | --- | --- |
|  |  |  | **A** | **B** | **C** | **D** | **E** | **F** |
| **Logistic regression** | **C**: Inverse of L1 regularisation strength | 0.001, 0.003, 0.01, 0.03, 0.1, 0.3, 1 | 0.01 | 0.3 | 1 | 0.03 | 0.03 | 0.3 |
| **Decision tree** | **criterion**: Function to measure the split quality | gini, entropy | entropy | gini | entropy | entropy | entropy | entropy |
|  | **max_depth**: Maximum tree depth | 4, 6, 8, 10, 12, 14, 16 | 12 | 12 | 8 | 8 | 8 | 10 |
|  | **max_features**: Maximum number of predictors for best split | sqrt, 0.5, 0.75, 0.85, 1 | 0.75 | sqrt | 0.75 | 0.75 | 0.75 | 0.85 |
| **Random Forests** | **criterion**: Function to measure the split quality | gini, entropy | entropy | entropy | entropy | gini | entropy | entropy |
|  | **max_depth**: Maximum tree depth | 4, 6, 8, 10, 12, 14, 16 | 14 | 10 | 8 | 10 | 6 | 10 |
|  | **max_features**: Maximum number of predictors for best split | sqrt, 0.5, 0.75, 0.85, 1 | sqrt | sqrt | 0.75 | 1 | 0.85 | 0.5 |
|  | **n_estimators**: Number of trees in the forest | 50, 100, 300, 500, 700, 1000 | 300 | 300 | 300 | 300 | 300 | 300 |
| **Extreme gradient boosting** | **learning_rate**: Boosting learning rate | 0.001, 0.003, 0.01, 0.03, 0.1, 0.3, 1 | 0.03 | 0.1 | 0.03 | 0.1 | 0.1 | 0.03 |
|  | **max_depth**: Maximum tree depth for base learners | 2, 3, 4, 5, 6, 7, 8 | 5 | 2 | 5 | 2 | 2 | 3 |
|  | **n_estimators**: Number of gradient boosted trees | 50, 100, 300, 500, 700, 1000 | 300 | 300 | 300 | 300 | 300 | 300 |
|  | **reg_alpha**: L1 regularisation term on weights | 0.001, 0.003, 0.01, 0.03, 0.1, 0.3, 1 | 0.1 | 0.003 | 0.03 | 0.003 | 0.1 | 0.1 |
|  | **reg_lambda**: L2 regularisation term on weights | 0.001, 0.003, 0.01, 0.03, 0.1, 0.3, 1 | 0.001 | 0.01 | 1 | 0.01 | 0.003 | 1 |
|  | **subsample**: Subsample ratio of training instance | 0.3, 0.4, 0.5, 0.6, 0.7, 0.8 | 0.7 | 0.6 | 0.6 | 0.6 | 0.6 | 0.4 |
| **Multi-layer perceptron** | **activation**: Activation function for hidden layer | relu, logistic, tanh | relu | tanh | tanh | tanh | tanh | tanh |
|  | **alpha**: L2 regularisation term on weights | 0.001, 0.003, 0.01, 0.03, 0.1, 0.3, 1 | 0.03 | 0.03 | 0.03 | 0.003 | 0.003 | 0.003 |
|  | **batch_size**: Size of minibatches for stochastic optimisers | 200, 500, 1500, 3000 | 500 | 1500 | 1500 | 200 | 200 | 200 |
|  | **hidden_layer_size_0**: Number of neurons (1^st^ hidden layer) | Number of predictors, 100, 200, 250 | 200 | 100 | 100 | 38 | 73 | 88 |
|  | **hidden_layer_size_1**: Number of neurons (2^nd^ hidden layer) | 0.5* no. of predictors, 50, 100, 125 | 28 | - | - | 50 | 36 | 50 |
|  | **learning_rate_init**: Step-size in updating weights | 0.0001, 0.0003, 0.001, 0.003, 0.01, 0.03, 0.1, 0.3, 1 | 0.0003 | 0.01 | 0.01 | 0.03 | 0.03 | 0.03 |

Model A: Cohort - all births; Predictors - maternal socio-demographic factors, maternal chronic medical conditions, and current pregnancy characteristics and complications; Model B: Cohort - births of multiparous women; Predictors - Model A + maternal past obstetric history; Model C: Cohort - births of parents who were born during the study period; Predictors - Model A + parent’s birth outcomes and grandmother’s chronic medical conditions and obstetric history; Model D: Cohort - all births; Predictors - maternal socio-demographic factors, maternal chronic medical conditions, parity, and birth year; Model E: Cohort - births of multiparous women; Predictors - Model D + maternal past obstetric history; Model F: Cohort - births of multiparous women; Predictors - Model B excluding small-for-gestational age and congenital anomalies in current birth

**Supplementary Table 5. Feature importance of models A, B, and C, by classification algorithms**

|  | **Model A** | | | | **Model B** | | | | **Model C** | | | |
| --- | --- | --- | --- | --- | --- | --- | --- | --- | --- | --- | --- | --- |
| **Rank** | **Logistic regression** | **Decision tree** | **Random Forests** | **XGBoost** | **Logistic regression** | **Decision tree** | **Random Forests** | **XGBoost** | **Logistic regression** | **Decision tree** | **Random Forests** | **XGBoost** |
|  | Feature  Mean^ (standard error) | | | | Feature  Mean^ (standard error) | | | | Feature  Mean^ (standard error) | | | |
| 1 | Multiple (>2) gestation | Pre-labour rupture of membranes | Pre-labour rupture of membranes | Pre-labour rupture of membranes | Multiple (>2) gestation | Pre-labour rupture of membranes | Pre-labour rupture of membranes | Pre-labour rupture of membranes | Multiple (>2) gestation | Singleton | Pre-labour rupture of membranes | Threatened preterm labour |
|  | 3.765 (0.004) | 0.256 (0.001) | 0.238 (0.001) | 0.199 (0.005) | 5.592 (0.022) | 0.236 (0.002) | 0.235 (<0.001) | 0.137 (0.004) | 5.435 (0.019) | 0.239 (0.003) | 0.208 (0.001) | 0.154 (0.003) |
| 2 | Twin | Singleton | Threatened miscarriage | Singleton | Twin | Singleton | Singleton | *Previous GA ≥37w* | Twin | Pre-labour rupture of membranes | Threatened preterm labour | Singleton |
|  | 2.704 (0.002) | 0.216 (0.015) | 0.136 (0.001) | 0.179 (0.005) | 2.815 (0.001) | 0.176 (0.02) | 0.182 (0.001) | 0.113 (0.004) | 3.025 (0.008) | 0.209 (0.004) | 0.202 (0.002) | 0.130 (0.004) |
| 3 | Threatened preterm labour | Threatened miscarriage | Singleton | Threatened miscarriage | Placental abruption | *Previous GA ≥37w* | *Previous GA ≥37w* | Singleton | Threatened preterm labour | Threatened preterm labour | Singleton | Pre-labour rupture of membranes |
|  | 2.491 (0.002) | 0.135 (0.006) | 0.131 (0.002) | 0.087 (0.003) | 2.389 (0.007) | 0.158 (0.005) | 0.153 (0.001) | 0.111 (0.007) | 2.545 (0.004) | 0.196 (0.006) | 0.191 (0.002) | 0.103 (0.002) |
| 4 | Placental abruption | Pre-eclampsia | Twin | Threatened preterm labour | Pre-labour rupture of membranes | Threatened miscarriage | Threatened miscarriage | Twin | Placental abruption | Pre-eclampsia | Pre-eclampsia | Twin |
|  | 2.318 (0.003) | 0.085 (0.001) | 0.106 (0.002) | 0.073 (0.001) | 2.274 (0.002) | 0.114 (0.011) | 0.107 (<0.001) | 0.105 (0.022) | 2.416 (0.007) | 0.131 (0.003) | 0.123 (0.001) | 0.092 (0.006) |
| 5 | Pre-labour rupture of membranes | Threatened preterm labour | Threatened preterm labour | Twin | Threatened preterm labour | Threatened preterm labour | Threatened preterm labour | Threatened miscarriage | Pre-eclampsia | Threatened miscarriage | Threatened miscarriage | Pre-eclampsia |
|  | 2.249 (0.002) | 0.082 (0.001) | 0.078 (<0.001) | 0.064 (0.005) | 2.217 (0.003) | 0.062 (0.001) | 0.065 (<0.001) | 0.070 (0.003) | 2.005 (0.006) | 0.069 (0.003) | 0.070 (<0.001) | 0.079 (0.001) |
| 6 | Placenta praevia | Placental abruption | Pre-eclampsia | Placental abruption | Placenta praevia | Pre-eclampsia | Pre-eclampsia | Threatened preterm labour | Pre-labour rupture of membranes | Placental abruption | Twin | Threatened miscarriage |
|  | 1.735 (0.003) | 0.046 (0.001) | 0.072 (<0.001) | 0.062 (0.001) | 1.976 (0.003) | 0.041 (<0.001) | 0.044 (<0.001) | 0.054 (0.002) | 1.949 (0.004) | 0.042 (0.001) | 0.038 (0.001) | 0.046 (0.001) |
| 7 | Pre-eclampsia | Unspecified antepartum haemorrhage | Placental abruption | Pre-eclampsia | *Previous GA 20-31w* | Placental abruption | Placental abruption | Placental abruption | Diabetes | Congenital anomalies | Placental abruption | Placental abruption |
|  | 1.552 (0.001) | 0.028 (0.002) | 0.039 (<0.001) | 0.060 (0.001) | 1.653 (0.007) | 0.039 (0.001) | 0.043 (<0.001) | 0.034 (0.001) | 1.904 (0.012) | 0.025 (0.003) | 0.037 (<0.001) | 0.044 (0.001) |
| 8 | Diabetes | Placenta praevia | Unspecified antepartum haemorrhage | Placenta praevia | *Previous GA 32-36w* | Placenta praevia | Placenta praevia | Pre-eclampsia | Placenta praevia | Unspecified antepartum haemorrhage | Unspecified antepartum haemorrhage | Unspecified antepartum haemorrhage |
|  | 1.394 (0.005) | 0.027 (<0.001) | 0.033 (<0.001) | 0.051 (0.001) | 1.471 (0.001) | 0.035 (0.001) | 0.036 (<0.001) | 0.032 (0.001) | 1.766 (0.009) | 0.024 (0.004) | 0.028 (0.001) | 0.035 (0.001) |
| 9 | Threatened miscarriage | Congenital anomalies | Congenital anomalies | Unspecified antepartum haemorrhage | *Uterine rupture in last birth* | Unspecified antepartum haemorrhage | Twin | Unspecified antepartum haemorrhage | Uterine rupture | Caucasian | Congenital anomalies | Placenta praevia |
|  | 1.088 (0.002) | 0.021 (<0.001) | 0.022 (<0.001) | 0.034 (<0.001) | 1.393 (0.041) | 0.021 (0.001) | 0.025 (0.001) | 0.028 (0.001) | 1.590 (0.125) | 0.021 (0.002) | 0.025 (<0.001) | 0.032 (0.001) |
| 10 | Unspecified antepartum haemorrhage | Indigenous | Placenta praevia | Indigenous | Pre-eclampsia | Twin | Unspecified antepartum haemorrhage | Placenta praevia | Unspecified antepartum haemorrhage | Placenta praevia | Caucasian | Congenital anomalies |
|  | 1.012 (0.002) | 0.014 (0.002) | 0.021 (<0.001) | 0.032 (<0.001) | 1.366 (0.003) | 0.019 (0.019) | 0.021 (<0.001) | 0.028 (0.001) | 1.065 (0.006) | 0.007 (0.002) | 0.017 (0.001) | 0.032 (0.001) |

Emphasis: none, current pregnancy characteristics and complications; underline, maternal socio-demographic factors or chronic medical conditions; italic, past obstetric history

^ Mean of non-standardised absolute beta coefficient for logistic regression or mean of impurity-based importance measure for tree-based classifiers (decision tree, Random Forest, XGBoost)

Model A: Cohort - all births; Predictors - maternal socio-demographic factors, maternal chronic medical conditions, and current pregnancy characteristics and complications; Model B: Cohort - births of multiparous women; Predictors - Model A + maternal past obstetric history; Model C: Cohort - births of parents who were born during the study period; Predictors - Model A + parent’s birth outcomes and grandmother’s chronic medical conditions and obstetric history

**Supplementary Table 6. Feature importance of models D, E and F, by classification algorithms**

|  | **Model D** | | | | **Model E** | | | | **Model F** | | | |
| --- | --- | --- | --- | --- | --- | --- | --- | --- | --- | --- | --- | --- |
| **Rank** | **Logistic regression** | **Decision tree** | **Random Forests** | **XGBoost** | **Logistic regression** | **Decision tree** | **Random Forests** | **XGBoost** | **Logistic regression** | **Decision tree** | **Random Forests** | **XGBoost** |
|  | Feature  Mean^ (standard error) | | | | Feature  Mean^ (standard error) | | | | Feature  Mean^ (standard error) | | | |
| 1 | Diabetes | Indigenous | Indigenous | Indigenous | *Previous GA 20-31w* | *Previous GA ≥37w* | *Previous GA ≥37w* | *Previous GA ≥37w* | Multiple (>2) gestation | Pre-labour rupture of membranes | Pre-labour rupture of membranes | Pre-labour rupture of membranes |
|  | 1.46 (0.004) | 0.278 (0.031) | 0.13 (0.002) | 0.205 (0.008) | 1.475 (0.006) | 0.469 (0.068) | 0.613 (0.003) | 0.295 (0.015) | 5.518 (0.021) | 0.241 (0.003) | 0.236 (<0.001) | 0.136 (0.004) |
| 2 | Indigenous | Diabetes | Caucasian | Diabetes | *Previous GA <20w* | *Previous GA 32-36w* | Indigenous | *Previous GA 32-36w* | Twin | Singleton | Singleton | Singleton |
|  | 0.659 (0.002) | 0.136 (0.001) | 0.088 (0.002) | 0.09 (0.002) | 1.345 (0.004) | 0.166 (0.067) | 0.086 (0.001) | 0.093 (0.014) | 2.768 (0.004) | 0.193 (0.021) | 0.146 (0.001) | 0.108 (0.003) |
| 3 | Maternal age*≥*40y | Parity=1 | Diabetes | Parity=1 | *Previous GA 32-36w* | Indigenous | *Previous GA 32-36w* | Indigenous | Placental abruption | *Previous GA ≥37w* | *Previous GA ≥37w* | *Previous GA ≥37w* |
|  | 0.508 (0.003) | 0.108 (0.001) | 0.07 (0.002) | 0.079 (0.005) | 1.284 (0.003) | 0.071 (0.01) | 0.068 (0.003) | 0.068 (0.003) | 2.358 (0.006) | 0.167 (0.003) | 0.128 (0.001) | 0.103 (0.003) |
| 4 | Hypertension | Smoking | Parity=1 | Smoking | Diabetes | *Caesarean delivery in last birth* | *Caesarean delivery in last birth* | *Caesarean delivery in last birth* | Pre-labour rupture of membranes | Threatened miscarriage | Threatened miscarriage | Threatened miscarriage |
|  | 0.495 (0.006) | 0.077 (0.001) | 0.061 (0.001) | 0.069 (0.002) | 0.957 (0.005) | 0.064 (0.001) | 0.066 (0.001) | 0.037 (0.001) | 2.261 (0.002) | 0.107 (0.002) | 0.124 (<0.001) | 0.088 (0.002) |
| 5 | Parity=0 | Parity=2 | Smoking | Caucasian | *Threatened preterm labour in last birth* | Smoking | Smoking | Smoking | Threatened preterm labour | Threatened preterm labour | Threatened preterm labour | Threatened preterm labour |
|  | 0.302 (0.001) | 0.064 (0.002) | 0.059 (0.001) | 0.049 (0.006) | 0.498 (0.004) | 0.032 (0.002) | 0.033 (<0.001) | 0.035 (0.002) | 2.203 (0.004) | 0.069 (<0.001) | 0.069 (<0.001) | 0.062 (0.001) |
| 6 | Circulatory system diseases | Maternal age 35-39y | Parity=0 | Maternal age*≥*40y | Indigenous | *Small-for-gestational age in last birth* | *Small-for-gestational age in last birth* | *Threatened miscarriage in last birth* | Placenta praevia | Placental abruption | Twin | Twin |
|  | 0.299 (0.002) | 0.048 (0.004) | 0.053 (0.001) | 0.043 (0.001) | 0.471 (0.003) | 0.027 (0.001) | 0.023 (0.001) | 0.033 (0.002) | 1.966 (0.005) | 0.045 (0.001) | 0.064 (0.001) | 0.055 (0.004) |
| 7 | Maternal age 35-39y | Maternal age*≥*40y | Parity>=3 | Maternal age 35-39y | *Threatened preterm labour in earlier birth* | *Pre-labour rupture of membranes in last birth* | Parity>=3 | Parity>=3 | *Previous GA 20-31w* | Pre-eclampsia | Pre-eclampsia | *Previous GA 32-36w* |
|  | 0.275 (0.001) | 0.047 (0.004) | 0.046 (0.001) | 0.04 (0.001) | 0.457 (0.004) | 0.024 (0.011) | 0.021 (<0.001) | 0.029 (0.002) | 1.625 (0.007) | 0.044 (<0.001) | 0.044 (<0.001) | 0.044 (0.005) |
| 8 | Miscarriage | Maternal age 25-29y | IRSD <20% | Parity=0 | Maternal age <20y | Parity>=3 | Diabetes | Caucasian | *Uterine rupture in last birth* | Placenta praevia | Placental abruption | Pre-eclampsia |
|  | 0.263 (0.002) | 0.027 (0.003) | 0.029 (0.001) | 0.037 (0.001) | 0.402 (0.005) | 0.024 (0.003) | 0.011 (<0.001) | 0.029 (0.001) | 1.464 (0.093) | 0.037 (<0.001) | 0.04 (<0.001) | 0.042 (0.001) |
| 9 | Smoking | Parity>=3 | Maternal age 25-29y | Parity>=3 | *Caesarean delivery in last birth* | *Pre-eclampsia in last birth* | *Pre-eclampsia in last birth* | *Pre-labour rupture of membranes in last birth* | *Previous GA 32-36w* | Twin | Placenta praevia | Placental abruption |
|  | 0.261 (0.001) | 0.021 (0.001) | 0.029 (<0.001) | 0.03 (0.002) | 0.384 (0.002) | 0.016 (0.001) | 0.011 (<0.001) | 0.024 (0.002) | 1.449 (0.001) | 0.022 (0.02) | 0.033 (<0.001) | 0.039 (0.001) |
| 10 | Asthma | Other ethnicities | Maternal age <20y | Asthma | *Pre-labour rupture of membranes in last birth* | Caucasian | *Pre-labour rupture of membranes in last birth* | *Small-for-gestational age in last birth* | Pre-eclampsia | Unspecified antepartum haemorrhage | *Previous GA 32-36w* | Placenta praevia |
|  | 0.236 (0.001) | 0.021 (0.016) | 0.028 (0.001) | 0.027 (0.001) | 0.365 (0.002) | 0.015 (0.009) | 0.011 (<0.001) | 0.023 (0.001) | 1.344 (0.002) | 0.02 (0.002) | 0.028 (0.001) | 0.038 (0.001) |

Emphasis: none, current pregnancy characteristics and complications; underline, maternal socio-demographic factors or chronic medical conditions; italic, past obstetric history

^ Mean of non-standardised absolute beta coefficient for logistic regression or mean of impurity-based importance measure for tree-based classifiers (decision tree, Random Forest, XGBoost)

Model D: Cohort - all births; Predictors - maternal socio-demographic factors, maternal chronic medical conditions, parity, and birth year; Model E: Cohort - births of multiparous women; Predictors - Model D + maternal past obstetric history; Model F: Cohort - births of multiparous women; Predictors - Model B excluding small-for-gestational age and congenital anomalies in current birth

**Supplementary Table 7. Estimated coefficients of models A, B and C produced using regularised logistic regression algorithms**

|  |  | **Model A (n=953,244)** | | **Model B (n=465,037)** | | **Model C (n=135,943)** | |
| --- | --- | --- | --- | --- | --- | --- | --- |
|  |  | Preterm birth n (%) | β (95% CI),  *P*-value | Preterm birth n (%) | β (95% CI),  *P*-value | Preterm birth n (%) | β (95% CI),  *P*-value |
| Intercept |  | 81,578 (8.6) | -1.40 (-1.42,-1.37), <0.01 | 37,978 (8.2) | -1.56 (-1.59,-1.53), <0.01 | 12,895 (9.5) | -1.42 (-1.47,-1.36), <0.01 |
| **Maternal socio-demographic factors** | | | | | | | |
| Age, years | <20 | 5,736 (11.0) | 0.23 (0.21,0.26), <0.01 | 1,201 (14.4) | 0.52 (0.47,0.58), <0.01 | 2,176 (11.1) | 0.12 (0.08,0.17), <0.01 |
|  | 20-24 | 14,968 (8.3) | Reference | 6,376 (9.0) | Reference | 4,237 (9.6) | Reference |
|  | 25-29 | 23,788 (7.8) | -0.07 (-0.09,-0.06), <0.01 | 10,879 (7.5) | -0.18 (-0.20,-0.15), <0.01 | 4,105 (8.8) | -0.11 (-0.14,-0.07), <0.01 |
|  | 30-34 | 22,681 (8.3) | -0.04 (-0.05,-0.02), <0.01 | 11,594 (7.6) | -0.17 (-0.20,-0.15), <0.01 | 2,131 (9.2) | -0.10 (-0.15,-0.05), <0.01 |
|  | 35-39 | 11,655 (9.7) | 0.06 (0.04,0.08), <0.01 | 6,443 (8.8) | -0.10 (-0.13,-0.07), <0.01 | 211 (10.4) | -0.13 (-0.25,-0.02), 0.02 |
|  | ≥40 | 2,750 (12.3) | 0.21 (0.18,0.25), <0.01 | 1,485 (11.2) | 0.09 (0.04,0.13), <0.01 | 35 (16.0) | 0.11 (-0.21,0.44), 0.49 |
| Ethnicity | Caucasian | 64,214 (8.1) | Reference | 29,171 (7.5) | Reference | 9,467 (8.5) | Reference |
|  | Indigenous | 7,520 (15.4) | 0.65 (0.62,0.67), <0.01 | 4,881 (15.6) | 0.54 (0.51,0.57), <0.01 | 2,043 (15.3) | 0.48 (0.43,0.53), <0.01 |
|  | Other | 9,844 (8.9) | 0.13 (0.11,0.15), <0.01 | 3,926 (9.0) | 0.22 (0.20,0.25), <0.01 | 1,385 (12.2) | 0.36 (0.31,0.41), <0.01 |
| Socio-economic status, IRSD percentile | <20 | 18,136 (9.8) | 0.05 (0.03,0.07), <0.01 | 9,881 (10.1) | 0.03 (0.00,0.06), 0.03 | 3,546 (11.2) | 0.09 (0.05,0.14), <0.01 |
|  | 20-39 | 15,724 (8.6) | 0.02 (0.00,0.03), 0.08 | 7,533 (8.3) | -0.01 (-0.04,0.01), 0.25 | 2,594 (9.8) | 0.04 (-0.01,0.09), 0.10 |
|  | 40-59 | 14,804 (8.1) | 0.00 (-0.02,0.01), 0.71 | 6,684 (7.7) | -0.02 (-0.05,0.00), 0.07 | 2,368 (9.1) | 0.02 (-0.03,0.06), 0.47 |
|  | 60-79 | 14,847 (8.2) | 0.01 (-0.01,0.03), 0.24 | 6,542 (7.5) | 0.01 (-0.01,0.04), 0.38 | 2,330 (8.4) | 0.00 (-0.05,0.05), 1.00 |
|  | ≥80 | 14,261 (8.0) | Reference | 6,264 (7.3) | Reference | 1,710 (8.3) | Reference |
|  | Unknown | 3,806 (8.3) | 0.00 (-0.03,0.03), 1.00 | 1,074 (6.5) | -0.12 (-0.18,-0.07), <0.01 | 347 (9.8) | -0.08 (-0.18,0.03), 0.15 |
| Remoteness of residence | Major cities | 54,014 (8.6) | Reference | 24,018 (8.1) | Reference | 7,809 (9.4) | Reference |
|  | Regional/Remote | 19,288 (8.6) | -0.06 (-0.08,-0.05), <0.01 | 10,225 (8.3) | -0.09 (-0.11,-0.07), <0.01 | 4,057 (9.6) | -0.14 (-0.17,-0.11), <0.01 |
|  | Unknown | 8,276 (8.6) | 0.01 (-0.02,0.03), 0.58 | 3,735 (8.0) | -0.02 (-0.05,0.01), 0.31 | 1,029 (10.0) | 0.05 (-0.01,0.11), 0.13 |
| Smoking | Yes | 9,713 (11.6) | 0.24 (0.22,0.26), <0.01 | 6,109 (12.2) | 0.20 (0.18,0.23), <0.01 | 3,729 (12.5) | 0.30 (0.26,0.33), <0.01 |
|  | No | 38,002 (8.5) | Reference | 17,500 (7.9) | Reference | 9,166 (8.6) | Reference |
|  | Unknown | 33,863 (8.0) | 0.03 (0.00,0.05), 0.06 | 14,369 (7.4) | 0.09 (0.05,0.13), <0.01 | N/A |  |
| **Maternal chronic medical conditions** | | | | | | | |
| Essential hypertension | Yes | 531 (17.0) | 0.32 (0.23,0.41), <0.01 | 510 (16.8) | 0.19 (0.10,0.29), <0.01 | 92 (14.6) | 0.04 (-0.16,0.25), 0.67 |
|  | No | 81,047 (8.5) | Reference | 37,468 (8.1) | Reference | 12,803 (9.5) | Reference |
| Diabetes mellitus | Yes | 1,044 (33.2) | 1.39 (1.30,1.49), <0.01 | 769 (31.4) | 1.14 (1.03,1.25), <0.01 | 233 (39.5) | 1.83 (1.60,2.06), <0.01 |
|  | No | 80,534 (8.5) | Reference | 37,209 (8.0) | Reference | 12,662 (9.4) | Reference |
| Asthma | Yes | 4,546 (10.8) | 0.13 (0.10,0.15), <0.01 | 4,220 (10.7) | 0.08 (0.06,0.11), <0.01 | 1,234 (10.8) | 0.01 (-0.04,0.06), 0.70 |
|  | No | 77,032 (8.5) | Reference | 33,758 (7.9) | Reference | 11,661 (9.4) | Reference |
| Miscarriage | Yes | 3,055 (11.5) | 0.09 (0.06,0.12), <0.01 | 2,999 (11.4) | 0.06 (0.02,0.09), <0.01 | 368 (14.3) | 0.08 (-0.02,0.18), 0.12 |
|  | No | 78,523 (8.5) | Reference | 34,979 (8.0) | Reference | 12,527 (9.4) | Reference |
| Obesity | Yes | 794 (12.6) | -0.01 (-0.07,0.06), 0.82 | 624 (13.3) | -0.01 (-0.09,0.06), 0.72 | 200 (9.7) | -0.45 (-0.56,-0.33), <0.01 |
|  | No | 80,784 (8.5) | Reference | 37,354 (8.1) | Reference | 12,695 (9.5) | Reference |
| Circulatory system diseases | Yes | 1,168 (13.2) | 0.07 (0.02,0.12), <0.01 | 1,007 (13.4) | 0.14 (0.08,0.20), <0.01 | 66 (21.4) | 0.19 (-0.09,0.47), 0.17 |
|  | No | 80,410 (8.5) | Reference | 36,971 (8.1) | Reference | 12,829 (9.5) | Reference |
| **Current pregnancy characteristics and complications** | | | | | | | |
| Parity | 0 | 35,777 (9.3) | 0.08 (0.07,0.09), <0.01 | N/A |  | 6,541 (9.4) | -0.02 (-0.05,0.02), 0.29 |
|  | 1 | 23,772 (7.4) | Reference | 19,476 (7.4) | Reference | 3,510 (8.4) | Reference |
|  | 2 | 12,072 (7.8) | 0.02 (0.01,0.04), <0.01 | 9,901 (7.9) | 0.00 (-0.02,0.02), 0.94 | 1,649 (10.2) | 0.12 (0.08,0.17), <0.01 |
|  | ≥3 | 9,957 (10.9) | 0.21 (0.19,0.23), <0.01 | 8,601 (11.2) | 0.16 (0.13,0.18), <0.01 | 1,195 (14.9) | 0.45 (0.39,0.51), <0.01 |
| Gestational diabetes | Yes | 5,956 (13.9) | 0.36 (0.34,0.39), <0.01 | 2,944 (13.7) | 0.29 (0.25,0.32), <0.01 | 1,357 (15.0) | 0.32 (0.27,0.37), <0.01 |
|  | No | 75,622 (8.3) | Reference | 35,034 (7.9) | Reference | 11,538 (9.1) | Reference |
| Gestational hypertension | Yes | 2,170 (10.2) | -0.31 (-0.34,-0.27), <0.01 | 1,106 (12.6) | -0.24 (-0.30,-0.18), <0.01 | 452 (8.7) | -0.58 (-0.65,-0.50), <0.01 |
|  | No | 79,408 (8.5) | Reference | 36,872 (8.1) | Reference | 12,443 (9.5) | Reference |
| Urinary tract infection | Yes | 7,234 (14.3) | 0.20 (0.18,0.22), <0.01 | 3,848 (15.2) | 0.18 (0.15,0.21), <0.01 | 1,133 (14.0) | 0.08 (0.02,0.14), <0.01 |
|  | No | 74,344 (8.2) | Reference | 34,130 (7.8) | Reference | 11,762 (9.2) | Reference |
| Cancer registration | Yes | 365 (12.7) | 0.43 (0.34,0.51), <0.01 | 181 (11.4) | 0.57 (0.45,0.69), <0.01 | 41 (12.1) | 0.64 (0.39,0.89), <0.01 |
|  | No | 81,213 (8.5) | Reference | 37,797 (8.2) | Reference | 12,854 (9.5) | Reference |
| Pre-eclampsia | Yes | 12,932 (21.2) | 1.55 (1.53,1.57), <0.01 | 4,248 (21.9) | 1.38 (1.35,1.42), <0.01 | 1,803 (28.2) | 2.01 (1.94,2.07), <0.01 |
|  | No | 68,646 (7.7) | Reference | 33,730 (7.6) | Reference | 11,092 (8.6) | Reference |
| Threatened miscarriage | Yes | 36,658 (19.0) | 1.08 (1.07,1.10), <0.01 | 17,176 (18.2) | 0.99 (0.97,1.00), <0.01 | 4,652 (19.6) | 0.80 (0.77,0.84), <0.01 |
|  | No | 44,920 (5.9) | Reference | 20,802 (5.6) | Reference | 8,243 (7.3) | Reference |
| Placenta praevia | Yes | 2,954 (30.2) | 1.73 (1.68,1.78), <0.01 | 1,728 (33.2) | 1.98 (1.90,2.05), <0.01 | 329 (32.3) | 1.75 (1.58,1.91), <0.01 |
|  | No | 78,624 (8.3) | Reference | 36,250 (7.9) | Reference | 12,566 (9.3) | Reference |
| Placental abruption | Yes | 3,846 (48.4) | 2.32 (2.25,2.39), <0.01 | 1,951 (49.9) | 2.38 (2.27,2.49), <0.01 | 496 (53.1) | 2.41 (2.19,2.63), <0.01 |
|  | No | 77,732 (8.2) | Reference | 36,027 (7.8) | Reference | 12,399 (9.2) | Reference |
| Pre-labour rupture of membranes | Yes | 23,157 (35.5) | 2.25 (2.23,2.27), <0.01 | 9,885 (38.0) | 2.27 (2.24,2.31), <0.01 | 3,756 (29.5) | 1.94 (1.89,1.98), <0.01 |
|  | No | 58,421 (6.6) | Reference | 28,093 (6.4) | Reference | 9,139 (7.4) | Reference |
| Unspecified antepartum haemorrhage | Yes | 8,173 (24.4) | 1.01 (0.99,1.04), <0.01 | 4,494 (25.4) | 0.98 (0.94,1.02), <0.01 | 1,336 (26.6) | 1.08 (1.01,1.16), <0.01 |
|  | No | 73,405 (8.0) | Reference | 33,484 (7.5) | Reference | 11,559 (8.8) | Reference |
| Threatened preterm labour | Yes | 7,390 (52.3) | 2.48 (2.42,2.54), <0.01 | 3,954 (48.9) | 2.21 (2.13,2.28), <0.01 | 2,407 (53.8) | 2.56 (2.45,2.67), <0.01 |
|  | No | 74,188 (7.9) | Reference | 34,024 (7.4) | Reference | 10,488 (8.0) | Reference |
| Uterine rupture | Yes | 30 (30.0) | 0.00 (-0.49,0.49), 1.00 | 19 (26.4) | 1.21 (0.62,1.79), <0.01 | <5 | 1.67 (-0.10,3.45), 0.06 |
|  | No | 81,548 (8.6) | Reference | 37,959 (8.2) | Reference | 12,893 (9.5) | Reference |
| Birth year | 1980-1984 | 8,077 (7.5) | -0.03 (-0.06,0.01), 0.11 | 1,918 (7.3) | -0.05 (-0.10,0.00), 0.06 | N/A |  |
|  | 1985-1989 | 9,648 (7.9) | 0.00 (-0.03,0.04), 0.87 | 4,405 (7.1) | -0.09 (-0.14,-0.04), <0.01 | N/A |  |
|  | 1990-1994 | 10,316 (8.2) | -0.03 (-0.07,0.00), 0.04 | 5,129 (7.5) | -0.13 (-0.18,-0.09), <0.01 | N/A |  |
|  | 1995-1999 | 10,844 (8.5) | -0.10 (-0.12,-0.08), <0.01 | 5,437 (7.9) | -0.17 (-0.20,-0.13), <0.01 | 190 (11.9) | 0.01 (-0.12,0.13), 0.92 |
|  | 2000-2004 | 11,284 (9.1) | -0.18 (-0.20,-0.16), <0.01 | 5,804 (8.7) | -0.20 (-0.22,-0.17), <0.01 | 1,293 (10.0) | -0.22 (-0.27,-0.17), <0.01 |
|  | 2005-2009 | 13,450 (9.2) | -0.11 (-0.13,-0.10), <0.01 | 6,659 (8.8) | -0.17 (-0.19,-0.14), <0.01 | 3,580 (9.6) | -0.14 (-0.18,-0.11), <0.01 |
|  | 2010-2015 | 17,959 (9.1) | Reference | 8,626 (8.8) | Reference | 7,832 (9.3) | Reference |
| Plurality | Singleton | 66,082 (7.1) | Reference | 30,867 (6.8) | Reference | 10,755 (8.1) | Reference |
|  | Twin | 14,587 (57.5) | 2.70 (2.65,2.75), <0.01 | 6,735 (53.1) | 2.82 (2.75,2.88), <0.01 | 2,082 (66.2) | 3.04 (2.88,3.20), <0.01 |
|  | Multiple (>2) | 909 (97.7) | 3.78 (3.32,4.24), <0.01 | 376 (96.9) | 5.62 (4.08,7.17), <0.01 | 58 (100.0) | 5.45 (0.93,9.97), 0.02 |
| Small-for-gestational age | Yes | 8,399 (9.2) | -0.10 (-0.12,-0.09), <0.01 | 3,329 (9.6) | -0.18 (-0.21,-0.16), <0.01 | 1,305 (10.7) | 0.00 (-0.05,0.05), 0.95 |
|  | No | 73,179 (8.5) | Reference | 34,649 (8.0) | Reference | 11,590 (9.4) | Reference |
| Congenital anomalies | Yes | 8,831 (17.0) | 0.87 (0.85,0.89), <0.01 | 4,013 (16.3) | 0.94 (0.91,0.97), <0.01 | 1,421 (20.1) | 1.02 (0.96,1.08), <0.01 |
|  | No | 72,747 (8.1) | Reference | 33,965 (7.7) | Reference | 11,474 (8.9) | Reference |
| **Past obstetric history** | | | | | | | |
| Gestational diabetes | No |  |  | 36,207 (8.0) | Reference |  |  |
|  | Yes, in an earlier birth |  |  | 276 (12.7) | -0.08 (-0.19,0.03), 0.15 |  |  |
|  | Yes, in last birth |  |  | 1,495 (12.1) | 0.10 (0.05,0.14), <0.01 |  |  |
| Gestational hypertension | No |  |  | 36,378 (8.1) | Reference |  |  |
|  | Yes, in an earlier birth |  |  | 547 (14.1) | 0.04 (-0.05,0.12), 0.41 |  |  |
|  | Yes, in last birth |  |  | 1,053 (10.0) | 0.08 (0.03,0.13), <0.01 |  |  |
| Urinary tract infection | No |  |  | 32,512 (7.7) | Reference |  |  |
|  | Yes, in an earlier birth |  |  | 2,042 (13.2) | 0.01 (-0.03,0.05), 0.58 |  |  |
|  | Yes, in last birth |  |  | 3,424 (12.1) | 0.03 (0.00,0.06), 0.06 |  |  |
| Cancer registration | No |  |  | 37,828 (8.2) | Reference |  |  |
|  | Yes, in an earlier birth |  |  | 48 (13.0) | -0.08 (-0.34,0.18), 0.54 |  |  |
|  | Yes, in last birth |  |  | 102 (9.6) | 0.07 (-0.08,0.23), 0.34 |  |  |
| Pre-eclampsia | No |  |  | 32,086 (7.7) | Reference |  |  |
|  | Yes, in an earlier birth |  |  | 1,638 (11.1) | 0.08 (0.04,0.12), <0.01 |  |  |
|  | Yes, in last birth |  |  | 4,254 (12.4) | 0.03 (0.00,0.06), 0.03 |  |  |
| Threatened miscarriage | No |  |  | 22,992 (6.9) | Reference |  |  |
|  | Yes, in an earlier birth |  |  | 3,758 (11.3) | 0.17 (0.14,0.20), <0.01 |  |  |
|  | Yes, in last birth |  |  | 11,228 (11.7) | 0.09 (0.07,0.10), <0.01 |  |  |
| Placenta praevia | No |  |  | 37,242 (8.1) | Reference |  |  |
|  | Yes, in an earlier birth |  |  | 225 (15.3) | 0.04 (-0.09,0.17), 0.57 |  |  |
|  | Yes, in last birth |  |  | 511 (13.0) | -0.19 (-0.27,-0.11), <0.01 |  |  |
| Placental abruption | No |  |  | 36,652 (8.0) | Reference |  |  |
|  | Yes, in an earlier birth |  |  | 435 (19.3) | 0.31 (0.20,0.41), <0.01 |  |  |
|  | Yes, in last birth |  |  | 891 (21.4) | 0.20 (0.12,0.28), <0.01 |  |  |
| Pre-labour rupture of membranes | No |  |  | 30,850 (7.4) | Reference |  |  |
|  | Yes, in an earlier birth |  |  | 1,974 (14.3) | 0.24 (0.20,0.29), <0.01 |  |  |
|  | Yes, in last birth |  |  | 5,154 (15.7) | 0.00 (-0.03,0.03), 0.90 |  |  |
| Unspecified antepartum haemorrhage | No |  |  | 34,261 (7.8) | Reference |  |  |
|  | Yes, in an earlier birth |  |  | 1,257 (15.8) | 0.19 (0.13,0.25), <0.01 |  |  |
|  | Yes, in last birth |  |  | 2,460 (15.1) | 0.15 (0.11,0.19), <0.01 |  |  |
| Threatened preterm labour | No |  |  | 36,138 (7.9) | Reference |  |  |
|  | Yes, in an earlier birth |  |  | 495 (20.9) | 0.49 (0.39,0.60), <0.01 |  |  |
|  | Yes, in last birth |  |  | 1,345 (23.9) | 0.35 (0.28,0.42), <0.01 |  |  |
| Uterine rupture | No |  |  | 37,966 (8.2) | Reference |  |  |
|  | Yes, in an earlier birth |  |  | <5 | 0.00 (-1.61,1.61), 1.00 |  |  |
|  | Yes, in last birth |  |  | 9 (33.3) | 1.40 (0.46,2.34), <0.01 |  |  |
| Caesarean section delivery | No |  |  | 25,998 (7.3) | Reference |  |  |
|  | Yes, in an earlier birth |  |  | 929 (13.7) | 0.18 (0.12,0.24), <0.01 |  |  |
|  | Yes, in last birth |  |  | 11,051 (11.1) | 0.27 (0.26,0.29), <0.01 |  |  |
| Stillbirth | No |  |  | 36,225 (7.9) | Reference |  |  |
|  | Yes, in an earlier birth |  |  | 602 (16.0) | 0.25 (0.17,0.33), <0.01 |  |  |
|  | Yes, in last birth |  |  | 1,151 (23.6) | 0.31 (0.23,0.40), <0.01 |  |  |
| Gestational age of last birth, weeks | <28 |  |  | 1,295 (31.2) | 1.22 (1.13,1.32), <0.01 |  |  |
|  | 28-31 |  |  | 1,148 (34.3) | 1.62 (1.52,1.72), <0.01 |  |  |
|  | 32-36 |  |  | 7,308 (25.5) | 1.47 (1.44,1.51), <0.01 |  |  |
|  | ≥37 |  |  | 28,227 (6.6) | Reference |  |  |
| Small-for-gestational age | No |  |  | 29,937 (7.6) | Reference |  |  |
|  | Yes, in an earlier birth |  |  | 2,534 (11.8) | 0.17 (0.13,0.21), <0.01 |  |  |
|  | Yes, in last birth |  |  | 5,507 (11.1) | 0.43 (0.40,0.45), <0.01 |  |  |
| Congenital anomalies | Yes |  |  | 3,894 (10.1) | -0.06 (-0.09,-0.03), <0.01 |  |  |
|  | No |  |  | 34,084 (8.0) |  |  |  |
| **Parent’s birth outcomes** | | | | | | | |
| Preterm birth | Yes |  |  |  |  | 1,540 (12.1) | 0.17 (0.12,0.21), <0.01 |
|  | No |  |  |  |  | 11,355 (9.2) | Reference |
| Small-for-gestational age | Yes |  |  |  |  | 2,471 (11.2) | 0.16 (0.13,0.20), <0.01 |
|  | No |  |  |  |  | 10,424 (9.1) | Reference |
| Congenital anomalies | Yes |  |  |  |  | 660 (10.0) | 0.02 (-0.04,0.08), 0.55 |
|  | No |  |  |  |  | 12,235 (9.5) | Reference |
| **Grandmother’s chronic medical conditions and obstetric history** | | | | | | | |
| Essential hypertension | Yes |  |  |  |  | 20 (12.6) | 0.00 (-0.41,0.40), 0.98 |
|  | No |  |  |  |  | 12,875 (9.5) | Reference |
| Diabetes mellitus | Yes |  |  |  |  | 35 (19.2) | 0.28 (-0.08,0.65), 0.13 |
|  | No |  |  |  |  | 12,860 (9.5) | Reference |
| Asthma | Yes |  |  |  |  | 220 (10.6) | 0.06 (-0.05,0.17), 0.28 |
|  | No |  |  |  |  | 12,675 (9.5) | Reference |
| Miscarriage | Yes |  |  |  |  | 469 (10.5) | 0.04 (-0.03,0.11), 0.30 |
|  | No |  |  |  |  | 12,426 (9.5) | Reference |
| Circulatory system disease | Yes |  |  |  |  | 145 (11.5) | 0.16 (0.01,0.30), 0.03 |
|  | No |  |  |  |  | 12,750 (9.5) | Reference |
| Gestational diabetes | Yes |  |  |  |  | 139 (12.0) | 0.02 (-0.12,0.16), 0.79 |
|  | No |  |  |  |  | 12,756 (9.5) | Reference |
| Gestational hypertension | Yes |  |  |  |  | 13 (7.3) | -0.65 (-1.05,-0.25), <0.01 |
|  | No |  |  |  |  | 12,882 (9.5) | Reference |
| Urinary tract infection | Yes |  |  |  |  | 1,409 (11.1) | 0.02 (-0.03,0.06), 0.47 |
|  | No |  |  |  |  | 11,486 (9.3) | Reference |
| Cancer registration | Yes |  |  |  |  | 14 (9.7) | -0.09 (-0.50,0.33), 0.68 |
|  | No |  |  |  |  | 12,881 (9.5) | Reference |
| Pre-eclampsia | Yes |  |  |  |  | 1,532 (9.9) | -0.02 (-0.06,0.02), 0.39 |
|  | No |  |  |  |  | 11,363 (9.4) | Reference |
| Threatened miscarriage | Yes |  |  |  |  | 3,577 (10.4) | 0.04 (0.01,0.07), 0.02 |
|  | No |  |  |  |  | 9,318 (9.2) | Reference |
| Placenta praevia | Yes |  |  |  |  | 132 (9.6) | -0.06 (-0.19,0.08), 0.40 |
|  | No |  |  |  |  | 12,763 (9.5) | Reference |
| Placental abruption | Yes |  |  |  |  | 163 (9.5) | -0.21 (-0.33,-0.08), <0.01 |
|  | No |  |  |  |  | 12,732 (9.5) | Reference |
| Pre-labour rupture of membranes | Yes |  |  |  |  | 788 (11.8) | 0.10 (0.03,0.16), <0.01 |
|  | No |  |  |  |  | 12,107 (9.4) | Reference |
| Unspecified antepartum haemorrhage | Yes |  |  |  |  | 534 (10.2) | -0.01 (-0.08,0.06), 0.74 |
|  | No |  |  |  |  | 12,361 (9.5) | Reference |
| Stillbirth | Yes |  |  |  |  | <5 | -0.33 (-0.62,-0.04), <0.01 |
|  | No |  |  |  |  | 12,895.0 (9.5) | Reference |

β, beta coefficient; CI, confidence interval; IRSD, The Index of Relative Socio-economic Disadvantage; N/A, not applicable

Coding for pregnancy history: Nulliparous - parity=0; First birth, parity>0 – no previous birth records exist as this was the first birth in the study period for a non-nulliparous mother; No – previous birth records exist and there is no history of the condition; Yes, in an earlier birth – the condition was present in an earlier birth; Yes, in last birth – the condition was present in the birth prior to the current one; Unknown – unknown due to missing value. Results with cell size less than 5 (excluding zero) have been presented as “<5” in accordance with the practice code for the use of personal health information provided by the WA Department of Health

Model A: Cohort - all births; Predictors - maternal socio-demographic factors, maternal chronic medical conditions, and current pregnancy characteristics and complications; Model B: Cohort - births of multiparous women; Predictors - Model A + maternal past obstetric history; Model C: Cohort - births of parents who were born during the study period; Predictors - Model A + parent’s birth outcomes and grandmother’s chronic medical conditions and obstetric history

Predictive probability for preterm birth can be calculated using the following formula, where *X* is the non-reference predictor category (either a value of 1 if present or 0 if absent) and *β* is the associated beta coefficient.

$$P_{Preterm birth}= \frac{e^{intercept+ \sum_{i=1}^{n} \beta_{i}X_{i}}}{1+e^{intercept+\sum_{i=1}^{n} \beta_{i}X_{i}}}$$

**Supplementary Table 8. Estimated coefficients of models D, E and F produced using regularised logistic regression algorithms**

|  |  | **Model D (n=953,806)** | | **Model E (n=465,244)** | | **Model F (n=465,215)** | |
| --- | --- | --- | --- | --- | --- | --- | --- |
|  |  | Preterm birth n (%) | β (95% CI),  *P*-value | Preterm birth n (%) | β (95% CI),  *P*-value | Preterm birth n (%) | β (95% CI),  *P*-value |
| Intercept |  | 81,706 (8.6) | -0.34 (-0.36,-0.32), <0.01 | 38,043 (8.2) | -0.63 (-0.65,-0.60), <0.01 | 38,021 (8.2) |  |
| **Maternal socio-demographic factors** | | | | | | | |
| Age, years | <20 | 5,744 (11.0) | 0.11 (0.09,0.13), <0.01 | 1,204 (14.4) | 0.42 (0.37,0.47), <0.01 | 1,203 (14.4) | 0.52 (0.47,0.58), <0.01 |
|  | 20-24 | 14,993 (8.3) | Reference | 6,386 (9.0) | Reference | 6,383 (9.0) | Reference |
|  | 25-29 | 23,820 (7.8) | 0.00 (-0.01,0.01), 0.86 | 10,894 (7.5) | -0.13 (-0.15,-0.11), <0.01 | 10,891 (7.5) | -0.17 (-0.19,-0.14), <0.01 |
|  | 30-34 | 22,719 (8.3) | 0.10 (0.09,0.12), <0.01 | 11,612 (7.6) | -0.11 (-0.13,-0.09), <0.01 | 11,602 (7.6) | -0.16 (-0.18,-0.13), <0.01 |
|  | 35-39 | 11,676 (9.8) | 0.28 (0.26,0.29), <0.01 | 6,461 (8.8) | 0.00 (-0.02,0.02), 1.00 | 6,456 (8.8) | -0.08 (-0.11,-0.05), <0.01 |
|  | ≥40 | 2,754 (12.3) | 0.50 (0.47,0.53), <0.01 | 1,486 (11.2) | 0.19 (0.15,0.23), <0.01 | 1,486 (11.2) | 0.10 (0.06,0.15), <0.01 |
| Ethnicity | Caucasian | 64,308 (8.1) | Reference | 29,219 (7.5) | Reference | 29,205 (7.5) | Reference |
|  | Indigenous | 7,537 (15.4) | 0.66 (0.64,0.68), <0.01 | 4,894 (15.7) | 0.47 (0.44,0.50), <0.01 | 4,888 (15.7) | 0.52 (0.48,0.55), <0.01 |
|  | Other | 9,861 (8.9) | 0.05 (0.04,0.07), <0.01 | 3,930 (9.0) | 0.12 (0.10,0.15), <0.01 | 3,928 (9.0) | 0.20 (0.18,0.23), <0.01 |
| Socio-economic status, IRSD percentile | <20 | 18,172 (9.8) | 0.13 (0.11,0.14), <0.01 | 9,901 (10.1) | 0.10 (0.08,0.12), <0.01 | 9,892 (10.1) | 0.03 (0.01,0.06), 0.02 |
|  | 20-39 | 15,751 (8.6) | 0.07 (0.05,0.08), <0.01 | 7,545 (8.3) | 0.04 (0.02,0.06), <0.01 | 7,543 (8.3) | -0.01 (-0.03,0.02), 0.47 |
|  | 40-59 | 14,828 (8.2) | 0.02 (0.01,0.03), <0.01 | 6,696 (7.7) | 0.00 (-0.02,0.02), 0.98 | 6,692 (7.7) | -0.01 (-0.04,0.01), 0.25 |
|  | 60-79 | 14,868 (8.2) | 0.03 (0.02,0.05), <0.01 | 6,554 (7.6) | 0.01 (-0.01,0.03), 0.32 | 6,551 (7.5) | 0.02 (-0.01,0.04), 0.12 |
|  | ≥80 | 14,277 (8.0) | Reference | 6,269 (7.3) | Reference | 6,267 (7.3) | Reference |
|  | Unknown | 3,810 (8.3) | 0.07 (0.04,0.10), <0.01 | 1,078 (6.5) | -0.10 (-0.14,-0.05), <0.01 | 1,076 (6.5) | -0.12 (-0.17,-0.07), <0.01 |
| Remoteness of residence | Major cities | 54,103 (8.6) | Reference | 24,059 (8.1) | Reference | 24,052 (8.1) | Reference |
|  | Regional/Remote | 19,321 (8.6) | -0.09 (-0.10,-0.08), <0.01 | 10,243 (8.3) | -0.10 (-0.12,-0.08), <0.01 | 10,232 (8.3) | -0.10 (-0.12,-0.08), <0.01 |
|  | Unknown | 8,282 (8.6) | -0.05 (-0.07,-0.03), <0.01 | 3,741 (8.0) | -0.01 (-0.03,0.02), 0.54 | 3,737 (8.0) | 0.00 (-0.03,0.03), 0.87 |
| Smoking | Yes | 9,727 (11.6) | 0.26 (0.24,0.27), <0.01 | 6,116 (12.2) | 0.27 (0.24,0.29), <0.01 | 6,116 (12.2) | 0.22 (0.19,0.24), <0.01 |
|  | No | 38,042 (8.5) | Reference | 17,520 (7.9) | Reference | 17,520 (7.9) | Reference |
|  | Unknown | 33,937 (8.0) | 0.03 (0.01,0.06), <0.01 | 14,407 (7.5) | 0.04 (0.00,0.07), 0.03 | 14,385 (7.5) | 0.11 (0.07,0.14), <0.01 |
| **Maternal chronic medical conditions** | | | | | | | |
| Essential hypertension | Yes | 533 (17.1) | 0.51 (0.42,0.59), <0.01 | 512 (16.9) | 0.13 (0.05,0.22), <0.01 | 512 (16.9) | 0.18 (0.09,0.28), <0.01 |
|  | No | 81,173 (8.5) | Reference | 37,531 (8.1) | Reference | 37,509 (8.1) | Reference |
| Diabetes mellitus | Yes | 1,045 (33.3) | 1.44 (1.34,1.54), <0.01 | 770 (31.4) | 0.96 (0.85,1.07), <0.01 | 769 (31.4) | 1.23 (1.12,1.34), <0.01 |
|  | No | 80,661 (8.5) | Reference | 37,273 (8.1) | Reference | 37,252 (8.0) | Reference |
| Asthma | Yes | 4,551 (10.8) | 0.24 (0.22,0.26), <0.01 | 4,225 (10.7) | 0.14 (0.12,0.17), <0.01 | 4,221 (10.7) | 0.09 (0.06,0.11), <0.01 |
|  | No | 77,155 (8.5) | Reference | 33,818 (7.9) | Reference | 33,800 (7.9) | Reference |
| Miscarriage | Yes | 3,068 (11.6) | 0.26 (0.24,0.29), <0.01 | 3,010 (11.4) | 0.19 (0.17,0.22), <0.01 | 3,004 (11.4) | 0.08 (0.05,0.12), <0.01 |
|  | No | 78,638 (8.5) | Reference | 35,033 (8.0) | Reference | 35,017 (8.0) | Reference |
| Obesity | Yes | 796 (12.6) | 0.18 (0.12,0.23), <0.01 | 626 (13.4) | 0.09 (0.03,0.16), <0.01 | 626 (13.4) | -0.03 (-0.10,0.05), 0.45 |
|  | No | 80,910 (8.5) | Reference | 37,417 (8.1) | Reference | 37,395 (8.1) | Reference |
| Circulatory system diseases | Yes | 1,170 (13.2) | 0.30 (0.25,0.34), <0.01 | 1,009 (13.4) | 0.29 (0.24,0.34), <0.01 | 1,009 (13.4) | 0.14 (0.09,0.20), <0.01 |
|  | No | 80,536 (8.5) | Reference | 37,034 (8.1) | Reference | 37,012 (8.1) | Reference |
| **Current pregnancy characteristics and complications** | | | | | | | |
| Parity | 0 | 35,837 (9.3) | 0.30 (0.29,0.31), <0.01 | N/A |  | N/A |  |
|  | 1 | 23,808 (7.4) | Reference | 19,510 (7.4) | Reference | 19,500 (7.4) | Reference |
|  | 2 | 12,091 (7.8) | 0.00 (-0.01,0.02), 0.67 | 9,920 (7.9) | -0.01 (-0.03,0.00), 0.11 | 9,913 (7.9) | 0.01 (-0.01,0.03), 0.39 |
|  | ≥3 | 9,970 (10.9) | 0.20 (0.18,0.21), <0.01 | 8,613 (11.2) | 0.14 (0.12,0.16), <0.01 | 8,608 (11.2) | 0.14 (0.12,0.17), <0.01 |
| Gestational diabetes | Yes |  |  |  |  | 2,944 (13.7) | 0.28 (0.24,0.32), <0.01 |
|  | No |  |  |  |  | 35,077 (7.9) | Reference |
| Gestational hypertension | Yes |  |  |  |  | 1,107 (12.6) | -0.25 (-0.31,-0.20), <0.01 |
|  | No |  |  |  |  | 36,914 (8.1) | Reference |
| Urinary tract infection | Yes |  |  |  |  | 3,853 (15.2) | 0.17 (0.14,0.21), <0.01 |
|  | No |  |  |  |  | 34,168 (7.8) | Reference |
| Cancer registration | Yes |  |  |  |  | 181 (11.4) | 0.54 (0.42,0.66), <0.01 |
|  | No |  |  |  |  | 37,840 (8.2) | Reference |
| Pre-eclampsia | Yes |  |  |  |  | 4,249 (21.9) | 1.34 (1.31,1.38), <0.01 |
|  | No |  |  |  |  | 33,772 (7.6) | Reference |
| Threatened miscarriage | Yes |  |  |  |  | 17,213 (18.3) | 1.04 (1.02,1.05), <0.01 |
|  | No |  |  |  |  | 20,808 (5.6) | Reference |
| Placenta praevia | Yes |  |  |  |  | 1,730 (33.2) | 1.96 (1.88,2.03), <0.01 |
|  | No |  |  |  |  | 36,291 (7.9) | Reference |
| Placental abruption | Yes |  |  |  |  | 1,955 (49.9) | 2.37 (2.26,2.48), <0.01 |
|  | No |  |  |  |  | 36,066 (7.8) | Reference |
| Pre-labour rupture of membranes | Yes |  |  |  |  | 9,909 (38.1) | 2.26 (2.23,2.30), <0.01 |
|  | No |  |  |  |  | 28,112 (6.4) | Reference |
| Unspecified antepartum haemorrhage | Yes |  |  |  |  | 4,507 (25.5) | 0.99 (0.95,1.03), <0.01 |
|  | No |  |  |  |  | 33,514 (7.5) | Reference |
| Threatened preterm labour | Yes |  |  |  |  | 3,959 (48.9) | 2.21 (2.13,2.28), <0.01 |
|  | No |  |  |  |  | 34,062 (7.5) | Reference |
| Uterine rupture | Yes |  |  |  |  | 19 (26.4) | 1.01 (0.43,1.58), <0.01 |
|  | No |  |  |  |  | 38,002 (8.2) | Reference |
| Birth year | 1980-1984 | 8,080 (7.5) | -0.12 (-0.15,-0.09), <0.01 | 1,919 (7.3) | 0.00 (-0.04,0.05), 0.85 | 1,918 (7.3) | -0.09 (-0.14,-0.03), <0.01 |
|  | 1985-1989 | 9,664 (7.9) | -0.08 (-0.10,-0.05), <0.01 | 4,410 (7.1) | -0.06 (-0.10,-0.02), <0.01 | 4,409 (7.1) | -0.11 (-0.16,-0.06), <0.01 |
|  | 1990-1994 | 10,344 (8.2) | -0.07 (-0.10,-0.05), <0.01 | 5,144 (7.5) | -0.04 (-0.08,0.00), 0.06 | 5,139 (7.5) | -0.15 (-0.19,-0.10), <0.01 |
|  | 1995-1999 | 10,859 (8.5) | -0.07 (-0.09,-0.05), <0.01 | 5,446 (7.9) | -0.07 (-0.09,-0.04), <0.01 | 5,442 (7.9) | -0.17 (-0.20,-0.13), <0.01 |
|  | 2000-2004 | 11,301 (9.1) | -0.03 (-0.04,-0.01), <0.01 | 5,810 (8.7) | -0.01 (-0.03,0.01), 0.52 | 5,809 (8.7) | -0.18 (-0.20,-0.15), <0.01 |
|  | 2005-2009 | 13,474 (9.2) | -0.01 (-0.02,0.00), 0.18 | 6,673 (8.8) | -0.03 (-0.05,-0.01), <0.01 | 6,669 (8.8) | -0.16 (-0.18,-0.13), <0.01 |
|  | 2010-2015 | 17,984 (9.1) | Reference | 8,641 (8.8) | Reference | 8,635 (8.8) | Reference |
| Plurality | Singleton |  |  |  |  | 30,876 (6.8) | Reference |
|  | Twin |  |  |  |  | 6,769 (53.0) | 2.76 (2.70,2.83), <0.01 |
|  | Multiple (>2) |  |  |  |  | 376 (96.9) | 5.62 (3.98,7.27), <0.01 |
| Small-for-gestational age | Yes |  |  |  |  |  |  |
|  | No |  |  |  |  |  |  |
| Congenital anomalies | Yes |  |  |  |  |  |  |
|  | No |  |  |  |  |  |  |
| **Past obstetric history** | | | | | | | |
| Gestational diabetes | No |  |  | 36,270 (8.0) | Reference | 36,249 (8.0) | Reference |
|  | Yes, in an earlier birth |  |  | 276 (12.7) | 0.00 (-0.10,0.10), 1.00 | 276 (12.7) | -0.10 (-0.21,0.01), 0.08 |
|  | Yes, in last birth |  |  | 1,497 (12.1) | 0.19 (0.15,0.23), <0.01 | 1,496 (12.1) | 0.11 (0.07,0.16), <0.01 |
| Gestational hypertension | No |  |  | 36,442 (8.1) | Reference | 36,420 (8.1) | Reference |
|  | Yes, in an earlier birth |  |  | 547 (14.1) | 0.26 (0.19,0.33), <0.01 | 547 (14.1) | 0.05 (-0.04,0.13), 0.28 |
|  | Yes, in last birth |  |  | 1,054 (10.0) | 0.08 (0.04,0.12), <0.01 | 1,054 (10.0) | 0.06 (0.01,0.11), 0.02 |
| Urinary tract infection | No |  |  | 32,559 (7.7) | Reference | 32,543 (7.7) | Reference |
|  | Yes, in an earlier birth |  |  | 2,048 (13.2) | 0.06 (0.02,0.10), <0.01 | 2,046 (13.2) | 0.00 (-0.05,0.04), 0.84 |
|  | Yes, in last birth |  |  | 3,436 (12.2) | 0.16 (0.14,0.19), <0.01 | 3,432 (12.2) | 0.05 (0.02,0.08), <0.01 |
| Cancer registration | No |  |  | 37,893 (8.2) | Reference | 37,871 (8.2) | Reference |
|  | Yes, in an earlier birth |  |  | 48 (12.9) | 0.00 (-0.23,0.23), 1.00 | 48 (12.9) | 0.05 (-0.21,0.31), 0.70 |
|  | Yes, in last birth |  |  | 102 (9.6) | 0.00 (-0.13,0.13), 1.00 | 102 (9.6) | 0.00 (-0.15,0.15), 1.00 |
| Pre-eclampsia | No |  |  | 32,137 (7.7) | Reference | 32,116 (7.7) | Reference |
|  | Yes, in an earlier birth |  |  | 1,646 (11.2) | 0.13 (0.10,0.17), <0.01 | 1,645 (11.2) | 0.10 (0.06,0.14), <0.01 |
|  | Yes, in last birth |  |  | 4,260 (12.4) | 0.36 (0.33,0.38), <0.01 | 4,260 (12.4) | 0.04 (0.01,0.07), <0.01 |
| Threatened miscarriage | No |  |  | 23,025 (6.9) | Reference | 23,011 (6.9) | Reference |
|  | Yes, in an earlier birth |  |  | 3,763 (11.3) | 0.15 (0.12,0.18), <0.01 | 3,762 (11.3) | 0.17 (0.14,0.20), <0.01 |
|  | Yes, in last birth |  |  | 11,255 (11.7) | 0.18 (0.17,0.20), <0.01 | 11,248 (11.7) | 0.07 (0.05,0.09), <0.01 |
| Placenta praevia | No |  |  | 37,302 (8.1) | Reference | 37,281 (8.1) | Reference |
|  | Yes, in an earlier birth |  |  | 228 (15.5) | 0.16 (0.04,0.27), <0.01 | 227 (15.4) | 0.05 (-0.08,0.18), 0.42 |
|  | Yes, in last birth |  |  | 513 (13.1) | 0.00 (-0.07,0.07), 1.00 | 513 (13.1) | -0.16 (-0.24,-0.07), <0.01 |
| Placental abruption | No |  |  | 36,712 (8.0) | Reference | 36,690 (8.0) | Reference |
|  | Yes, in an earlier birth |  |  | 437 (19.4) | 0.30 (0.20,0.40), <0.01 | 437 (19.4) | 0.34 (0.24,0.45), <0.01 |
|  | Yes, in last birth |  |  | 894 (21.4) | 0.33 (0.26,0.41), <0.01 | 894 (21.4) | 0.16 (0.08,0.24), <0.01 |
| Pre-labour rupture of membranes | No |  |  | 30,896 (7.4) | Reference | 30,879 (7.4) | Reference |
|  | Yes, in an earlier birth |  |  | 1,979 (14.3) | 0.33 (0.29,0.36), <0.01 | 1,977 (14.3) | 0.24 (0.19,0.28), <0.01 |
|  | Yes, in last birth |  |  | 5,168 (15.7) | 0.36 (0.34,0.39), <0.01 | 5,165 (15.7) | 0.01 (-0.02,0.04), 0.68 |
| Unspecified antepartum haemorrhage | No |  |  | 34,311 (7.8) | Reference | 34,292 (7.8) | Reference |
|  | Yes, in an earlier birth |  |  | 1,264 (15.8) | 0.29 (0.24,0.34), <0.01 | 1,263 (15.8) | 0.17 (0.11,0.22), <0.01 |
|  | Yes, in last birth |  |  | 2,468 (15.1) | 0.36 (0.32,0.39), <0.01 | 2,466 (15.1) | 0.16 (0.12,0.20), <0.01 |
| Threatened preterm labour | No |  |  | 36,201 (7.9) | Reference | 36,179 (7.9) | Reference |
|  | Yes, in an earlier birth |  |  | 495 (20.9) | 0.45 (0.35,0.55), <0.01 | 495 (20.9) | 0.43 (0.33,0.53), <0.01 |
|  | Yes, in last birth |  |  | 1,347 (24.0) | 0.49 (0.42,0.56), <0.01 | 1,347 (24.0) | 0.34 (0.27,0.41), <0.01 |
| Uterine rupture | No |  |  | 38,031 (8.2) | Reference | 38,009 (8.2) | Reference |
|  | Yes, in an earlier birth |  |  | <5 | 0.00 (-1.24,1.24), 1.00 | <5 | 0.00 (-1.67,1.67), 1.00 |
|  | Yes, in last birth |  |  | 9 (33.3) | 0.00 (-0.89,0.89), 1.00 | 9 (33.3) | 1.49 (0.60,2.39), <0.01 |
| Caesarean section delivery | No |  |  | 26,044 (7.3) | Reference | 26,029 (7.3) | Reference |
|  | Yes, in an earlier birth |  |  | 934 (13.8) | 0.22 (0.16,0.27), <0.01 | 933 (13.8) | 0.19 (0.13,0.25), <0.01 |
|  | Yes, in last birth |  |  | 11,065 (11.1) | 0.38 (0.36,0.40), <0.01 | 11,059 (11.1) | 0.29 (0.27,0.30), <0.01 |
| Stillbirth | No |  |  | 36,286 (7.9) | Reference | 36,264 (7.9) | Reference |
|  | Yes, in an earlier birth |  |  | 606 (16.1) | 0.14 (0.06,0.21), <0.01 | 606 (16.1) | 0.23 (0.15,0.31), <0.01 |
|  | Yes, in last birth |  |  | 1,151 (23.6) | 0.18 (0.09,0.26), <0.01 | 1,151 (23.6) | 0.31 (0.22,0.40), <0.01 |
| Gestational age of last birth, weeks | <28 |  |  | 1,298 (31.2) | 1.35 (1.25,1.45), <0.01 | 1,297 (31.2) | 1.24 (1.14,1.34), <0.01 |
|  | 28-31 |  |  | 1,149 (34.3) | 1.45 (1.35,1.55), <0.01 | 1,148 (34.3) | 1.62 (1.52,1.72), <0.01 |
|  | 32-36 |  |  | 7,321 (25.5) | 1.28 (1.25,1.32), <0.01 | 7,321 (25.5) | 1.45 (1.42,1.48), <0.01 |
|  | ≥37 |  |  | 28,275 (6.6) | Reference | 28,255 (6.6) | Reference |
| Small-for-gestational age | No |  |  | 29,987 (7.6) | Reference | 29,970 (7.6) | Reference |
|  | Yes, in an earlier birth |  |  | 2,542 (11.8) | 0.10 (0.07,0.13), <0.01 | 2,540 (11.8) | 0.17 (0.13,0.20), <0.01 |
|  | Yes, in last birth |  |  | 5,514 (11.1) | 0.35 (0.33,0.37), <0.01 | 5,511 (11.1) | 0.40 (0.37,0.42), <0.01 |
| Congenital anomalies | Yes |  |  | 3,906 (10.1) | -0.01 (-0.03,0.02), 0.63 | 3,902 (10.1) | -0.04 (-0.07,-0.02), <0.01 |
|  | No |  |  | 34,137 (8.0) | Reference | 34,119 (8.0) | Reference |

β, beta coefficient; CI, confidence interval; IRSD, The Index of Relative Socio-economic Disadvantage; N/A, not applicable

Coding for pregnancy history: Nulliparous - parity=0; First birth, parity>0 – no previous birth records exist as this was the first birth in the study period for a non-nulliparous mother; No – previous birth records exist and there is no history of the condition; Yes, in an earlier birth – the condition was present in an earlier birth; Yes, in last birth – the condition was present in the birth prior to the current one; Unknown – unknown due to missing value. Results with cell size less than 5 (excluding zero) have been presented as “<5” in accordance with the practice code for the use of personal health information provided by the WA Department of Health

Model D: Cohort - all births; Predictors - maternal socio-demographic factors, maternal chronic medical conditions, parity, and birth year; Model E: Cohort - births of multiparous women; Predictors - Model D + maternal past obstetric history; Model F: Cohort - births of multiparous women; Predictors - Model B excluding small-for-gestational age and congenital anomalies in current birth

Predictive probability for preterm birth can be calculated using the following formula, where *X* is the non-reference predictor category (either a value of 1 if present or 0 if absent) and *β* is the associated beta coefficient.

$$P_{Preterm birth}= \frac{e^{intercept+ \sum_{i=1}^{n} \beta_{i}X_{i}}}{1+e^{intercept+\sum_{i=1}^{n} \beta_{i}X_{i}}}$$
